# Abdominal CT metrics in 17,646 patients reveal associations between myopenia, myosteatosis, and medical phenotypes: a phenome-wide association study

**DOI:** 10.1016/j.ebiom.2024.105116

**Published:** 2024-04-17

**Authors:** Juan M. Zambrano Chaves, Leon Lenchik, Isabel O. Gallegos, Louis Blankemeier, Daniel L. Rubin, Marc H. Willis, Akshay S. Chaudhari, Robert D. Boutin

**Affiliations:** a Department of Biomedical Data Science, Stanford University School of Medicine, Stanford, CA, USA; b Department of Diagnostic Radiology, Wake Forest University School of Medicine, Winston-Salem, North Carolina, USA; c Department of Computer Science, (IOG), Stanford University, Stanford, CA, USA; d Department of Electrical Engineering, Stanford University, Stanford, CA, USA; e Department of Radiology, Stanford University, Stanford, CA, USA

**Keywords:** Myopenia, Myosteatosis, Phenome wide association study, Computed tomography

## Abstract

**Background:**

Deep learning facilitates large-scale automated imaging evaluation of body composition. However, associations of body composition biomarkers with medical phenotypes have been underexplored. Phenome-wide association study (PheWAS) techniques search for medical phenotypes associated with biomarkers. A PheWAS integrating large-scale analysis of imaging biomarkers and electronic health record (EHR) data could discover previously unreported associations and validate expected associations. Here we use PheWAS methodology to determine the association of abdominal CT-based skeletal muscle metrics with medical phenotypes in a large North American cohort.

**Methods:**

An automated deep learning pipeline was used to measure skeletal muscle index (SMI; biomarker of myopenia) and skeletal muscle density (SMD; biomarker of myosteatosis) from abdominal CT scans of adults between 2012 and 2018. A PheWAS was performed with logistic regression using patient sex and age as covariates to assess for associations between CT-derived muscle metrics and 611 common EHR-derived medical phenotypes. PheWAS P values were considered significant at a Bonferroni corrected threshold (α = 0.05/1222).

**Findings:**

17,646 adults (mean age, 56 years ± 19 [SD]; 57.5% women) were included. CT-derived SMI was significantly associated with 268 medical phenotypes; SMD with 340 medical phenotypes. Previously unreported associations with the highest magnitude of significance included higher SMI with decreased cardiac dysrhythmias (OR [95% CI], 0.59 [0.55–0.64]; P < 0.0001), decreased epilepsy (OR, 0.59 [0.50–0.70]; P < 0.0001), and increased elevated prostate-specific antigen (OR, 1.84 [1.47–2.31]; P < 0.0001), and higher SMD with decreased decubitus ulcers (OR, 0.36 [0.31–0.42]; P < 0.0001), sleep disorders (OR, 0.39 [0.32–0.47]; P < 0.0001), and osteomyelitis (OR, 0.43 [0.36–0.52]; P < 0.0001).

**Interpretation:**

PheWAS methodology reveals previously unreported associations between CT-derived biomarkers of myopenia and myosteatosis and EHR medical phenotypes. The high-throughput PheWAS technique applied on a population scale can generate research hypotheses related to myopenia and myosteatosis and can be adapted to research possible associations of other imaging biomarkers with hundreds of EHR medical phenotypes.

**Funding:**

10.13039/100000002National Institutes of Health, Stanford AIMI-HAI pilot grant, Stanford Precision Health and Integrated Diagnostics, 10.13039/100019607Stanford Cardiovascular Institute, Stanford Center for Digital Health, and Stanford Knight-Hennessy Scholars.

**Research in context**

**Evidence before this study**

We searched PubMed and the Cochrane Library from database inception to October 22, 2023 using keywords “phenome-wide association studies (PheWAS)” AND “muscle”, “myopenia”, “myosteatosis”, “body composition”, “computed tomography (CT)”, or “diagnostic imaging” with no language restrictions. Previous PheWAS studies have evaluated the association of genetic features with observable phenotypes, and fully automated approaches can now combine deep learning-based computer vision analysis of routinely acquired medical images with known EHR-based medical phenotypes. Although PheWAS methodology can now be used to study the association between imaging metrics and disease phenotypes, no studies have used PheWAS methodology to study imaging markers of the largest protein reservoir in the human body, skeletal muscle.

**Added value of this study**

In this study, we apply PheWAS methodology to integrate large-scale analysis of CT muscle metrics and EHR data to determine the association of abdominal CT-based skeletal muscle of mass (skeletal muscle index [SMI], used to assess for myopenia) and quality (skeletal muscle density [SMD], used to assess for myosteatosis) with medical phenotypes in a retrospective cohort of 17,646 North American adults. Of 611 medical phenotypes, 213 were significantly associated with both SMI and SMD. Both the effect size and statistical significance for each association of SMI and SMD with the EHR-derived medical phenotypes are determined and supported with multimedia interactive displays after Bonferroni correction. We identify the presence and magnitude of previously unreported phenotype associations for both SMI and SMD. For example, we find previously unreported associations of higher SMI with decreased cardiac dysrhythmias (OR, 0.59; P < 0.0001) and decreased epilepsy (OR, 0.59; P < 0.0001). We also find expected associations that have been reported in the literature and are validated by our PheWAS approach, such as higher SMI associated with decreased protein-calorie malnutrition (OR, 0.30 [0.28–0.32]; P < 0.0001) and higher SMD associated with decreased morbid obesity (OR, 0.28 [0.26–0.31]; P < 0.0001).

**Implications of all the available evidence**

Our study shows that deep learning can be fully automated to analyse routinely acquired CT scans for body composition alterations indicative of myopenia and myosteatosis, and this affords the opportunity to evaluate for a wide variety of disease associations at a population level. When significant associations are identified with PheWAS, hypothesis-generating analysis can focus on distinct biological mechanisms of disease and specific causal effects of imaging biomarkers on disease phenotypes. With appropriate replication and validation in independent populations, the application of biological knowledge to diagnostic imaging in a standardized high-throughput PheWAS framework could enable accelerated evaluation of previously unreported strategies for disease prevention and management, such as pharmaceutical repositioning of existing drugs to treat diseases that share common pathophysiology.

## Introduction

Myopenia (low muscle mass) and myosteatosis (accumulation of lipid in muscle) have been associated with higher risk of adverse patient outcomes, including fragility fractures, adverse cardiovascular events, and death.^1^ With CT, myopenia and myosteatosis are quantified using surrogate measures of muscle mass (e.g., skeletal muscle index, SMI) and quality (e.g., skeletal muscle density, SMD).^2^ Fully automated approaches measuring SMI and SMD using traditional and deep learning-based computer vision methods^3^, ^4^, ^5^, ^6^, ^7^ have revolutionized research on myopenia and myosteatosis.^8^, ^9^, ^10^ More importantly, the application of automated segmentation methods to clinical CT scans may “opportunistically” improve patient care by quantifying image metrics beyond those in the original reason for examination. Identifying patients at risk for myopenia and myosteatosis on CT scans obtained for other reasons may lead to early interventions with diet and exercise, and in the future with pharmacologic therapy, thereby reducing morbidity and mortality.

As in many other diseases, research on myopenia has benefited from genome-wide association studies that enable the identification of single nucleotide variants associated with myopenia.^11^ Research using genome-wide association studies has led to research using *phenome*-wide association studies (PheWAS) that search for phenotypes associated with specific single nucleotide variants.^12^ Hundreds of PheWAS, mostly focused on genomic biomarkers, have been previously carried out^13^; however, this methodology has not been used to study myopenia and myosteatosis.

Recently, in place of genotypes, PheWAS methodology has been adapted to study imaging metrics or laboratory measurements for deriving novel hypotheses about disease associations in large populations.^8^^,^^14^^,^^15^ To our knowledge, no studies have used PheWAS methodology to study imaging markers of myopenia or myosteatosis. In this study, we apply PheWAS methodology to integrate large-scale analysis of CT muscle metrics and EHR data. The aim of the study was to systematically determine the association of abdominal CT-based skeletal muscle metrics with medical phenotypes using PheWAS methodology in a large North American cohort.

## Methods

### Overview

**Fig. 1:**
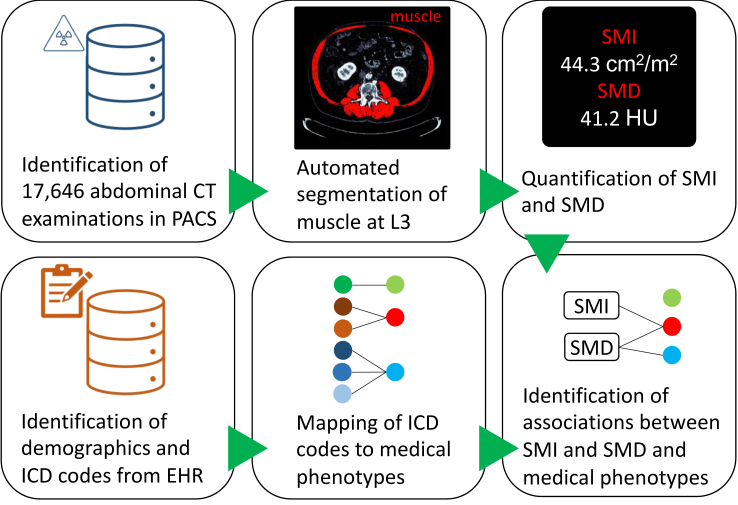
Diagram outlines muscle PheWAS study design. CT, computed tomography; PACS, picture archiving and communications system; L3, axial slice at third lumbar vertebra; SMI, skeletal muscle index; SMD, skeletal muscle density; ICD, International Classification of Diseases; EHR, electronic health record.

An overview of the study methodology is presented in Fig. 1. Briefly, consecutive abdominal CT examinations of adults along with their medical records were retrospectively collected. SMI and SMD metrics were obtained using a previously validated automated deep learning pipeline. Diagnosis codes were mapped to medical phenotypes. PheWAS methodology was used to identify associations between CT metrics and medical phenotypes.

### Data sources

**Fig. 2:**
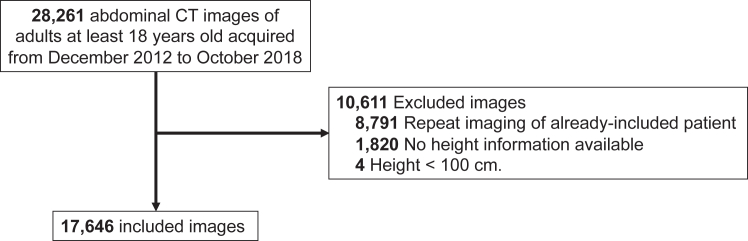
Flowchart details study inclusion and exclusion criteria and process. CT, computed tomography.

We identified consecutive patients over the age of 18 years who had intravenous contrast-enhanced abdominal CT scans at Stanford Health Care (an academic medical centre providing tertiary-level care) between December 2012 and October 2018. We excluded patients for whom no height information was available in the electronic health record (EHR), patients with height <100 cm, as well as any repeated scans on patients, such that only the earliest scan for each patient was included. From the original set of 28,261 CT scans, 8791 were excluded as repeat examinations of an already included patient, 4 from individuals with height <100 cm, and 1820 were excluded due to missing height information in the patient's EHR that is required for SMI calculation (Fig. 2). Information for all patients derived from the EHR included: age, sex, race and ethnicity, height, weight, body mass index (BMI), and International Classification of Diseases (ICD-9 and -10) codes. No EHR derived information was missing for the included patients in this study.

CT scans were performed on 17 multislice CT scanners made by four manufacturers (9918 scans on GE scanners, 7724 on Siemens, 2 on Philips, and 2 on Toshiba). Prior multi-institutional work with CT phantom evaluation of 67,392 American College of Radiology accreditation examinations has demonstrated that muscle density surrogates had only a small degree of change across CT scanners made by these four manufacturers.^16^ All scanners in this study underwent daily calibration using a quality assurance phantom, in accordance with the quality assurance specifications of the American College of Radiology, with contemporaneous daily recording of all calibration results. CT protocol parameters included a tube voltage mode of 120 kV (range, 70–140 kV), slice thickness mode of 1.25 mm (range, 0.5–5 mm), standard rotation time of 0.5s, effective current setting on the basis of body mass index, and variable contrast phase. We used a rule-based algorithm to identify the delayed contrast phase for each scan.^17^

### CT-derived muscle metrics

An automated pipeline that was previously validated at our institution was used to obtain CT-derived skeletal muscle metrics where an axial slice at the level of the third lumbar vertebra (L3) was selected using a deep learning algorithm consisting of a two-dimensional fully-convolutional neural network.^18^ This axial slice was then input into a second deep learning algorithm, a 2.5-dimensional convolutional neural network, which labelled pixels as muscle or non-muscle. Muscle cross-sectional area in cm^2^ and average radiodensity (SMD) in Hounsfield units (HU) were obtained from each image. The cross-sectional area was divided twice by the patient's height in meters to derive the skeletal muscle index (SMI [cm^2^/m^2^]). Sex-specific mean and standard deviations of SMI and SMD were computed for males (n = 7492) and females (n = 10,154). Sex-specific z-scores for SMI and SMD were created by normalizing each patient's metrics by subtracting the corresponding sex-specific mean and dividing by the sex-specific standard deviation. This tool was utilized given prior validation yielded average measurement errors <1% for both SMI and SMD.^18^

### PheWAS

A PheWAS was performed using previously described methodology.^19^ The association between sex-specific z-scores of CT-derived SMI and SMD and phenotypes of interest was determined. The EHR ICD-9 and ICD-10 CM codes for each patient between one month prior to and three months following the time of CT acquisition were recorded. ICD-9 and -10 CM codes were grouped into phecodes, which are medical phenotypes defined by medical findings, symptoms, and diagnoses. To do so, each ICD-9 and ICD-10 CM code was mapped to a phecode using the Phecode Map v1.2.^20^^,^^21^ The Phecode Map includes medical phenotype groupings consisting of 16 categories: infectious diseases, neoplasms, endocrine/metabolic, hematopoietic, mental disorders, neurological, sense organs, circulatory system, respiratory, digestive, genitourinary, dermatologic, musculoskeletal, congenital anomalies, symptoms, and injuries & poisonings. Phecodes with a count of 2 or more for an individual corresponded to a case for that medical phenotype and a count of 0 corresponded to a control; patients with a phecode count of 1 were not included in the association study for that medical phenotype. 1206 phecodes with fewer than 100 cases or controls were excluded from analysis. Logistic regression using patient age and sex as covariates was used to determine associations between SMI and SMD z-scores and each phecode.

Nonlinear associations between SMI, SMD and medical phenotypes were also explored. Specifically a nonlinear term (SMI∗SMI and SMD∗SMD) was included and assessed for significance at the Bonferroni corrected threshold. For significant associations of the nonlinear term, likelihood ratio tests were used to determine whether the model containing the nonlinear term is preferred to the linear only model.

The code to reproduce the PheWAS analysis is available at the following website: https://github.com/StanfordMIMI/muscle_phewas/muscle_phewas.R.

### Statistical analysis

Statistical analyses were performed in R version 4.1.1 (R Core Team). Demographic characteristics of the study population were compared using the Welch's t-test for continuous variables, and the two-proportion z-test for proportions using a level of significance α = 0.05. SMI and SMD were compared across age and sex using bootstrapped two-factor ANOVA by sampling with replacement 1000 times, due to heteroskedasticity as determined by Levene's test. PheWAS analyses were performed with the R PheWAS package, version 0.99.5–5.^19^ PheWAS P values were considered significant at the Bonferroni corrected threshold (0.05/1222) and are reported as Bonferroni corrected P values (P value ∗ 1222). As an additional criteria for significance, associations were considered significant if, in addition to being significant at the Bonferroni corrected threshold, the value 1.0 was not present in the 95% confidence interval of the association's odds ratio. Associations meeting both criteria were considered significant.

### Role of the funding source

The funding sources used in this study did not have any involvement in the study design, collection, analysis, interpretation of data, writing of the report or the decision to submit the paper for publication.

### Ethics

This study was approved by Stanford University institutional review board (IRB-58903) which waived of informed consent given minimal risk to subjects. The study complied with the stipulations set forth by the institutional review board.

## Results

### Patient characteristics

**Fig. 3:**
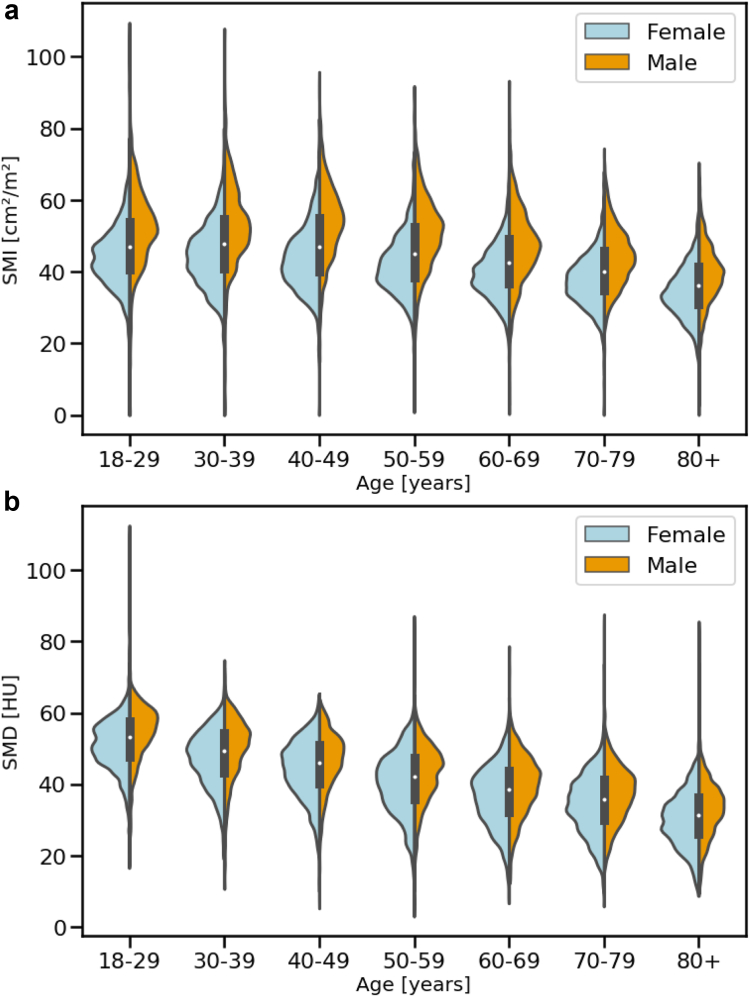
Distributions of SMI (a) and SMD (b) stratified by age decile and sex illustrate differences across subgroups. In each age group the lower black box edge, white circle, and upper black box edge mark the 25th, 50th, and 75th percentile, respectively. SMI, skeletal muscle index; SMD, skeletal muscle density.

Table 1 shows the demographic characteristics of the study population. A total of 17,646 patients were included in this analysis, with an average (standard deviation) age of 56.2 (18.3) years. There were 10,154/17,646 (57.5%) female patients. Patients were approximately evenly distributed across inpatient. (5757/17,646; 32.6%), emergency department (5705/17,646; 32.3%) and outpatient (6184/17,646; 35.1%) populations. The average (standard deviation) SMI and SMD were 44.3 (10.6) cm^2^/m^2^ and 41.2 (10.4) HU, respectively. Both SMI and SMD varied across age decile (P < 0.0001 [Bootstrap ANOVA]) and sex (P < 0.0001 [Bootstrap ANOVA]), with the interaction of age decile and sex significant for SMI (P < 0.0001 [Bootstrap ANOVA]) but not SMD (P = 0.59 [Bootstrap ANOVA]). Compared to men, women had a higher BMI (27.3 vs 26.8; P < 0.0001 [Welch's t-test]), lower SMI (41.0 vs 48.9; P < 0.0001 [Welch's t-test]), and lower SMD (40.0 vs 42.8; P < 0.0001 [Welch's t-test]). The decreases in SMI and SMD with increasing age in men and women are shown in Table 2 and Fig. 3.

**Table 1:** Demographic and other characteristics of the study population of 17,646 patients.

Parameter	Overall	Male	Female	P value
Age [years]	56.2 (18.3)	56.5 (18.0)	56.0 (18.5)	0.10
Female sex	10,154 (57.5)^a^			
Race and ethnicity				
Asian	2593 (14.7)^a^	1113 (14.9)	1480 (14.6)	0.83
Hispanic/Latino	3506 (19.9)^a^	1289 (17.2)	2217 (21.8)	0.0011
Native American	58 (0.3)^a^	24 (0.3)	34 (0.3)	1.0
Non-Hispanic Black	880 (5.0)^a^	338 (4.5)	542 (5.3)	0.60
Non-Hispanic White	8850 (50.2)^a^	3981 (53.1)	4869 (48.0)	<0.0001
Pacific Islander	254 (1.4)^a^	79 (1.1)	175 (1.7)	0.71
Other^b^	1269 (7.2)^a^	565 (7.5)	704 (6.9)	0.68
Unknown	236 (1.3)^a^	103 (1.4)	133 (1.3)	0.95
Body mass index [kg/m^2^]	27.1 (7.0)	26.8 (5.9)	27.3 (7.6)	<0.0001
SMI [cm^2^/m^2^]	44.3 (10.6)	48.9 (11.1)	41.0 (8.9)	<0.0001
SMD [HU]	41.2 (10.4)	42.8 (10.2)	40.0 (10.3)	<0.0001

Note.–P values correspond to differences in means or proportions in males vs females as determined by a t-test and two-proportion z-test, respectively. Data are presented as mean (standard deviation) unless otherwise specified. SMI, skeletal muscle index; SMD, skeletal muscle density; HU, Hounsfield unit.

a Values correspond to number (percentage), where the number corresponds to numerator and percentage is calculated with denominator 17,646.

b Documented as Other in the patient's health record.

**Table 2:** Muscle CT-metrics according to age and sex groups.

Age [years]	Number of females	Number of males	SMI females [cm^2^/m^2^]	SMI males [cm^2^/m^2^]	SMD females [HU]	SMD males [HU]
18–29	1001	740	44.1 (8.4)	52.1 (12.0)	50.8 (7.2)	54.2 (8.5)
30–39	1257	849	44.6 (9.1)	53.3 (11.3)	47.0 (8.0)	50.1 (8.5)
40–49	1583	1015	43.7 (9.1)	54.0 (10.7)	44.1 (8.2)	46.7 (8.0)
50–59	1897	1449	41.8 (8.4)	50.9 (10.5)	40.1 (8.7)	42.9 (8.1)
60–69	1920	1582	40.0 (7.8)	47.3 (10.0)	36.4 (8.6)	39.6 (8.5)
70–79	1428	1159	37.6 (7.2)	44.3 (8.3)	34.0 (8.3)	37.2 (7.9)
80+	1068	698	34.2 (7.5)	39.5 (8.2)	29.9 (7.9)	33.0 (7.7)

Note.–SMI and SMD are reported as mean (standard deviation). SMI, skeletal muscle index; SMD, skeletal muscle density; HU, Hounsfield unit.

Table 3 lists the prevalence of medical phenotypes for the 16 diagnostic groups. Among grouped medical phenotypes, *symptoms* had the highest average prevalence (1343/17,270; 7.6%), whereas *congenital anomalies* had the lowest (128/17,576; 0.7%).

**Table 3:** Prevalence of grouped medical phenotypes in study population.

Group	Number of medical phenotypes	Average number of cases/Total number of patients	Prevalence (%) [95% CI]
Infectious diseases	23	375/16,788	2.2 [2.0–2.4]
Neoplasms	65	412/14,611	2.9 [2.7–3.2]
Endocrine/metabolic	63	553/16,207	3.3 [3.1–3.5]
Hematopoietic	24	532/15,815	3.3 [3.1–3.6]
Mental disorders	27	616/15,921	3.8 [3.6–4.1]
Neurological	28	430/16,860	2.5 [2.3–2.7]
Sense organs	17	255/17,461	1.5 [1.3–1.6]
Circulatory system	81	591/15,776	3.7 [3.4–3.9]
Respiratory	35	535/16,622	3.2 [3.0–3.4]
Digestive	87	598/15,410	3.9 [3.6–4.2]
Genitourinary	50	422/13,721	3.0 [2.7–3.3]
Dermatologic	27	232/17,264	1.3 [1.2–1.5]
Musculoskeletal	31	288/17,243	1.7 [1.5–1.8]
Congenital anomalies	3	128/17,576	0.7 [0.6–0.9]
Symptoms	19	1343/17,270	7.6 [7.4–7.9]
Injuries & poisonings	31	331/16,850	1.9 [1.8–2.1]

CI, confidence interval.

### PheWAS analysis

PheWAS analysis included 611 medical phenotypes. Table S1 shows the full list of medical phenotypes studied and the magnitude of association to CT muscle metrics. CT-derived SMI was significantly associated (P < 0.05/1222) with 268 medical phenotypes and SMD with 340 medical phenotypes. 213 medical phenotypes were significantly associated (P < 0.05/1222) with both SMI and SMD. All associations significant at the Bonferroni corrected threshold were considered significant based on the associated odds ratio confidence interval. For SMI and SMD, 143 and 122 associations met the confidence interval criterion but did not meet the Bonferroni threshold criterion.

**Fig. 4:**
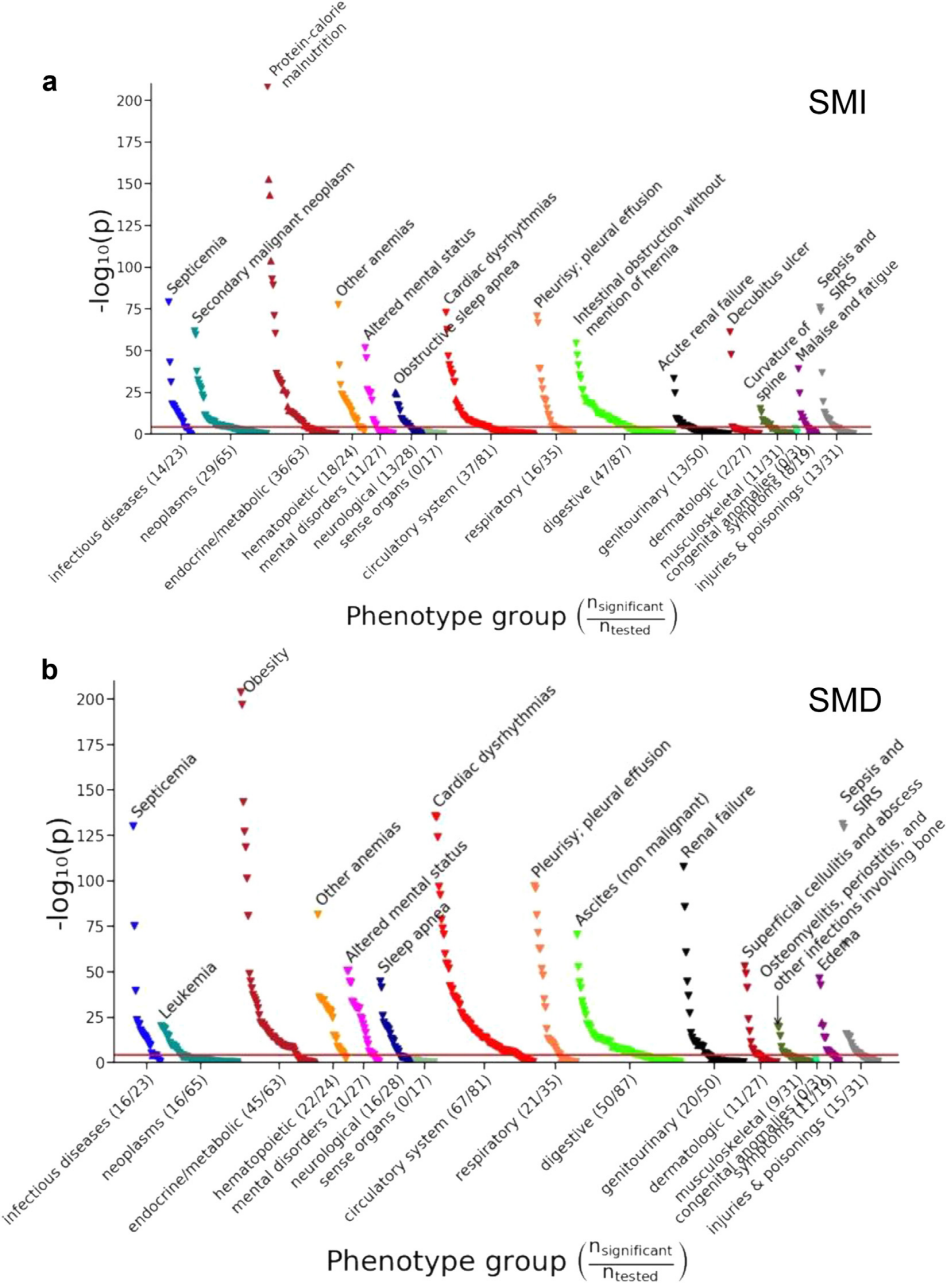
Manhattan plot for PheWAS analysis studying associations between SMI (a) and SMD (b) and ICD-derived medical phenotypes. The x-axis shows the medical phenotype groups evaluated; the y-axis shows the magnitude of significance as measured by the negative logarithm of the P value. The horizontal red line corresponds to the Bonferroni significance threshold (0.05/1222). The direction of the markers indicates the direction of the association (e.g., marker pointing up indicates higher odds with increased muscle metric). SMI, skeletal muscle index; SMD, skeletal muscle density; ICD, International Classification of Diseases.

**Fig. 5:**
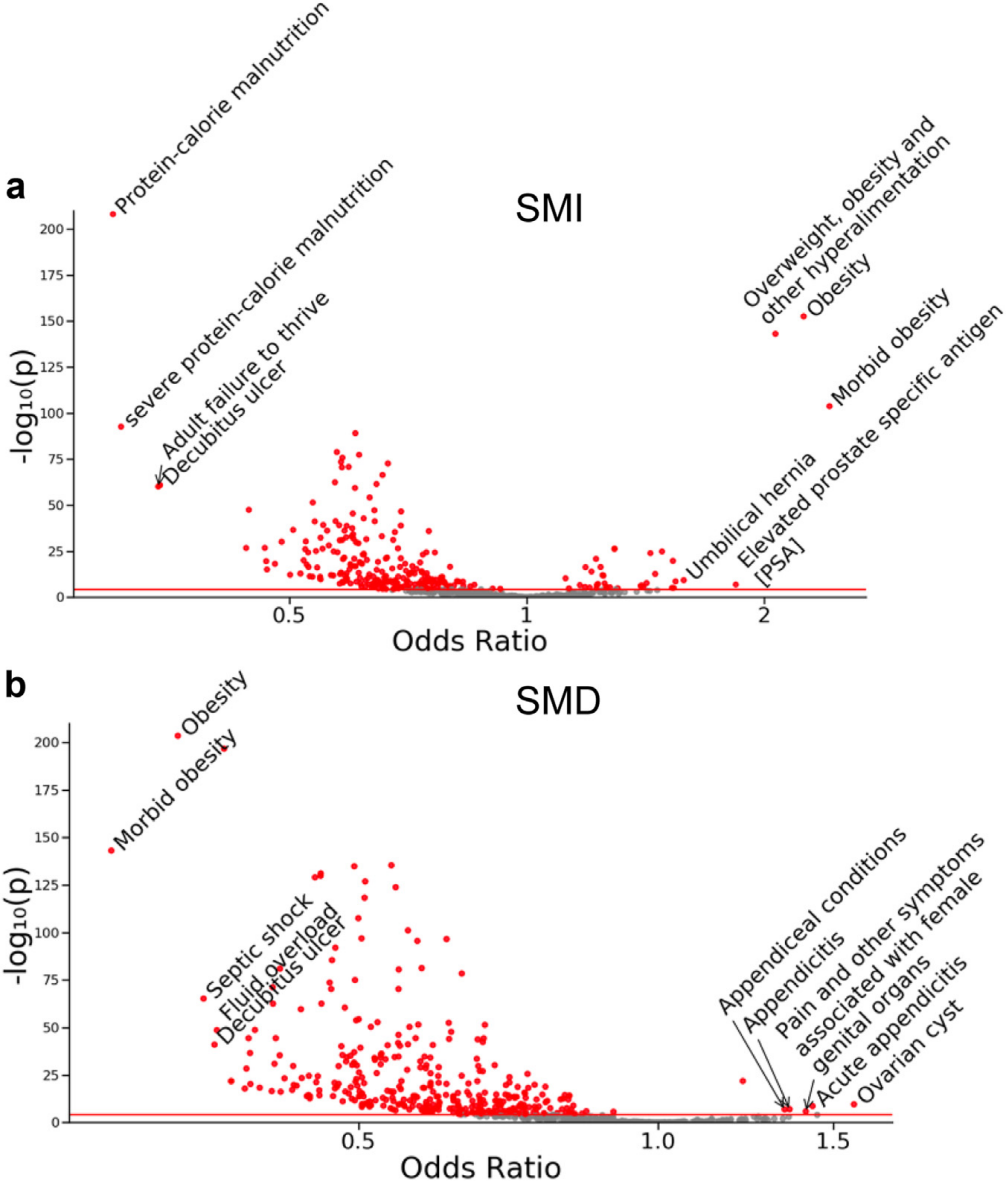
Volcano plot of PheWAS associations between SMI (a) and SMD (b) and ICD-derived medical phenotypes. Statistically significant associations are marked in red. The horizontal red bar corresponds to the Bonferroni significance threshold (0.05/1222). The 5 medical phenotypes with highest and lowest magnitude of association (|Log Odds Ratios|) are annotated. SMI, skeletal muscle index; SMD, skeletal muscle density; ICD, International Classification of Diseases.

Fig. 4 shows a Manhattan plot describing the significance of the association of CT-derived muscle metrics and medical phenotypes. The medical phenotype group with the highest proportion of statistically significant associations was hematopoietic: 75% (18/24) and 92% (22/24) for SMI and SMD, respectively. Fig. 5 shows an alternative visualization of the data using a Volcano plot, where both the effect size and statistical significance of each association are shown. Interactive versions of Figs. 4 and 5 are available at the following website: https://github.com/StanfordMIMI/muscle_phewas/blob/main/muscle_phewas.R. This multimedia supplementary material also supports interactive display of odds ratios for the associations between SMI and SMD and ICD-derived medical phenotypes.

Table 4 shows *previously unreported* associations between CT-derived muscle metrics and medical phenotypes, which to our knowledge have not been previously described in the literature. For higher SMI, this included decreased cardiac dysrhythmias (OR, 0.59 [0.55–0.64]; P < 0.0001 [PheWAS]), decreased hypopotassaemia (hypokalaemia) (OR, 0.59 [0.54–0.64]; P < 0.0001 [PheWAS]), decreased epilepsy (OR, 0.59 [0.50–0.70]; P < 0.0001 [PheWAS]), and increased elevated prostate-specific antigen (OR, 1.84 [1.47–2.31]; P < 0.0001 [PheWAS]). For higher SMD, this included decreased decubitus ulcers (OR, 0.36 [0.31–0.42]; P < 0.0001 [PheWAS]), osteomyelitis (OR, 0.43 [0.36–0.52]; P < 0.0001 [PheWAS]), chronic ulcers of skin (OR, 0.39 [0.35–0.45]; P < 0.0001 [PheWAS]), heart transplant/surgery (OR, 0.41 [0.33–0.50]; P < 0.0001 [PheWAS]) and circadian rhythm sleep disorders (OR, 0.39 [0.32–0.47]; P < 0.0001 [PheWAS]).

**Table 4:** Top 5 previously unreported associations between SMI (a) and SMD (b) and medical phenotypes.

a) Phenotype	Cases (n)	Controls (n)	OR (95% CI)	P value
Supraventricular premature beats	191	12,606	0.54 (0.46–0.66)	<0.0001
Hypopotassaemia	643	15,001	0.59 (0.54–0.64)	<0.0001
Other specified cardiac dysrhythmias	814	12,606	0.59 (0.55–0.64)	<0.0001
Epilepsy	136	16,355	0.59 (0.50–0.70)	<0.0001
Elevated prostate-specific antigen	108	6261	1.84 (1.47–2.31)	<0.0001
**b) Phenotype**	**Cases (n)**	**Controls (n)**	**OR (95% CI)**	**P value**
Decubitus ulcer	238	17,293	0.36 (0.31–0.42)	<0.0001
Circadian rhythm sleep disorder	118	15,923	0.39 (0.32–0.47)	<0.0001
Chronic ulcer of skin	346	17,293	0.39 (0.35–0.45)	<0.0001
Heart transplant/surgery	102	16,703	0.41 (0.33–0.50)	<0.0001
Osteomyelitis, periostitis, and other infections involving bone	155	17,063	0.43 (0.36–0.52)	<0.0001

Note.–Top refers to those with highest magnitude of association without prior evidence in the literature. SMI, skeletal muscle index; SMD, skeletal muscle density; OR, sex- and age-adjusted odds ratio; CI, confidence interval; n, number.

Table 5 shows *expected* associations between CT-derived muscle metrics and medical phenotypes that have previously been reported in the literature and are validated by our approach. For higher SMI, this included decreased protein-calorie malnutrition (OR, 0.30 [0.28–0.32]; P < 0.0001 [PheWAS]), decreased failure to thrive (OR, 0.34 [0.30–0.39]; P < 0.0001 [PheWAS]), decreased decubitus ulcers (OR, 0.34 [0.30–0.39]; P < 0.0001 [PheWAS]), and increased morbid obesity (OR, 2.42 [2.24–2.62]; P < 0.0001 [PheWAS]). For higher SMD, this included decreased morbid obesity (OR, 0.28 [0.26–0.31]; P < 0.0001 [PheWAS]), septic shock (OR, 0.35 [0.31–0.39]; P < 0.0001 [PheWAS]), and fluid overload (OR, 0.36 [0.31–0.41]; P < 0.0001 [PheWAS]).

**Table 5:** Top 5 validated associations between SMI (a) and SMD (b) and medical phenotypes.

a) Phenotype	Cases (n)	Controls (n)	OR (95% CI)	P value	Prior evidence
Protein-calorie malnutrition	1220	15,673	0.30 (0.28–0.32)	<0.0001	^22^
Severe protein-calorie malnutrition	352	15,673	0.31 (0.27–0.34)	<0.0001	^22^
Adult failure to thrive	244	15,673	0.34 (0.30–0.39)	<0.0001	^23^
Decubitus ulcer	238	17,300	0.34 (0.30–0.39)	<0.0001	^24^
Morbid obesity	655	16,054	2.42 (2.24–2.62)	<0.0001	^10^^,^^25^
**b) Phenotype**	**Cases (n)**	**Controls (n)**	**OR (95% CI)**	**P value**	**Prior evidence**
Morbid obesity	655	16,048	0.28 (0.26–0.31)	<0.0001	^26^
Obesity	1350	16,048	0.33 (0.31–0.35)	<0.0001	^26^
Septic shock	380	15,963	0.35 (0.31–0.39)	<0.0001	^27^
Fluid overload	275	14,995	0.36 (0.31–0.41)	<0.0001	^28^
Overweight/obesity	1591	16,048	0.36 (0.34–0.39)	<0.0001	^26^

Note.–Top refers to those with highest magnitude of association and validated refers to those with prior evidence in the literature. SMI, skeletal muscle index; SMD, skeletal muscle density; OR, sex- and age-adjusted odds ratio; CI, confidence interval; n, number.

Among the nonlinear terms, 29 SMI and 81 SMD were statistically significant at the Bonferroni threshold. In all such cases the model containing the nonlinear term was preferred. Table S2 summarizes for each of these associations whether they had been determined statistically significant in the linear only model, and whether in the nonlinear model the linear, square or both terms were found to be significant. Among the 29 nonlinear SMI models, 8 associations that were not significant in the linear only model were found to be significant in the nonlinear model. Twelve of the 81 SMD associations were not significant in the linear only model but were significant in the nonlinear model. The remaining associations had been previously identified as significant in the linear only analyses. Table S3 includes the nonlinear associations where the nonlinear model was preferred.

## Discussion

Our PheWAS study used abdominal CT scans of 17,646 patients acquired during routine clinical practice to reveal *previously unreported* associations between CT-derived biomarkers of myopenia and myosteatosis and EHR-derived medical phenotypes. In addition to providing body composition distributions for SMI and SMD in a large North American cohort, we validate prior findings that illustrate their variability across age and sex subgroups. Furthermore, we validated several known associations of SMI and SMD with medical phenotypes. The tools we provide to analyse and visualize these associations facilitate further hypothesis generation in studies of CT-derived metrics and their relation to medical phenotypes.

We identified previously unreported, medical phenotype associations for both SMI and SMD. For example, with increased SMI, we showed associations with decreased odds of supraventricular premature beats and other specified cardiac dysrhythmias. Dual-energy X-ray absorptiometry-derived metrics of myopenia linked with other specific types of cardiac dysrhythmia, such as premature ventricular contraction in individuals with normal body weight and atrial fibrillation among obese individuals,^29^ suggests associations between skeletal muscle quantity and myocardial conductivity. We showed these associations extend to other types of dysrhythmias and are also relevant in CT-based metrics. Increased SMI was also significantly associated with decreased epilepsy phenotype. Skeletal muscle is increasingly recognized as a metabolically active tissue that releases endocrine-like substances that affect other organs, including the brain.^30^ Examples of muscle–brain interactions previously described include muscle-secreted myokines and neurotrophic factors that can stimulate synaptic plasticity, promote neurogenesis, and improve cognition. People with epilepsy have been previously identified as being less physically active, with decreased aerobic endurance, muscle strength and physical flexibility.^31^ Our findings suggest people with epilepsy are also more likely to have decreased SMI, a surrogate of myopenia. The potential causality and direction of this association can be studied in future work. These examples illustrate the ability of PheWAS analyses to generate new evidence for associations between CT-derived metrics and EHR-derived medical phenotypes. Future studies will be needed to confirm the previously unreported associations of CT-derived muscle metrics with cardiac and brain disorders, and to identify the potential biologic causal mechanisms underlying the associations.

Our PheWAS analysis also validated medical phenotype associations with SMI and SMD that were previously reported in the literature. For example, we showed an association between protein-calorie malnutrition and decreased SMI, in accordance with prior studies showing malnutrition as one of the primary causes of myopenia.^22^ We also showed morbid obesity associated with increased SMI and inversely associated with SMD (OR [95% CI]: 2.42 [2.24–2.62] for SMI, 0.28 [0.26–0.31] for SMD). This is consistent with a previously-described increase in lean body mass among patient with obesity, with an accompanying decrease in SMD.^32^^,^^33^ These examples illustrate the power of PheWAS analysis to confirm known associations between CT-derived metrics and diseases.

There are several additional implications of our results. Our study shows the value of leveraging routine CT exams opportunistically at a population scale. In the future, such automated analysis of routine CT scans may improve patient care. Identification of CT biomarkers of myopenia and myosteatosis and their associations with diseases may provide value to many different types of clinicians. For example, urologists may be interested in CT screening, since our findings show that in males elevated prostate-specific antigen (PSA) is associated with increased SMI (OR [95% CI] 1.84 [1.47–2.31]). Increased testosterone levels may increase both SMI and PSA. Alternatively, rates of low serum testosterone and prostate cancer increase with age, as do rates of low muscle mass.^34^ Further, lower PSA levels have been found in individuals with higher body mass index, a relationship that is not necessarily explained by adiposity alone.^34^, ^35^, ^36^ Our study shows increased muscle mass with obesity, increasing the potential complexity of this association. Further studies may evaluate whether a different threshold for increased prostate-specific antigen should be used when screening patients to account for potential confounding effects of varying muscle composition. We encourage physicians and other researchers with other specific interests to explore further associations of SMI, SMD, and specific medical phenotypes in Tables S1 and S3 and our interactive Figs. 4 and 5 available on our website. Given our findings, different study designs such as case–control trials may be performed to assess the previously unreported PheWAS associations. Additionally, future basic science research may aim to establish potential causal mechanisms between new and validated associations, such as those identified by our study for myopenia with cardiac dysrhythmias and epilepsy.

Our study has several limitations. PheWAS analyses can identify many associations between imaging biomarkers and medical phenotypes, however, subsequent studies with alternative study designs should be carried out to study the nature of newly described associations, which may or may not be causal and may result from unobserved confounding. Additionally, such future studies may also clarify the role of adjustment variables as confounders or covariates for a specific association. Further, this was a retrospective analysis of data from a single institution. The associations between CT metrics and diagnoses may be biased in this cohort of patients undergoing abdominal CT examination during routine practice. However, there is pragmatic value in the high-throughput opportunistic analysis of already-acquired CT scans, and our cohort is representative of patients undergoing CT examinations in routine practice. Furthermore, although our cohort is large and contains individuals of diverse racial and ethnic backgrounds (Asian, Hispanic, and White), Black and Native American patients are underrepresented. In addition, we could not reliably identify individuals receiving gender-affirming therapy due to possible limitations in the documentation of sex vs gender in our EHR. Given that gender-affirming therapy may impact body composition,^37^ subsequent studies should evaluate the potential of such therapy on CT-derived muscle metrics and associated medical phenotypes. Furthermore, our methodology for identifying medical phenotypes relies on ICD codes, which may not precisely capture medical phenotypes since they are used generally for billing and quality improvement. We used this approach since it has been extensively validated in prior PheWAS work outside of radiology. Future studies may explore different, less-established medical phenotype definitions, such as deep phenotyping or laboratory exam-based phenotypes.^14^^,^^38^

In summary, our study illustrates the power of PheWAS analysis for broad radiology research in general and for myopenia and myosteatosis research in particular. We found evidence for previously unreported associations between CT-derived markers of myopenia and myosteatosis with medical phenotypes. In addition to facilitating hypothesis generation, this study paves the way for other imaging-based PheWAS studies exploring the associations of imaging biomarkers with medical phenotypes on a population scale.

## Contributors

Conceptualisation: JZC, AC, RB.

Data Curation and Verification: JZC, IG, LB, AC.

Formal Analysis: JZC, LL, TL, DR, MW, AC, RB.

Funding Acquisition: DR, AC, RB.

Writing–original draft: JZC.

Writing–review and editing: All authors.

All authors have approved the final version of the manuscript and agree to be accountable for all aspects of the work.

## Data sharing statement

The raw data are not publicly available due to privacy information embedded directly within the data. Data are available on request due to privacy or other restrictions. The data that support the findings of this study are available on request from the corresponding author (RDB).

## Declaration of interests

JMZC receives funding from Stanford Knight-Hennessy scholars and research support from GE HealthCare. LL receives funding from National Institutes of Health/National Institute on Aging grants P30 AG021332 and R21 AG070804. IG receives funding from Stanford Knight-Hennessy scholars, the Fannie & John Hertz Foundation, National Science Foundation Graduate Research Fellowship under Grant No. DGE-2146755, and the National GEM Consortium. LB receives funding from the Stanford Graduate Fund. DLR receives research support from Philips and National Institutes of Health grant U24CA226110. ASC receives research support from GE HealthCare, Philips, Stanford Precision Health and Integrated Diagnostics Center, Stanford Artificial Intelligence in Medicine and Imaging-Human centered Artificial Intelligence Partnership Grant, and National Institutes of Health grants R01 AR077604, R01 EB002524, R01 AR079431, HL167974, K24 AR062068, and P41 EB027060. RDB receives research support from GE HealthCare and is president elect of the Society of Academic Bone Radiologists.
