# Supplementary material for: Abdominal CT metrics in 17,646 patients reveal associations between myopenia, myosteatosis, and medical phenotypes: a phenome-wide association study
Source: eBioMedicine. 2024 Apr 17;103:105116. doi: 10.1016/j.ebiom.2024.105116 (PMC11031722; doi:10.1016/j.ebiom.2024.105116)
Supplement: Supplementary Material [file mmc1.docx]

Table S1: Associations between SMI and SMD with 611 medical phenotypes.

| **Phenotype** | **Description** | **Phenotype Group** | **Metric** | **P value** | **OR (95% CI)** | **Cases (n)** | **Controls (n)** |
| --- | --- | --- | --- | --- | --- | --- | --- |
| 260 | Protein-calorie malnutrition | endocrine/metabolic | SMI | < 0.0001 | 0.30 (0.28 - 0.32) | 1220 | 15673 |
| 278.1 | Obesity | endocrine/metabolic | SMD | < 0.0001 | 0.33 (0.31 - 0.35) | 1350 | 16048 |
| 278 | Overweight, obesity and other hyperalimentation | endocrine/metabolic | SMD | < 0.0001 | 0.37 (0.34 - 0.39) | 1591 | 16048 |
| 278.1 | Obesity | endocrine/metabolic | SMI | < 0.0001 | 2.25 (2.11 - 2.38) | 1350 | 16054 |
| 278.11 | Morbid obesity | endocrine/metabolic | SMD | < 0.0001 | 0.28 (0.26 - 0.31) | 655 | 16048 |
| 278 | Overweight, obesity and other hyperalimentation | endocrine/metabolic | SMI | < 0.0001 | 2.07 (1.96 - 2.19) | 1592 | 16054 |
| 427 | Cardiac dysrhythmias | circulatory system | SMD | < 0.0001 | 0.54 (0.51 - 0.57) | 3636 | 12600 |
| 426.7 | Abnormal electrocardiogram [ECG] [EKG] | circulatory system | SMD | < 0.0001 | 0.50 (0.47 - 0.52) | 2656 | 12600 |
| 994 | Sepsis and SIRS | injuries & poisonings | SMD | < 0.0001 | 0.46 (0.43 - 0.49) | 1676 | 15963 |
| 038 | Septicemia | infectious diseases | SMD | < 0.0001 | 0.46 (0.43 - 0.49) | 1686 | 15348 |
| 994.2 | Sepsis | injuries & poisonings | SMD | < 0.0001 | 0.45 (0.42 - 0.48) | 1588 | 15963 |
| 250 | Diabetes mellitus | endocrine/metabolic | SMD | < 0.0001 | 0.51 (0.48 - 0.54) | 2395 | 14747 |
| 426 | Cardiac conduction disorders | circulatory system | SMD | < 0.0001 | 0.55 (0.52 - 0.57) | 3578 | 12600 |
| 250.2 | Type 2 diabetes | endocrine/metabolic | SMD | < 0.0001 | 0.51 (0.48 - 0.54) | 2193 | 14747 |
| 585 | Renal failure | genitourinary | SMD | < 0.0001 | 0.50 (0.47 - 0.53) | 1829 | 14818 |
| 278.11 | Morbid obesity | endocrine/metabolic | SMI | < 0.0001 | 2.42 (2.24 - 2.62) | 655 | 16054 |
| 276 | Disorders of fluid, electrolyte, and acid-base balance | endocrine/metabolic | SMD | < 0.0001 | 0.56 (0.53 - 0.59) | 2544 | 14995 |
| 507 | Pleurisy; pleural effusion | respiratory | SMD | < 0.0001 | 0.50 (0.47 - 0.54) | 1602 | 15342 |
| 401 | Hypertension | circulatory system | SMD | < 0.0001 | 0.61 (0.59 - 0.64) | 4422 | 13217 |
| 514 | Abnormal findings examination of lungs | respiratory | SMD | < 0.0001 | 0.57 (0.54 - 0.60) | 2656 | 14743 |
| 260.2 | severe protein-calorie malnutrition | endocrine/metabolic | SMI | < 0.0001 | 0.31 (0.27 - 0.34) | 352 | 15673 |
| 427.7 | Tachycardia NOS | circulatory system | SMD | < 0.0001 | 0.47 (0.44 - 0.51) | 1237 | 12600 |
| 276 | Disorders of fluid, electrolyte, and acid-base balance | endocrine/metabolic | SMI | < 0.0001 | 0.61 (0.58 - 0.64) | 2545 | 15001 |
| 585.1 | Acute renal failure | genitourinary | SMD | < 0.0001 | 0.47 (0.44 - 0.51) | 1097 | 14818 |
| 285 | Other anemias | hematopoietic | SMD | < 0.0001 | 0.58 (0.55 - 0.61) | 2204 | 14700 |
| 509 | Respiratory failure, insufficiency, arrest | respiratory | SMD | < 0.0001 | 0.42 (0.38 - 0.46) | 712 | 15342 |
| 276.1 | Electrolyte imbalance | endocrine/metabolic | SMD | < 0.0001 | 0.55 (0.52 - 0.58) | 1749 | 14995 |
| 038 | Septicemia | infectious diseases | SMI | < 0.0001 | 0.57 (0.54 - 0.61) | 1686 | 15355 |
| 401.1 | Essential hypertension | circulatory system | SMD | < 0.0001 | 0.64 (0.61 - 0.67) | 4012 | 13217 |
| 285 | Other anemias | hematopoietic | SMI | < 0.0001 | 0.61 (0.58 - 0.65) | 2205 | 14706 |
| 994 | Sepsis and SIRS | injuries & poisonings | SMI | < 0.0001 | 0.58 (0.55 - 0.62) | 1676 | 15970 |
| 041 | Bacterial infection NOS | infectious diseases | SMD | < 0.0001 | 0.50 (0.46 - 0.53) | 1100 | 15348 |
| 427.2 | Atrial fibrillation and flutter | circulatory system | SMD | < 0.0001 | 0.47 (0.43 - 0.51) | 1234 | 12600 |
| 994.2 | Sepsis | injuries & poisonings | SMI | < 0.0001 | 0.58 (0.55 - 0.62) | 1588 | 15970 |
| 427 | Cardiac dysrhythmias | circulatory system | SMI | < 0.0001 | 0.67 (0.64 - 0.70) | 3636 | 12606 |
| 509.1 | Respiratory failure | respiratory | SMD | < 0.0001 | 0.41 (0.37 - 0.45) | 589 | 15342 |
| 276.1 | Electrolyte imbalance | endocrine/metabolic | SMI | < 0.0001 | 0.59 (0.56 - 0.63) | 1750 | 15001 |
| 507 | Pleurisy; pleural effusion | respiratory | SMI | < 0.0001 | 0.58 (0.55 - 0.62) | 1602 | 15349 |
| 427.21 | Atrial fibrillation | circulatory system | SMD | < 0.0001 | 0.47 (0.43 - 0.51) | 1180 | 12600 |
| 572 | Ascites (non malignant) | digestive | SMD | < 0.0001 | 0.55 (0.51 - 0.59) | 1477 | 13909 |
| 514 | Abnormal findings examination of lungs | respiratory | SMI | < 0.0001 | 0.66 (0.63 - 0.69) | 2656 | 14750 |
| 994.21 | Septic shock | injuries & poisonings | SMD | < 0.0001 | 0.35 (0.31 - 0.39) | 380 | 15963 |
| 508 | Pulmonary collapse; interstitial and compensatory emphysema | respiratory | SMD | < 0.0001 | 0.46 (0.42 - 0.50) | 683 | 15342 |
| 503 | Pulmonary congestion and hypostasis | respiratory | SMD | < 0.0001 | 0.41 (0.37 - 0.46) | 519 | 15342 |
| 427.7 | Tachycardia NOS | circulatory system | SMI | < 0.0001 | 0.57 (0.53 - 0.61) | 1237 | 12606 |
| 198 | Secondary malignant neoplasm | neoplasms | SMI | < 0.0001 | 0.65 (0.61 - 0.68) | 2160 | 14143 |
| 707.1 | Decubitus ulcer | dermatologic | SMI | < 0.0001 | 0.34 (0.30 - 0.39) | 238 | 17300 |
| 585.3 | Chronic renal failure [CKD] | genitourinary | SMD | < 0.0001 | 0.48 (0.44 - 0.53) | 828 | 14818 |
| 260.3 | Adult failure to thrive | endocrine/metabolic | SMI | < 0.0001 | 0.34 (0.30 - 0.39) | 244 | 15673 |
| 458.9 | Hypotension NOS | circulatory system | SMD | < 0.0001 | 0.44 (0.40 - 0.48) | 570 | 16641 |
| 195 | Cancer, suspected or other | neoplasms | SMI | < 0.0001 | 0.61 (0.57 - 0.64) | 1452 | 14143 |
| 428 | Congestive heart failure; nonhypertensive | circulatory system | SMD | < 0.0001 | 0.50 (0.46 - 0.55) | 820 | 16703 |
| 560 | Intestinal obstruction without mention of hernia | digestive | SMI | < 0.0001 | 0.63 (0.60 - 0.67) | 1698 | 11805 |
| 458 | Hypotension | circulatory system | SMD | < 0.0001 | 0.50 (0.46 - 0.54) | 741 | 16641 |
| 681 | Superficial cellulitis and abscess | dermatologic | SMD | < 0.0001 | 0.52 (0.48 - 0.57) | 823 | 16721 |
| 571 | Chronic liver disease and cirrhosis | digestive | SMD | < 0.0001 | 0.62 (0.58 - 0.66) | 1717 | 13909 |
| 452 | Other venous embolism and thrombosis | circulatory system | SMD | < 0.0001 | 0.57 (0.53 - 0.62) | 1178 | 15238 |
| 292.4 | Altered mental status | mental disorders | SMI | < 0.0001 | 0.54 (0.49 - 0.58) | 805 | 16104 |
| 512 | Other symptoms of respiratory system | respiratory | SMD | < 0.0001 | 0.67 (0.64 - 0.71) | 2608 | 15031 |
| 292.4 | Altered mental status | mental disorders | SMD | < 0.0001 | 0.51 (0.47 - 0.56) | 805 | 16099 |
| 292 | Neurological disorders | mental disorders | SMD | < 0.0001 | 0.56 (0.52 - 0.61) | 1117 | 16099 |
| 707 | Chronic ulcer of skin | dermatologic | SMD | < 0.0001 | 0.39 (0.35 - 0.45) | 346 | 17293 |
| 276.6 | Fluid overload | endocrine/metabolic | SMD | < 0.0001 | 0.36 (0.31 - 0.41) | 275 | 14995 |
| 512.7 | Shortness of breath | respiratory | SMD | < 0.0001 | 0.62 (0.58 - 0.66) | 1556 | 15031 |
| 707 | Chronic ulcer of skin | dermatologic | SMI | < 0.0001 | 0.44 (0.40 - 0.50) | 346 | 17300 |
| 572 | Ascites (non malignant) | digestive | SMI | < 0.0001 | 0.64 (0.60 - 0.68) | 1477 | 13915 |
| 426.7 | Abnormal electrocardiogram [ECG] [EKG] | circulatory system | SMI | < 0.0001 | 0.69 (0.66 - 0.73) | 2657 | 12606 |
| 782.3 | Edema | symptoms | SMD | < 0.0001 | 0.55 (0.51 - 0.60) | 877 | 16728 |
| 292 | Neurological disorders | mental disorders | SMI | < 0.0001 | 0.60 (0.56 - 0.65) | 1118 | 16104 |
| 276.4 | Acid-base balance disorder | endocrine/metabolic | SMD | < 0.0001 | 0.41 (0.37 - 0.47) | 354 | 14995 |
| 327.3 | Sleep apnea | neurological | SMD | < 0.0001 | 0.57 (0.53 - 0.62) | 977 | 15923 |
| 585.32 | End stage renal disease | genitourinary | SMD | < 0.0001 | 0.39 (0.34 - 0.44) | 293 | 14818 |
| 296 | Mood disorders | mental disorders | SMD | < 0.0001 | 0.67 (0.63 - 0.71) | 2152 | 14160 |
| 571.5 | Other chronic nonalcoholic liver disease | digestive | SMD | < 0.0001 | 0.62 (0.58 - 0.66) | 1386 | 13909 |
| 296.2 | Depression | mental disorders | SMD | < 0.0001 | 0.66 (0.62 - 0.70) | 1947 | 14160 |
| 041 | Bacterial infection NOS | infectious diseases | SMI | < 0.0001 | 0.62 (0.58 - 0.66) | 1100 | 15355 |
| 783 | Fever of unknown origin | symptoms | SMD | < 0.0001 | 0.67 (0.63 - 0.71) | 1943 | 15696 |
| 427.3 | Other specified cardiac dysrhythmias | circulatory system | SMD | < 0.0001 | 0.55 (0.50 - 0.60) | 814 | 12600 |
| 458 | Hypotension | circulatory system | SMI | < 0.0001 | 0.57 (0.53 - 0.62) | 741 | 16647 |
| 560.4 | Other intestinal obstruction | digestive | SMI | < 0.0001 | 0.64 (0.60 - 0.68) | 1300 | 11805 |
| 327.32 | Obstructive sleep apnea | neurological | SMD | < 0.0001 | 0.56 (0.52 - 0.61) | 834 | 15923 |
| 285.2 | Anemia of chronic disease | hematopoietic | SMI | < 0.0001 | 0.54 (0.49 - 0.59) | 581 | 14706 |
| 707.1 | Decubitus ulcer | dermatologic | SMD | < 0.0001 | 0.36 (0.31 - 0.42) | 238 | 17293 |
| 567 | Peritonitis and retroperitoneal infections | digestive | SMD | < 0.0001 | 0.59 (0.55 - 0.64) | 1039 | 14829 |
| 275 | Disorders of mineral metabolism | endocrine/metabolic | SMD | < 0.0001 | 0.54 (0.49 - 0.59) | 671 | 16968 |
| 415 | Pulmonary heart disease | circulatory system | SMD | < 0.0001 | 0.55 (0.50 - 0.60) | 770 | 16487 |
| 416 | Cardiomegaly | circulatory system | SMD | < 0.0001 | 0.48 (0.43 - 0.54) | 485 | 16487 |
| 038.3 | Bacteremia | infectious diseases | SMD | < 0.0001 | 0.50 (0.45 - 0.56) | 557 | 15348 |
| 509.1 | Respiratory failure | respiratory | SMI | < 0.0001 | 0.55 (0.51 - 0.60) | 589 | 15349 |
| 509 | Respiratory failure, insufficiency, arrest | respiratory | SMI | < 0.0001 | 0.58 (0.53 - 0.63) | 712 | 15349 |
| 798 | Malaise and fatigue | symptoms | SMI | < 0.0001 | 0.69 (0.66 - 0.73) | 1832 | 15814 |
| 427.3 | Other specified cardiac dysrhythmias | circulatory system | SMI | < 0.0001 | 0.59 (0.55 - 0.64) | 814 | 12606 |
| 198.4 | Secondary malignant neoplasm of liver | neoplasms | SMI | < 0.0001 | 0.60 (0.56 - 0.65) | 789 | 14143 |
| 250.3 | Insulin pump user | endocrine/metabolic | SMD | < 0.0001 | 0.50 (0.44 - 0.55) | 487 | 14747 |
| 585.31 | Renal dialysis | genitourinary | SMD | < 0.0001 | 0.39 (0.34 - 0.45) | 238 | 14818 |
| 994.21 | Septic shock | injuries & poisonings | SMI | < 0.0001 | 0.51 (0.46 - 0.56) | 380 | 15970 |
| 452.2 | Deep vein thrombosis [DVT] | circulatory system | SMD | < 0.0001 | 0.53 (0.48 - 0.58) | 575 | 15238 |
| 458.9 | Hypotension NOS | circulatory system | SMI | < 0.0001 | 0.56 (0.51 - 0.61) | 570 | 16647 |
| 275 | Disorders of mineral metabolism | endocrine/metabolic | SMI | < 0.0001 | 0.59 (0.54 - 0.64) | 672 | 16974 |
| 426 | Cardiac conduction disorders | circulatory system | SMI | < 0.0001 | 0.75 (0.72 - 0.79) | 3579 | 12606 |
| 285.2 | Anemia of chronic disease | hematopoietic | SMD | < 0.0001 | 0.53 (0.48 - 0.58) | 581 | 14700 |
| 428.1 | Congestive heart failure (CHF) NOS | circulatory system | SMD | < 0.0001 | 0.48 (0.43 - 0.54) | 435 | 16703 |
| 276.41 | Acidosis | endocrine/metabolic | SMD | < 0.0001 | 0.42 (0.36 - 0.48) | 282 | 14995 |
| 569 | Other disorders of intestine | digestive | SMI | < 0.0001 | 0.68 (0.64 - 0.72) | 1367 | 14836 |
| 426.3 | Bundle branch block | circulatory system | SMD | < 0.0001 | 0.49 (0.44 - 0.55) | 539 | 12600 |
| 287 | Purpura and other hemorrhagic conditions | hematopoietic | SMD | < 0.0001 | 0.55 (0.50 - 0.60) | 617 | 16143 |
| 480 | Pneumonia | respiratory | SMD | < 0.0001 | 0.58 (0.53 - 0.63) | 795 | 16737 |
| 287.3 | Thrombocytopenia | hematopoietic | SMD | < 0.0001 | 0.54 (0.49 - 0.60) | 592 | 16143 |
| 401.2 | Hypertensive heart and/or renal disease | circulatory system | SMD | < 0.0001 | 0.49 (0.43 - 0.55) | 465 | 13217 |
| 276.5 | Hypovolemia | endocrine/metabolic | SMI | < 0.0001 | 0.60 (0.56 - 0.65) | 742 | 15001 |
| 288 | Diseases of white blood cells | hematopoietic | SMD | < 0.0001 | 0.59 (0.54 - 0.64) | 822 | 15441 |
| 285.1 | Acute posthemorrhagic anemia | hematopoietic | SMD | < 0.0001 | 0.53 (0.48 - 0.58) | 547 | 14700 |
| 279 | Disorders involving the immune mechanism | endocrine/metabolic | SMD | < 0.0001 | 0.52 (0.47 - 0.58) | 474 | 17077 |
| 569 | Other disorders of intestine | digestive | SMD | < 0.0001 | 0.66 (0.61 - 0.70) | 1367 | 14829 |
| 317 | Alcohol-related disorders | mental disorders | SMD | < 0.0001 | 0.53 (0.48 - 0.59) | 513 | 15498 |
| 585.1 | Acute renal failure | genitourinary | SMI | < 0.0001 | 0.66 (0.61 - 0.70) | 1097 | 14825 |
| 555 | Inflammatory bowel disease and other gastroenteritis and colitis | digestive | SMI | < 0.0001 | 0.60 (0.55 - 0.65) | 650 | 11805 |
| 317.1 | Alcoholism | mental disorders | SMD | < 0.0001 | 0.52 (0.47 - 0.58) | 462 | 15498 |
| 276.14 | Hypopotassemia | endocrine/metabolic | SMI | < 0.0001 | 0.59 (0.54 - 0.64) | 643 | 15001 |
| 198.6 | Secondary malignancy of bone | neoplasms | SMI | < 0.0001 | 0.55 (0.50 - 0.60) | 446 | 14143 |
| 276.12 | Hyposmolality and/or hyponatremia | endocrine/metabolic | SMD | < 0.0001 | 0.58 (0.53 - 0.63) | 765 | 14995 |
| 427.6 | Premature beats | circulatory system | SMD | < 0.0001 | 0.49 (0.43 - 0.55) | 463 | 12600 |
| 276.12 | Hyposmolality and/or hyponatremia | endocrine/metabolic | SMI | < 0.0001 | 0.60 (0.55 - 0.66) | 765 | 15001 |
| 428.3 | Heart failure with reduced EF [Systolic or combined heart failure] | circulatory system | SMD | < 0.0001 | 0.49 (0.44 - 0.56) | 414 | 16703 |
| 480 | Pneumonia | respiratory | SMI | < 0.0001 | 0.63 (0.58 - 0.68) | 795 | 16744 |
| 284 | Aplastic anemia | hematopoietic | SMD | < 0.0001 | 0.50 (0.44 - 0.56) | 401 | 14700 |
| 452.2 | Deep vein thrombosis [DVT] | circulatory system | SMI | < 0.0001 | 0.58 (0.53 - 0.64) | 575 | 15244 |
| 452 | Other venous embolism and thrombosis | circulatory system | SMI | < 0.0001 | 0.68 (0.63 - 0.72) | 1179 | 15244 |
| 038.3 | Bacteremia | infectious diseases | SMI | < 0.0001 | 0.58 (0.53 - 0.64) | 557 | 15355 |
| 290.2 | Delirium due to conditions classified elsewhere | mental disorders | SMD | < 0.0001 | 0.41 (0.36 - 0.48) | 240 | 16099 |
| 539 | Bariatric surgery | digestive | SMD | < 0.0001 | 0.53 (0.47 - 0.59) | 478 | 17161 |
| 291 | Other specified nonpsychotic and/or transient mental disorders | mental disorders | SMD | < 0.0001 | 0.49 (0.44 - 0.56) | 380 | 16099 |
| 276.4 | Acid-base balance disorder | endocrine/metabolic | SMI | < 0.0001 | 0.52 (0.47 - 0.59) | 354 | 15001 |
| 512.9 | Other dyspnea | respiratory | SMD | < 0.0001 | 0.59 (0.53 - 0.64) | 704 | 15031 |
| 318 | Tobacco use disorder | mental disorders | SMD | < 0.0001 | 0.69 (0.64 - 0.73) | 1554 | 15498 |
| 260.6 | Anorexia | endocrine/metabolic | SMI | < 0.0001 | 0.49 (0.43 - 0.55) | 302 | 15673 |
| 284.1 | Pancytopenia | hematopoietic | SMD | < 0.0001 | 0.50 (0.44 - 0.56) | 391 | 14700 |
| 198.3 | Secondary malignant neoplasm of digestive systems | neoplasms | SMI | < 0.0001 | 0.61 (0.56 - 0.66) | 653 | 14143 |
| 260 | Protein-calorie malnutrition | endocrine/metabolic | SMD | < 0.0001 | 0.66 (0.61 - 0.71) | 1220 | 15668 |
| 317.11 | Alcoholic liver damage | mental disorders | SMD | < 0.0001 | 0.43 (0.37 - 0.50) | 225 | 15498 |
| 291.8 | Alteration of consciousness | mental disorders | SMD | < 0.0001 | 0.48 (0.42 - 0.54) | 340 | 16099 |
| 288.1 | Decreased white blood cell count | hematopoietic | SMI | < 0.0001 | 0.61 (0.56 - 0.67) | 613 | 15447 |
| 288.2 | Elevated white blood cell count | hematopoietic | SMD | < 0.0001 | 0.60 (0.55 - 0.66) | 752 | 15441 |
| 288.1 | Decreased white blood cell count | hematopoietic | SMD | < 0.0001 | 0.58 (0.53 - 0.64) | 613 | 15441 |
| 571.8 | Liver abscess and sequelae of chronic liver disease | digestive | SMD | < 0.0001 | 0.58 (0.53 - 0.64) | 661 | 13909 |
| 285.21 | Anemia in chronic kidney disease | hematopoietic | SMD | < 0.0001 | 0.39 (0.33 - 0.46) | 178 | 14700 |
| 198.2 | Secondary malignancy of respiratory organs | neoplasms | SMI | < 0.0001 | 0.56 (0.51 - 0.62) | 408 | 14143 |
| 288.11 | Neutropenia | hematopoietic | SMD | < 0.0001 | 0.56 (0.51 - 0.62) | 515 | 15441 |
| 411 | Ischemic Heart Disease | circulatory system | SMD | < 0.0001 | 0.66 (0.61 - 0.71) | 1325 | 16264 |
| 591 | Urinary tract infection | genitourinary | SMD | < 0.0001 | 0.67 (0.62 - 0.72) | 1186 | 15562 |
| 198.5 | Secondary malignancy of brain/spine | neoplasms | SMI | < 0.0001 | 0.47 (0.41 - 0.54) | 180 | 14143 |
| 501 | Pneumonitis due to inhalation of food or vomitus | respiratory | SMI | < 0.0001 | 0.44 (0.38 - 0.51) | 192 | 15349 |
| 567 | Peritonitis and retroperitoneal infections | digestive | SMI | < 0.0001 | 0.69 (0.64 - 0.74) | 1039 | 14836 |
| 272 | Disorders of lipoid metabolism | endocrine/metabolic | SMI | < 0.0001 | 1.29 (1.23 - 1.35) | 2884 | 14762 |
| 291 | Other specified nonpsychotic and/or transient mental disorders | mental disorders | SMI | < 0.0001 | 0.55 (0.49 - 0.61) | 381 | 16104 |
| 425 | Cardiomyopathy | circulatory system | SMD | < 0.0001 | 0.50 (0.44 - 0.57) | 332 | 17102 |
| 272.1 | Hyperlipidemia | endocrine/metabolic | SMI | < 0.0001 | 1.29 (1.23 - 1.35) | 2873 | 14762 |
| 559 | Ileostomy status | digestive | SMI | < 0.0001 | 0.52 (0.47 - 0.59) | 267 | 11805 |
| 560.1 | Paralytic ileus | digestive | SMD | < 0.0001 | 0.52 (0.46 - 0.59) | 371 | 11799 |
| 276.14 | Hypopotassemia | endocrine/metabolic | SMD | < 0.0001 | 0.59 (0.54 - 0.65) | 642 | 14995 |
| 338 | Pain | neurological | SMD | < 0.0001 | 0.74 (0.70 - 0.78) | 2136 | 15503 |
| 291.8 | Alteration of consciousness | mental disorders | SMI | < 0.0001 | 0.54 (0.48 - 0.61) | 340 | 16104 |
| 304 | Adjustment reaction | mental disorders | SMI | < 0.0001 | 0.57 (0.51 - 0.63) | 367 | 14166 |
| 327.32 | Obstructive sleep apnea | neurological | SMI | < 0.0001 | 1.48 (1.38 - 1.60) | 834 | 15928 |
| 296.22 | Major depressive disorder | mental disorders | SMD | < 0.0001 | 0.63 (0.58 - 0.69) | 805 | 14160 |
| 426.31 | Right bundle branch block | circulatory system | SMD | < 0.0001 | 0.45 (0.38 - 0.52) | 265 | 12600 |
| 286.2 | Encounter for long-term (current) use of anticoagulants | hematopoietic | SMD | < 0.0001 | 0.60 (0.55 - 0.66) | 635 | 16143 |
| 290 | Delirium dementia and amnestic and other cognitive disorders | mental disorders | SMI | < 0.0001 | 0.58 (0.52 - 0.64) | 590 | 16104 |
| 426.8 | Other cardiac conduction disorders | circulatory system | SMD | < 0.0001 | 0.46 (0.39 - 0.53) | 236 | 12600 |
| 585 | Renal failure | genitourinary | SMI | < 0.0001 | 0.75 (0.71 - 0.79) | 1829 | 14825 |
| 276.41 | Acidosis | endocrine/metabolic | SMI | < 0.0001 | 0.53 (0.47 - 0.60) | 282 | 15001 |
| 783 | Fever of unknown origin | symptoms | SMI | < 0.0001 | 0.76 (0.73 - 0.80) | 1943 | 15703 |
| 327.3 | Sleep apnea | neurological | SMI | < 0.0001 | 1.44 (1.34 - 1.54) | 977 | 15928 |
| 300 | Anxiety disorders | mental disorders | SMD | < 0.0001 | 0.71 (0.66 - 0.76) | 1502 | 14160 |
| 681.7 | Cellulitis and abscess of trunk | dermatologic | SMD | < 0.0001 | 0.50 (0.44 - 0.58) | 298 | 16721 |
| 288.11 | Neutropenia | hematopoietic | SMI | < 0.0001 | 0.62 (0.57 - 0.68) | 515 | 15447 |
| 427.22 | Atrial flutter | circulatory system | SMD | < 0.0001 | 0.44 (0.37 - 0.51) | 235 | 12600 |
| 041.2 | Streptococcus infection | infectious diseases | SMD | < 0.0001 | 0.42 (0.36 - 0.50) | 189 | 15348 |
| 279 | Disorders involving the immune mechanism | endocrine/metabolic | SMI | < 0.0001 | 0.62 (0.56 - 0.68) | 474 | 17084 |
| 197 | Chemotherapy | neoplasms | SMI | < 0.0001 | 0.74 (0.70 - 0.78) | 1450 | 14143 |
| 285.1 | Acute posthemorrhagic anemia | hematopoietic | SMI | < 0.0001 | 0.62 (0.57 - 0.68) | 547 | 14706 |
| 425.1 | Primary/intrinsic cardiomyopathies | circulatory system | SMD | < 0.0001 | 0.50 (0.43 - 0.57) | 278 | 17102 |
| 785 | Abdominal pain | symptoms | SMD | < 0.0001 | 1.22 (1.17 - 1.27) | 11046 | 6593 |
| 345 | Epilepsy, recurrent seizures, convulsions | neurological | SMD | < 0.0001 | 0.55 (0.49 - 0.62) | 379 | 16348 |
| 255.2 | Adrenal hypofunction | endocrine/metabolic | SMD | < 0.0001 | 0.37 (0.31 - 0.45) | 118 | 16932 |
| 255.21 | Glucocorticoid deficiency | endocrine/metabolic | SMD | < 0.0001 | 0.37 (0.31 - 0.45) | 118 | 16932 |
| 411.4 | Coronary atherosclerosis | circulatory system | SMD | < 0.0001 | 0.66 (0.61 - 0.72) | 1024 | 16264 |
| 195.1 | Malignant neoplasm, other | neoplasms | SMI | < 0.0001 | 0.67 (0.61 - 0.72) | 707 | 14143 |
| 560.1 | Paralytic ileus | digestive | SMI | < 0.0001 | 0.59 (0.53 - 0.65) | 371 | 11805 |
| 426.9 | Cardiac pacemaker/device in situ | circulatory system | SMD | < 0.0001 | 0.50 (0.43 - 0.57) | 313 | 12600 |
| 081 | Infection/inflammation of internal prosthetic device; implant; and graft | infectious diseases | SMD | < 0.0001 | 0.45 (0.39 - 0.53) | 181 | 17208 |
| 288 | Diseases of white blood cells | hematopoietic | SMI | < 0.0001 | 0.69 (0.64 - 0.75) | 822 | 15447 |
| 550 | Abdominal hernia | digestive | SMD | < 0.0001 | 0.71 (0.66 - 0.76) | 1261 | 16378 |
| 508 | Pulmonary collapse; interstitial and compensatory emphysema | respiratory | SMI | < 0.0001 | 0.66 (0.61 - 0.72) | 683 | 15349 |
| 290 | Delirium dementia and amnestic and other cognitive disorders | mental disorders | SMD | < 0.0001 | 0.59 (0.53 - 0.66) | 590 | 16099 |
| 041.1 | Staphylococcus infections | infectious diseases | SMD | < 0.0001 | 0.47 (0.41 - 0.55) | 227 | 15348 |
| 571.51 | Cirrhosis of liver without mention of alcohol | digestive | SMD | < 0.0001 | 0.60 (0.54 - 0.67) | 534 | 13909 |
| 284 | Aplastic anemia | hematopoietic | SMI | < 0.0001 | 0.60 (0.55 - 0.67) | 401 | 14706 |
| 401.1 | Essential hypertension | circulatory system | SMI | < 0.0001 | 1.22 (1.17 - 1.28) | 4013 | 13223 |
| 555.2 | Ulcerative colitis | digestive | SMI | < 0.0001 | 0.58 (0.52 - 0.65) | 321 | 11805 |
| 401.22 | Hypertensive chronic kidney disease | circulatory system | SMD | < 0.0001 | 0.51 (0.44 - 0.58) | 290 | 13217 |
| 276.5 | Hypovolemia | endocrine/metabolic | SMD | < 0.0001 | 0.65 (0.59 - 0.71) | 742 | 14995 |
| 275.5 | Disorders of calcium/phosphorus metabolism | endocrine/metabolic | SMD | < 0.0001 | 0.48 (0.41 - 0.56) | 213 | 16968 |
| 568 | Other disorders of peritoneum | digestive | SMD | < 0.0001 | 0.61 (0.55 - 0.68) | 548 | 14829 |
| 503 | Pulmonary congestion and hypostasis | respiratory | SMI | < 0.0001 | 0.63 (0.57 - 0.69) | 519 | 15349 |
| 327.6 | Circadian rhythm sleep disorder | neurological | SMD | < 0.0001 | 0.39 (0.32 - 0.47) | 118 | 15923 |
| 290.2 | Delirium due to conditions classified elsewhere | mental disorders | SMI | < 0.0001 | 0.52 (0.46 - 0.60) | 240 | 16104 |
| 204 | Leukemia | neoplasms | SMD | < 0.0001 | 0.59 (0.53 - 0.66) | 440 | 16677 |
| 798 | Malaise and fatigue | symptoms | SMD | < 0.0001 | 0.75 (0.71 - 0.80) | 1832 | 15807 |
| 539 | Bariatric surgery | digestive | SMI | < 0.0001 | 1.53 (1.40 - 1.68) | 478 | 17168 |
| 710 | Osteomyelitis, periostitis, and other infections involving bone | musculoskeletal | SMD | < 0.0001 | 0.43 (0.36 - 0.52) | 155 | 17063 |
| 338.2 | Chronic pain | neurological | SMD | < 0.0001 | 0.70 (0.65 - 0.76) | 1088 | 15503 |
| 480.1 | Bacterial pneumonia | respiratory | SMI | < 0.0001 | 0.47 (0.40 - 0.55) | 126 | 16744 |
| 710.1 | Osteomyelitis | musculoskeletal | SMD | < 0.0001 | 0.43 (0.36 - 0.52) | 154 | 17063 |
| 860 | Bone marrow or stem cell transplant | neoplasms | SMD | < 0.0001 | 0.50 (0.43 - 0.58) | 216 | 17423 |
| 990 | Effects radiation NOS | injuries & poisonings | SMI | < 0.0001 | 0.65 (0.59 - 0.71) | 547 | 15576 |
| 284.1 | Pancytopenia | hematopoietic | SMI | < 0.0001 | 0.61 (0.55 - 0.68) | 391 | 14706 |
| 550.5 | Ventral hernia | digestive | SMD | < 0.0001 | 0.59 (0.53 - 0.66) | 431 | 16378 |
| 276.13 | Hyperpotassemia | endocrine/metabolic | SMD | < 0.0001 | 0.48 (0.41 - 0.56) | 202 | 14995 |
| 575 | Other biliary tract disease | digestive | SMI | < 0.0001 | 0.73 (0.69 - 0.78) | 1119 | 15847 |
| 348 | Other conditions of brain | neurological | SMD | < 0.0001 | 0.55 (0.48 - 0.63) | 328 | 16348 |
| 198 | Secondary malignant neoplasm | neoplasms | SMD | < 0.0001 | 0.77 (0.73 - 0.82) | 2160 | 14136 |
| 300.1 | Anxiety disorder | mental disorders | SMD | < 0.0001 | 0.72 (0.67 - 0.78) | 1281 | 14160 |
| 415.2 | Chronic pulmonary heart disease | circulatory system | SMD | < 0.0001 | 0.49 (0.42 - 0.57) | 227 | 16487 |
| 575.2 | Obstruction of bile duct | digestive | SMI | < 0.0001 | 0.62 (0.56 - 0.69) | 412 | 15847 |
| 509.8 | Dependence on respirator [Ventilator] or supplemental oxygen | respiratory | SMD | < 0.0001 | 0.40 (0.33 - 0.49) | 119 | 15342 |
| 415.11 | Pulmonary embolism and infarction, acute | circulatory system | SMD | < 0.0001 | 0.58 (0.52 - 0.65) | 392 | 16487 |
| 195 | Cancer, suspected or other | neoplasms | SMD | < 0.0001 | 0.74 (0.69 - 0.79) | 1452 | 14136 |
| 415.1 | Acute pulmonary heart disease | circulatory system | SMD | < 0.0001 | 0.58 (0.52 - 0.66) | 394 | 16487 |
| 288.2 | Elevated white blood cell count | hematopoietic | SMI | < 0.0001 | 0.70 (0.65 - 0.76) | 752 | 15447 |
| 555.1 | Regional enteritis | digestive | SMI | < 0.0001 | 0.62 (0.56 - 0.69) | 351 | 11805 |
| 536.7 | Complications of gastrostomy, colostomy and enterostomy | digestive | SMI | < 0.0001 | 0.48 (0.41 - 0.57) | 132 | 16078 |
| 573.5 | Jaundice (not of newborn) | digestive | SMI | < 0.0001 | 0.60 (0.53 - 0.67) | 331 | 13915 |
| 348.8 | Encephalopathy, not elsewhere classified | neurological | SMD | < 0.0001 | 0.47 (0.40 - 0.56) | 194 | 16348 |
| 276.11 | Hyperosmolality and/or hypernatremia | endocrine/metabolic | SMD | < 0.0001 | 0.38 (0.31 - 0.48) | 110 | 14995 |
| 041.2 | Streptococcus infection | infectious diseases | SMI | < 0.0001 | 0.53 (0.46 - 0.61) | 189 | 15355 |
| 079 | Viral infection | infectious diseases | SMD | < 0.0001 | 0.55 (0.48 - 0.63) | 283 | 16466 |
| 348 | Other conditions of brain | neurological | SMI | < 0.0001 | 0.60 (0.53 - 0.67) | 328 | 16355 |
| 681.5 | Cellulitis and abscess of leg, except foot | dermatologic | SMD | < 0.0001 | 0.43 (0.35 - 0.52) | 131 | 16721 |
| 280 | Iron deficiency anemias | hematopoietic | SMI | < 0.0001 | 0.72 (0.66 - 0.77) | 822 | 14706 |
| 427.61 | Supraventricular premature beats | circulatory system | SMD | < 0.0001 | 0.46 (0.38 - 0.55) | 191 | 12600 |
| 008.52 | Intestinal infection due to C. difficile | infectious diseases | SMI | < 0.0001 | 0.60 (0.54 - 0.68) | 314 | 17040 |
| 427.6 | Premature beats | circulatory system | SMI | < 0.0001 | 0.61 (0.54 - 0.68) | 463 | 12606 |
| 275.3 | Disorders of magnesium metabolism | endocrine/metabolic | SMD | < 0.0001 | 0.53 (0.46 - 0.62) | 252 | 16968 |
| 561 | Symptoms involving digestive system | digestive | SMI | < 0.0001 | 0.80 (0.76 - 0.84) | 1959 | 11805 |
| 429.1 | Heart transplant/surgery | circulatory system | SMD | < 0.0001 | 0.41 (0.33 - 0.50) | 102 | 16703 |
| 008 | Intestinal infection | infectious diseases | SMI | < 0.0001 | 0.69 (0.63 - 0.75) | 606 | 17040 |
| 348.8 | Encephalopathy, not elsewhere classified | neurological | SMI | < 0.0001 | 0.53 (0.46 - 0.61) | 194 | 16355 |
| 117 | Mycoses | infectious diseases | SMD | < 0.0001 | 0.50 (0.43 - 0.59) | 189 | 16933 |
| 250.2 | Type 2 diabetes | endocrine/metabolic | SMI | < 0.0001 | 1.24 (1.18 - 1.31) | 2193 | 14753 |
| 401 | Hypertension | circulatory system | SMI | < 0.0001 | 1.19 (1.14 - 1.24) | 4423 | 13223 |
| 427.1 | Paroxysmal tachycardia, unspecified | circulatory system | SMD | < 0.0001 | 0.54 (0.47 - 0.62) | 278 | 12600 |
| 250.22 | Type 2 diabetes with renal manifestations | endocrine/metabolic | SMD | < 0.0001 | 0.49 (0.41 - 0.58) | 195 | 14747 |
| 304 | Adjustment reaction | mental disorders | SMD | < 0.0001 | 0.59 (0.53 - 0.67) | 367 | 14160 |
| 275.53 | Disorders of phosphorus metabolism | endocrine/metabolic | SMD | < 0.0001 | 0.42 (0.34 - 0.51) | 110 | 16968 |
| 285.22 | Anemia in neoplastic disease | hematopoietic | SMI | < 0.0001 | 0.54 (0.47 - 0.63) | 198 | 14706 |
| 585.33 | Chronic Kidney Disease, Stage III | genitourinary | SMD | < 0.0001 | 0.54 (0.47 - 0.63) | 300 | 14818 |
| 578.9 | Hemorrhage of gastrointestinal tract | digestive | SMD | < 0.0001 | 0.64 (0.58 - 0.71) | 538 | 15478 |
| 457 | Encounter for long-term (current) use of anticoagulants, antithrombotics, aspirin | circulatory system | SMD | < 0.0001 | 0.67 (0.61 - 0.74) | 706 | 15238 |
| 338.1 | Acute pain | neurological | SMD | < 0.0001 | 0.73 (0.68 - 0.79) | 1142 | 15503 |
| 578.9 | Hemorrhage of gastrointestinal tract | digestive | SMI | < 0.0001 | 0.68 (0.62 - 0.74) | 538 | 15485 |
| 977 | Personal history of allergy to medicinal agents | injuries & poisonings | SMD | < 0.0001 | 0.63 (0.56 - 0.70) | 460 | 15985 |
| 427.2 | Atrial fibrillation and flutter | circulatory system | SMI | < 0.0001 | 0.72 (0.67 - 0.78) | 1234 | 12606 |
| 038.1 | Gram negative septicemia | infectious diseases | SMD | < 0.0001 | 0.50 (0.43 - 0.60) | 204 | 15348 |
| 204.1 | Lymphoid leukemia | neoplasms | SMD | < 0.0001 | 0.49 (0.41 - 0.58) | 159 | 16677 |
| 575 | Other biliary tract disease | digestive | SMD | < 0.0001 | 0.74 (0.68 - 0.79) | 1119 | 15841 |
| 506 | Empyema and pneumothorax | respiratory | SMI | < 0.0001 | 0.60 (0.53 - 0.68) | 267 | 15349 |
| 737 | Curvature of spine | musculoskeletal | SMI | < 0.0001 | 0.47 (0.39 - 0.57) | 109 | 17330 |
| 008.5 | Bacterial enteritis | infectious diseases | SMI | < 0.0001 | 0.65 (0.58 - 0.72) | 373 | 17040 |
| 960 | Poisoning by antibiotics | injuries & poisonings | SMD | < 0.0001 | 0.69 (0.63 - 0.75) | 697 | 15985 |
| 280 | Iron deficiency anemias | hematopoietic | SMD | < 0.0001 | 0.70 (0.64 - 0.77) | 822 | 14700 |
| 197 | Chemotherapy | neoplasms | SMD | < 0.0001 | 0.77 (0.72 - 0.82) | 1450 | 14136 |
| 112 | Candidiasis | infectious diseases | SMD | < 0.0001 | 0.61 (0.54 - 0.69) | 370 | 16933 |
| 286 | Coagulation defects | hematopoietic | SMD | < 0.0001 | 0.65 (0.58 - 0.72) | 476 | 16143 |
| 530 | Diseases of esophagus | digestive | SMD | < 0.0001 | 0.81 (0.76 - 0.85) | 2393 | 14896 |
| 710.19 | Unspecified osteomyelitis | musculoskeletal | SMD | < 0.0001 | 0.45 (0.36 - 0.55) | 122 | 17063 |
| 287.3 | Thrombocytopenia | hematopoietic | SMI | < 0.0001 | 0.70 (0.64 - 0.77) | 592 | 16149 |
| 994.1 | Systemic inflammatory response syndrome (SIRS) | injuries & poisonings | SMD | < 0.0001 | 0.46 (0.38 - 0.56) | 135 | 15963 |
| 198.4 | Secondary malignant neoplasm of liver | neoplasms | SMD | < 0.0001 | 0.71 (0.65 - 0.77) | 789 | 14136 |
| 272.1 | Hyperlipidemia | endocrine/metabolic | SMD | < 0.0001 | 0.81 (0.77 - 0.86) | 2873 | 14755 |
| 457.3 | Encounter for long-term (current) use of aspirin | circulatory system | SMD | < 0.0001 | 0.66 (0.60 - 0.73) | 581 | 15238 |
| 573 | Other disorders of liver | digestive | SMI | < 0.0001 | 0.74 (0.68 - 0.80) | 830 | 13915 |
| 272 | Disorders of lipoid metabolism | endocrine/metabolic | SMD | < 0.0001 | 0.81 (0.77 - 0.86) | 2884 | 14755 |
| 255 | Disorders of adrenal glands | endocrine/metabolic | SMD | < 0.0001 | 0.57 (0.49 - 0.66) | 260 | 16932 |
| 080 | Postoperative infection | infectious diseases | SMD | < 0.0001 | 0.57 (0.50 - 0.66) | 257 | 17208 |
| 250 | Diabetes mellitus | endocrine/metabolic | SMI | < 0.0001 | 1.21 (1.15 - 1.27) | 2395 | 14753 |
| 426.92 | Cardiac defibrillator in situ | circulatory system | SMD | < 0.0001 | 0.45 (0.36 - 0.55) | 125 | 12600 |
| 851 | Complications of transplants and reattached limbs | injuries & poisonings | SMD | < 0.0001 | 0.44 (0.36 - 0.55) | 104 | 16969 |
| 401.3 | Other hypertensive complications | circulatory system | SMD | < 0.0001 | 0.47 (0.39 - 0.57) | 139 | 13217 |
| 411.2 | Myocardial infarction | circulatory system | SMD | < 0.0001 | 0.62 (0.55 - 0.70) | 423 | 16264 |
| 345 | Epilepsy, recurrent seizures, convulsions | neurological | SMI | < 0.0001 | 0.66 (0.59 - 0.73) | 379 | 16355 |
| 426.91 | Cardiac pacemaker in situ | circulatory system | SMD | < 0.0001 | 0.50 (0.42 - 0.60) | 211 | 12600 |
| 797 | Shock | symptoms | SMD | < 0.0001 | 0.50 (0.42 - 0.60) | 160 | 17479 |
| 573.5 | Jaundice (not of newborn) | digestive | SMD | < 0.0001 | 0.60 (0.53 - 0.69) | 331 | 13909 |
| 573.2 | Liver replaced by transplant | digestive | SMD | < 0.0001 | 0.51 (0.43 - 0.60) | 165 | 13909 |
| 571.81 | Portal hypertension | digestive | SMD | < 0.0001 | 0.60 (0.53 - 0.68) | 319 | 13909 |
| 287 | Purpura and other hemorrhagic conditions | hematopoietic | SMI | < 0.0001 | 0.72 (0.66 - 0.78) | 617 | 16149 |
| 427.21 | Atrial fibrillation | circulatory system | SMI | < 0.0001 | 0.74 (0.68 - 0.80) | 1180 | 12606 |
| 586 | Other disorders of the kidney and ureters | genitourinary | SMD | < 0.0001 | 0.72 (0.66 - 0.78) | 857 | 14818 |
| 790.6 | Other abnormal blood chemistry | symptoms | SMD | < 0.0001 | 0.72 (0.66 - 0.78) | 829 | 16690 |
| 117 | Mycoses | infectious diseases | SMI | < 0.0001 | 0.58 (0.51 - 0.67) | 189 | 16940 |
| 568.1 | Peritoneal adhesions (postoperative) (postinfection) | digestive | SMD | < 0.0001 | 0.57 (0.50 - 0.66) | 259 | 14829 |
| 275.3 | Disorders of magnesium metabolism | endocrine/metabolic | SMI | < 0.0001 | 0.61 (0.54 - 0.70) | 253 | 16974 |
| 495 | Asthma | respiratory | SMD | < 0.0001 | 0.72 (0.66 - 0.78) | 788 | 16322 |
| 550.6 | Incisional hernia | digestive | SMD | < 0.0001 | 0.54 (0.46 - 0.64) | 200 | 16378 |
| 536 | Disorders of function of stomach | digestive | SMI | < 0.0001 | 0.71 (0.65 - 0.78) | 554 | 16078 |
| 286.7 | Other and unspecified coagulation defects | hematopoietic | SMD | < 0.0001 | 0.53 (0.45 - 0.63) | 178 | 16143 |
| 558 | Noninfectious gastroenteritis | digestive | SMI | < 0.0001 | 0.75 (0.69 - 0.81) | 802 | 11805 |
| 327 | Sleep disorders | neurological | SMD | < 0.0001 | 0.74 (0.68 - 0.80) | 941 | 15923 |
| 260.2 | severe protein-calorie malnutrition | endocrine/metabolic | SMD | < 0.0001 | 0.62 (0.55 - 0.71) | 352 | 15668 |
| 275.5 | Disorders of calcium/phosphorus metabolism | endocrine/metabolic | SMI | < 0.0001 | 0.59 (0.52 - 0.68) | 213 | 16974 |
| 433 | Cerebrovascular disease | circulatory system | SMD | < 0.0001 | 0.71 (0.65 - 0.78) | 760 | 16754 |
| 743 | Osteoporosis, osteopenia and pathological fracture | musculoskeletal | SMI | < 0.0001 | 0.72 (0.66 - 0.79) | 728 | 16918 |
| 851 | Complications of transplants and reattached limbs | injuries & poisonings | SMI | < 0.0001 | 0.52 (0.44 - 0.62) | 104 | 16976 |
| 041.12 | Methicillin resistant Staphylococcus aureus | infectious diseases | SMD | < 0.0001 | 0.45 (0.36 - 0.55) | 113 | 15348 |
| 550.5 | Ventral hernia | digestive | SMI | < 0.0001 | 1.46 (1.32 - 1.61) | 431 | 16384 |
| 038.1 | Gram negative septicemia | infectious diseases | SMI | < 0.0001 | 0.58 (0.50 - 0.67) | 204 | 15355 |
| 561 | Symptoms involving digestive system | digestive | SMD | < 0.0001 | 0.80 (0.76 - 0.85) | 1959 | 11799 |
| 345.1 | Epilepsy | neurological | SMD | < 0.0001 | 0.49 (0.41 - 0.60) | 136 | 16348 |
| 532 | Dysphagia | digestive | SMI | < 0.0001 | 0.71 (0.65 - 0.78) | 569 | 14903 |
| 575.1 | Cholangitis | digestive | SMI | < 0.0001 | 0.63 (0.56 - 0.72) | 291 | 15847 |
| 575.8 | Other disorders of biliary tract | digestive | SMI | < 0.0001 | 0.71 (0.65 - 0.78) | 558 | 15847 |
| 316 | Substance addiction and disorders | mental disorders | SMD | < 0.0001 | 0.66 (0.59 - 0.74) | 443 | 15498 |
| 510 | Other diseases of lung | respiratory | SMD | < 0.0001 | 0.59 (0.51 - 0.68) | 255 | 17327 |
| 530.11 | GERD | digestive | SMD | < 0.0001 | 0.80 (0.75 - 0.85) | 1746 | 14896 |
| 276.11 | Hyperosmolality and/or hypernatremia | endocrine/metabolic | SMI | < 0.0001 | 0.50 (0.42 - 0.61) | 110 | 15001 |
| 574 | Cholelithiasis and cholecystitis | digestive | SMD | < 0.0001 | 0.75 (0.70 - 0.82) | 1049 | 15841 |
| 530.1 | Esophagitis, GERD and related diseases | digestive | SMD | < 0.0001 | 0.81 (0.77 - 0.86) | 2098 | 14896 |
| 573.3 | Hepatomegaly | digestive | SMD | < 0.0001 | 0.56 (0.47 - 0.65) | 201 | 13909 |
| 279.1 | Immunity deficiency | endocrine/metabolic | SMI | < 0.0001 | 0.56 (0.48 - 0.66) | 141 | 17084 |
| 562 | Diverticulosis and diverticulitis | digestive | SMI | < 0.0001 | 1.25 (1.17 - 1.33) | 1488 | 11805 |
| 501 | Pneumonitis due to inhalation of food or vomitus | respiratory | SMD | < 0.0001 | 0.53 (0.45 - 0.63) | 192 | 15342 |
| 797 | Shock | symptoms | SMI | < 0.0001 | 0.57 (0.49 - 0.67) | 160 | 17486 |
| 596 | Other disorders of bladder | genitourinary | SMD | < 0.0001 | 0.67 (0.60 - 0.75) | 451 | 16521 |
| 550 | Abdominal hernia | digestive | SMI | < 0.0001 | 1.25 (1.18 - 1.33) | 1262 | 16384 |
| 250.24 | Type 2 diabetes with neurological manifestations | endocrine/metabolic | SMD | < 0.0001 | 0.52 (0.43 - 0.63) | 162 | 14747 |
| 573.4 | Acute and subacute necrosis of liver | digestive | SMD | < 0.0001 | 0.50 (0.41 - 0.60) | 129 | 13909 |
| 345.3 | Convulsions | neurological | SMD | < 0.0001 | 0.56 (0.48 - 0.66) | 205 | 16348 |
| 859 | Complication due to other implant and internal device | injuries & poisonings | SMI | < 0.0001 | 0.59 (0.51 - 0.68) | 185 | 16976 |
| 512.9 | Other dyspnea | respiratory | SMI | < 0.0001 | 0.74 (0.68 - 0.81) | 704 | 15037 |
| 569.2 | Gastrointestinal complications | digestive | SMD | < 0.0001 | 0.62 (0.55 - 0.71) | 310 | 14829 |
| 250.42 | Other abnormal glucose | endocrine/metabolic | SMD | < 0.0001 | 0.69 (0.62 - 0.76) | 547 | 14747 |
| 425 | Cardiomyopathy | circulatory system | SMI | < 0.0001 | 0.66 (0.59 - 0.74) | 332 | 17109 |
| 592.1 | Cystitis | genitourinary | SMD | < 0.0001 | 0.69 (0.62 - 0.77) | 543 | 15562 |
| 573.9 | Abnormal serum enzyme levels | digestive | SMD | < 0.0001 | 0.65 (0.58 - 0.74) | 385 | 13909 |
| 244 | Hypothyroidism | endocrine/metabolic | SMD | < 0.0001 | 0.77 (0.72 - 0.83) | 1245 | 16096 |
| 592 | Cystitis and urethritis | genitourinary | SMD | < 0.0001 | 0.69 (0.63 - 0.77) | 555 | 15562 |
| 275.53 | Disorders of phosphorus metabolism | endocrine/metabolic | SMI | < 0.0001 | 0.53 (0.45 - 0.64) | 110 | 16974 |
| 008.52 | Intestinal infection due to C. difficile | infectious diseases | SMD | < 0.0001 | 0.63 (0.55 - 0.72) | 314 | 17033 |
| 510 | Other diseases of lung | respiratory | SMI | < 0.0001 | 0.64 (0.56 - 0.73) | 255 | 17334 |
| 426.2 | Atrioventricular [AV] block | circulatory system | SMD | < 0.0001 | 0.54 (0.46 - 0.65) | 203 | 12600 |
| 157 | Pancreatic cancer | neoplasms | SMI | < 0.0001 | 0.69 (0.62 - 0.77) | 440 | 15030 |
| 686 | Other local infections of skin and subcutaneous tissue | dermatologic | SMD | < 0.0001 | 0.48 (0.39 - 0.60) | 114 | 16721 |
| 041.12 | Methicillin resistant Staphylococcus aureus | infectious diseases | SMI | < 0.0001 | 0.53 (0.45 - 0.64) | 113 | 15355 |
| 426.32 | Left bundle branch block | circulatory system | SMD | < 0.0001 | 0.57 (0.48 - 0.67) | 239 | 12600 |
| 427.61 | Supraventricular premature beats | circulatory system | SMI | < 0.0001 | 0.55 (0.46 - 0.65) | 191 | 12606 |
| 587 | Kidney replaced by transpant | genitourinary | SMD | < 0.0001 | 0.51 (0.42 - 0.62) | 138 | 14818 |
| 153 | Colorectal cancer | neoplasms | SMI | < 0.0001 | 0.78 (0.73 - 0.84) | 1002 | 14717 |
| 496 | Chronic airway obstruction | respiratory | SMD | < 0.0001 | 0.70 (0.63 - 0.78) | 577 | 16322 |
| 564 | Functional digestive disorders | digestive | SMI | < 0.0001 | 0.78 (0.72 - 0.84) | 929 | 11805 |
| 204.2 | Myeloid leukemia | neoplasms | SMD | < 0.0001 | 0.58 (0.50 - 0.68) | 213 | 16677 |
| 280.2 | Iron deficiency anemia secondary to blood loss (chronic) | hematopoietic | SMI | < 0.0001 | 0.66 (0.58 - 0.75) | 307 | 14706 |
| 433.2 | Occlusion of cerebral arteries | circulatory system | SMD | < 0.0001 | 0.60 (0.52 - 0.70) | 260 | 16754 |
| 244.4 | Hypothyroidism NOS | endocrine/metabolic | SMD | < 0.0001 | 0.77 (0.71 - 0.83) | 1116 | 16096 |
| 426.8 | Other cardiac conduction disorders | circulatory system | SMI | < 0.0001 | 0.63 (0.55 - 0.72) | 236 | 12606 |
| 081 | Infection/inflammation of internal prosthetic device; implant; and graft | infectious diseases | SMI | < 0.0001 | 0.61 (0.53 - 0.71) | 181 | 17215 |
| 960.2 | Allergy/adverse effect of penicillin | injuries & poisonings | SMD | < 0.0001 | 0.66 (0.59 - 0.75) | 386 | 15985 |
| 433.21 | Cerebral artery occlusion, with cerebral infarction | circulatory system | SMD | < 0.0001 | 0.60 (0.52 - 0.70) | 255 | 16754 |
| 571.8 | Liver abscess and sequelae of chronic liver disease | digestive | SMI | < 0.0001 | 0.75 (0.69 - 0.82) | 661 | 13915 |
| 480.1 | Bacterial pneumonia | respiratory | SMD | < 0.0001 | 0.51 (0.42 - 0.62) | 126 | 16737 |
| 573.9 | Abnormal serum enzyme levels | digestive | SMI | < 0.0001 | 0.70 (0.63 - 0.78) | 386 | 13915 |
| 785 | Abdominal pain | symptoms | SMI | < 0.0001 | 1.12 (1.08 - 1.16) | 11048 | 6598 |
| 277.4 | Disorders of bilirubin excretion | endocrine/metabolic | SMI | < 0.0001 | 0.59 (0.51 - 0.69) | 157 | 17309 |
| 270.38 | Other specified disorders of plasma protein metabolism | endocrine/metabolic | SMI | < 0.0001 | 0.55 (0.46 - 0.66) | 112 | 17328 |
| 008.5 | Bacterial enteritis | infectious diseases | SMD | < 0.0001 | 0.67 (0.59 - 0.76) | 373 | 17033 |
| 315 | Develomental delays and disorders | mental disorders | SMD | < 0.0001 | 0.51 (0.41 - 0.63) | 111 | 17444 |
| 428.4 | Heart failure with preserved EF [Diastolic heart failure] | circulatory system | SMD | < 0.0001 | 0.51 (0.42 - 0.63) | 148 | 16703 |
| 782.3 | Edema | symptoms | SMI | < 0.0001 | 0.78 (0.72 - 0.84) | 878 | 16734 |
| 250.4 | Abnormal glucose | endocrine/metabolic | SMD | < 0.0001 | 0.73 (0.66 - 0.80) | 666 | 14747 |
| 270.38 | Other specified disorders of plasma protein metabolism | endocrine/metabolic | SMD | < 0.0001 | 0.50 (0.41 - 0.62) | 112 | 17321 |
| 427.11 | Paroxysmal supraventricular tachycardia | circulatory system | SMD | < 0.0001 | 0.55 (0.46 - 0.66) | 170 | 12600 |
| 429.1 | Heart transplant/surgery | circulatory system | SMI | < 0.0001 | 0.55 (0.46 - 0.66) | 102 | 16709 |
| 537 | Other disorders of stomach and duodenum | digestive | SMI | < 0.0001 | 0.76 (0.70 - 0.83) | 666 | 16078 |
| 628 | Ovarian cyst | genitourinary | SMD | < 0.0001 | 1.57 (1.37 - 1.81) | 383 | 9271 |
| 960 | Poisoning by antibiotics | injuries & poisonings | SMI | < 0.0001 | 0.77 (0.71 - 0.83) | 697 | 15992 |
| 198.6 | Secondary malignancy of bone | neoplasms | SMD | < 0.0001 | 0.70 (0.62 - 0.78) | 446 | 14136 |
| 200 | Myeloproliferative disease | neoplasms | SMD | < 0.0001 | 0.63 (0.55 - 0.73) | 266 | 16677 |
| 513 | Respiratory abnormalities | respiratory | SMD | < 0.0001 | 0.55 (0.46 - 0.66) | 149 | 17490 |
| 041.1 | Staphylococcus infections | infectious diseases | SMI | < 0.0001 | 0.65 (0.56 - 0.74) | 227 | 15355 |
| 270 | Disorders of protein plasma/amino-acid transport and metabolism | endocrine/metabolic | SMD | < 0.0001 | 0.59 (0.51 - 0.70) | 200 | 17321 |
| 592.11 | Acute cystitis | genitourinary | SMD | < 0.0001 | 0.70 (0.62 - 0.78) | 458 | 15562 |
| 280.1 | Iron deficiency anemias, unspecified or not due to blood loss | hematopoietic | SMI | < 0.0001 | 0.75 (0.69 - 0.82) | 599 | 14706 |
| 204.21 | Myeloid leukemia, acute | neoplasms | SMD | < 0.0001 | 0.59 (0.50 - 0.69) | 183 | 16677 |
| 008 | Intestinal infection | infectious diseases | SMD | < 0.0001 | 0.73 (0.67 - 0.81) | 606 | 17033 |
| 151 | Cancer of stomach | neoplasms | SMI | < 0.0001 | 0.65 (0.57 - 0.74) | 234 | 15030 |
| 286.7 | Other and unspecified coagulation defects | hematopoietic | SMI | < 0.0001 | 0.62 (0.54 - 0.73) | 178 | 16149 |
| 550.4 | Umbilical hernia | digestive | SMI | < 0.0001 | 1.58 (1.37 - 1.83) | 189 | 16384 |
| 590 | Pyelonephritis | genitourinary | SMD | < 0.0001 | 0.69 (0.62 - 0.78) | 428 | 15562 |
| 286 | Coagulation defects | hematopoietic | SMI | < 0.0001 | 0.74 (0.67 - 0.81) | 476 | 16149 |
| 585.32 | End stage renal disease | genitourinary | SMI | < 0.0001 | 0.68 (0.60 - 0.77) | 293 | 14825 |
| 279.1 | Immunity deficiency | endocrine/metabolic | SMD | < 0.0001 | 0.56 (0.46 - 0.67) | 141 | 17077 |
| 573.6 | Nonspecific elevation of levels of transaminase or lactic acid dehydrogenase [LDH] | digestive | SMI | < 0.0001 | 0.71 (0.63 - 0.79) | 349 | 13915 |
| 426.21 | First degree AV block | circulatory system | SMD | < 0.0001 | 0.51 (0.42 - 0.64) | 135 | 12600 |
| 345.1 | Epilepsy | neurological | SMI | < 0.0001 | 0.59 (0.50 - 0.70) | 136 | 16355 |
| 276.6 | Fluid overload | endocrine/metabolic | SMI | < 0.0001 | 0.67 (0.59 - 0.76) | 275 | 15001 |
| 963.1 | Antineoplastic and immunosuppressive drugs causing adverse effects | injuries & poisonings | SMI | < 0.0001 | 0.66 (0.58 - 0.76) | 233 | 15992 |
| 253 | Disorders of the pituitary gland and its hypothalamic control | endocrine/metabolic | SMD | < 0.0001 | 0.50 (0.41 - 0.63) | 103 | 16932 |
| 560 | Intestinal obstruction without mention of hernia | digestive | SMD | < 0.0001 | 0.82 (0.77 - 0.88) | 1698 | 11799 |
| 198.1 | Secondary malignancy of lymph nodes | neoplasms | SMI | < 0.0001 | 0.76 (0.70 - 0.83) | 612 | 14143 |
| 596.5 | Functional disorders of bladder | genitourinary | SMD | < 0.0001 | 0.60 (0.51 - 0.71) | 198 | 16521 |
| 433.8 | Late effects of cerebrovascular disease | circulatory system | SMD | < 0.0001 | 0.53 (0.43 - 0.65) | 132 | 16754 |
| 591 | Urinary tract infection | genitourinary | SMI | < 0.0001 | 0.82 (0.76 - 0.87) | 1188 | 15566 |
| 274.1 | Gout | endocrine/metabolic | SMD | < 0.0001 | 0.58 (0.49 - 0.70) | 184 | 17437 |
| 345.3 | Convulsions | neurological | SMI | < 0.0001 | 0.64 (0.56 - 0.74) | 205 | 16355 |
| 963 | Poisoning by primarily systemic agents | injuries & poisonings | SMI | < 0.0001 | 0.67 (0.59 - 0.76) | 238 | 15992 |
| 277.4 | Disorders of bilirubin excretion | endocrine/metabolic | SMD | < 0.0001 | 0.58 (0.48 - 0.69) | 157 | 17303 |
| 280.2 | Iron deficiency anemia secondary to blood loss (chronic) | hematopoietic | SMD | < 0.0001 | 0.66 (0.57 - 0.75) | 307 | 14700 |
| 540.11 | Acute appendicitis | digestive | SMD | < 0.0001 | 1.43 (1.27 - 1.61) | 525 | 17039 |
| 270.3 | Disorders of plasma protein metabolism | endocrine/metabolic | SMD | < 0.0001 | 0.59 (0.50 - 0.70) | 177 | 17321 |
| 550.6 | Incisional hernia | digestive | SMI | < 0.0001 | 1.54 (1.34 - 1.78) | 200 | 16384 |
| 579.2 | Splenomegaly | digestive | SMD | < 0.0001 | 0.66 (0.58 - 0.76) | 300 | 15478 |
| 420 | Carditis | circulatory system | SMD | < 0.0001 | 0.63 (0.54 - 0.73) | 237 | 17102 |
| 425.1 | Primary/intrinsic cardiomyopathies | circulatory system | SMI | < 0.0001 | 0.68 (0.60 - 0.77) | 278 | 17109 |
| 580 | Nephritis; nephrosis; renal sclerosis | genitourinary | SMD | < 0.0001 | 0.56 (0.46 - 0.68) | 139 | 14818 |
| 427.1 | Paroxysmal tachycardia, unspecified | circulatory system | SMI | < 0.0001 | 0.66 (0.58 - 0.76) | 278 | 12606 |
| 585.3 | Chronic renal failure [CKD] | genitourinary | SMI | < 0.0001 | 0.78 (0.72 - 0.85) | 828 | 14825 |
| 274 | Gout and other crystal arthropathies | endocrine/metabolic | SMD | < 0.0001 | 0.60 (0.51 - 0.71) | 202 | 17437 |
| 740 | Osteoarthrosis | musculoskeletal | SMD | < 0.0001 | 0.73 (0.66 - 0.81) | 564 | 17075 |
| 990 | Effects radiation NOS | injuries & poisonings | SMD | < 0.0001 | 0.72 (0.65 - 0.81) | 547 | 15569 |
| 250.1 | Type 1 diabetes | endocrine/metabolic | SMD | < 0.0001 | 0.57 (0.47 - 0.69) | 147 | 14747 |
| 300.1 | Anxiety disorder | mental disorders | SMI | < 0.0001 | 0.83 (0.78 - 0.88) | 1281 | 14166 |
| 578 | Gastrointestinal hemorrhage | digestive | SMI | < 0.0001 | 0.82 (0.77 - 0.88) | 1127 | 15485 |
| 555.21 | Ulcerative colitis (chronic) | digestive | SMI | < 0.0001 | 0.61 (0.52 - 0.72) | 135 | 11805 |
| 195.1 | Malignant neoplasm, other | neoplasms | SMD | < 0.0001 | 0.76 (0.69 - 0.83) | 707 | 14136 |
| 255.2 | Adrenal hypofunction | endocrine/metabolic | SMI | < 0.0001 | 0.58 (0.49 - 0.70) | 118 | 16939 |
| 255.21 | Glucocorticoid deficiency | endocrine/metabolic | SMI | < 0.0001 | 0.58 (0.49 - 0.70) | 118 | 16939 |
| 349 | Other and unspecified disorders of the nervous system | neurological | SMD | < 0.0001 | 0.51 (0.41 - 0.64) | 104 | 16348 |
| 415 | Pulmonary heart disease | circulatory system | SMI | < 0.0001 | 0.79 (0.73 - 0.85) | 770 | 16494 |
| 743.1 | Osteoporosis | musculoskeletal | SMI | < 0.0001 | 0.70 (0.63 - 0.79) | 407 | 16918 |
| 198.3 | Secondary malignant neoplasm of digestive systems | neoplasms | SMD | < 0.0001 | 0.75 (0.68 - 0.83) | 653 | 14136 |
| 411.8 | Other chronic ischemic heart disease, unspecified | circulatory system | SMD | < 0.0001 | 0.50 (0.40 - 0.64) | 103 | 16264 |
| 426.9 | Cardiac pacemaker/device in situ | circulatory system | SMI | < 0.0001 | 0.67 (0.58 - 0.76) | 313 | 12606 |
| 506 | Empyema and pneumothorax | respiratory | SMD | < 0.0001 | 0.65 (0.56 - 0.75) | 267 | 15342 |
| 204 | Leukemia | neoplasms | SMI | < 0.0001 | 0.74 (0.67 - 0.82) | 440 | 16684 |
| 595 | Hydronephrosis | genitourinary | SMI | < 0.0001 | 0.78 (0.72 - 0.85) | 680 | 16294 |
| 573.6 | Nonspecific elevation of levels of transaminase or lactic acid dehydrogenase [LDH] | digestive | SMD | < 0.0001 | 0.69 (0.60 - 0.78) | 349 | 13909 |
| 977 | Personal history of allergy to medicinal agents | injuries & poisonings | SMI | < 0.0001 | 0.75 (0.68 - 0.82) | 460 | 15992 |
| 280.1 | Iron deficiency anemias, unspecified or not due to blood loss | hematopoietic | SMD | < 0.0001 | 0.74 (0.67 - 0.82) | 599 | 14700 |
| 153.3 | Malignant neoplasm of rectum, rectosigmoid junction, and anus | neoplasms | SMI | < 0.0001 | 0.76 (0.69 - 0.83) | 506 | 14717 |
| 509.8 | Dependence on respirator [Ventilator] or supplemental oxygen | respiratory | SMI | < 0.0001 | 0.59 (0.49 - 0.71) | 119 | 15349 |
| 860 | Bone marrow or stem cell transplant | neoplasms | SMI | < 0.0001 | 0.68 (0.59 - 0.77) | 216 | 17430 |
| 270 | Disorders of protein plasma/amino-acid transport and metabolism | endocrine/metabolic | SMI | < 0.0001 | 0.65 (0.57 - 0.76) | 200 | 17328 |
| 772.3 | Muscle weakness | symptoms | SMI | < 0.0001 | 0.69 (0.60 - 0.78) | 266 | 17215 |
| 577.1 | Acute pancreatitis | digestive | SMD | < 0.0001 | 0.73 (0.66 - 0.82) | 495 | 16514 |
| 285.21 | Anemia in chronic kidney disease | hematopoietic | SMI | < 0.0001 | 0.64 (0.55 - 0.75) | 178 | 14706 |
| 740.1 | Osteoarthritis; localized | musculoskeletal | SMI | < 0.0001 | 1.42 (1.26 - 1.60) | 318 | 17082 |
| 159 | Malignant neoplasm of other and ill-defined sites within the digestive organs and peritoneum | neoplasms | SMI | < 0.0001 | 0.77 (0.71 - 0.85) | 627 | 15030 |
| 112 | Candidiasis | infectious diseases | SMI | < 0.0001 | 0.73 (0.66 - 0.82) | 370 | 16940 |
| 565 | Anal and rectal conditions | digestive | SMI | < 0.0001 | 0.77 (0.71 - 0.85) | 541 | 14836 |
| 577 | Diseases of pancreas | digestive | SMD | < 0.0001 | 0.81 (0.75 - 0.87) | 1125 | 16514 |
| 574.1 | Cholelithiasis | digestive | SMD | < 0.0001 | 0.78 (0.71 - 0.85) | 783 | 15841 |
| 562.2 | Diverticulitis | digestive | SMI | < 0.0001 | 1.26 (1.16 - 1.36) | 755 | 11805 |
| 153.2 | Colon cancer | neoplasms | SMI | < 0.0001 | 0.79 (0.72 - 0.86) | 695 | 14717 |
| 575.7 | Other disorders of gallbladder | digestive | SMD | < 0.0001 | 0.62 (0.53 - 0.74) | 192 | 15841 |
| 575.2 | Obstruction of bile duct | digestive | SMD | < 0.0001 | 0.72 (0.64 - 0.81) | 412 | 15841 |
| 696.4 | Psoriasis | dermatologic | SMD | < 0.0001 | 0.54 (0.43 - 0.67) | 106 | 16759 |
| 249 | Secondary diabetes mellitus | endocrine/metabolic | SMD | < 0.0001 | 0.60 (0.50 - 0.72) | 164 | 14747 |
| 327.6 | Circadian rhythm sleep disorder | neurological | SMI | < 0.0001 | 0.60 (0.50 - 0.72) | 118 | 15928 |
| 743.11 | Osteoporosis NOS | musculoskeletal | SMI | < 0.0001 | 0.71 (0.63 - 0.80) | 384 | 16918 |
| 270.3 | Disorders of plasma protein metabolism | endocrine/metabolic | SMI | < 0.0001 | 0.64 (0.55 - 0.75) | 177 | 17328 |
| 428.3 | Heart failure with reduced EF [Systolic or combined heart failure] | circulatory system | SMI | < 0.0001 | 0.73 (0.66 - 0.82) | 414 | 16709 |
| 560.3 | Peritoneal or intestinal adhesions | digestive | SMI | < 0.0001 | 0.62 (0.52 - 0.73) | 142 | 11805 |
| 349 | Other and unspecified disorders of the nervous system | neurological | SMI | < 0.0001 | 0.58 (0.48 - 0.71) | 104 | 16355 |
| 357 | Inflammatory and toxic neuropathy | neurological | SMD | < 0.0001 | 0.62 (0.52 - 0.74) | 179 | 17286 |
| 443 | Peripheral vascular disease | circulatory system | SMD | < 0.0001 | 0.64 (0.55 - 0.75) | 226 | 16717 |
| 415.1 | Acute pulmonary heart disease | circulatory system | SMI | < 0.0001 | 0.74 (0.66 - 0.82) | 394 | 16494 |
| 495 | Asthma | respiratory | SMI | < 0.0001 | 1.23 (1.14 - 1.33) | 788 | 16329 |
| 355.1 | Chronic pain syndrome | neurological | SMD | < 0.0001 | 0.60 (0.50 - 0.72) | 148 | 17138 |
| 532 | Dysphagia | digestive | SMD | < 0.0001 | 0.75 (0.68 - 0.84) | 569 | 14896 |
| 963 | Poisoning by primarily systemic agents | injuries & poisonings | SMD | 0.00011 | 0.66 (0.57 - 0.77) | 238 | 15985 |
| 204.1 | Lymphoid leukemia | neoplasms | SMI | 0.00011 | 0.65 (0.56 - 0.76) | 159 | 16684 |
| 564 | Functional digestive disorders | digestive | SMD | 0.00012 | 0.80 (0.74 - 0.87) | 929 | 11799 |
| 540.1 | Appendicitis | digestive | SMD | 0.00012 | 1.36 (1.21 - 1.52) | 567 | 17039 |
| 415.11 | Pulmonary embolism and infarction, acute | circulatory system | SMI | 0.00012 | 0.74 (0.66 - 0.83) | 392 | 16494 |
| 079 | Viral infection | infectious diseases | SMI | 0.00013 | 0.72 (0.64 - 0.81) | 283 | 16473 |
| 396 | Abnormal heart sounds | circulatory system | SMD | 0.00014 | 0.54 (0.43 - 0.68) | 102 | 17028 |
| 540 | Appendiceal conditions | digestive | SMD | 0.00016 | 1.34 (1.20 - 1.50) | 600 | 17039 |
| 459 | Other disorders of circulatory system | circulatory system | SMD | 0.00016 | 0.70 (0.61 - 0.80) | 316 | 16641 |
| 427.11 | Paroxysmal supraventricular tachycardia | circulatory system | SMI | 0.00016 | 0.63 (0.53 - 0.75) | 170 | 12606 |
| 578 | Gastrointestinal hemorrhage | digestive | SMD | 0.00016 | 0.82 (0.76 - 0.88) | 1127 | 15478 |
| 796 | Elevated prostate specific antigen [PSA] | genitourinary | SMI | 0.00018 | 1.84 (1.47 - 2.31) | 108 | 6261 |
| 696 | Psoriasis and related disorders | dermatologic | SMD | 0.00018 | 0.56 (0.46 - 0.70) | 112 | 16759 |
| 963.1 | Antineoplastic and immunosuppressive drugs causing adverse effects | injuries & poisonings | SMD | 0.00019 | 0.66 (0.57 - 0.77) | 233 | 15985 |
| 300 | Anxiety disorders | mental disorders | SMI | 0.00023 | 0.86 (0.81 - 0.91) | 1502 | 14166 |
| 710 | Osteomyelitis, periostitis, and other infections involving bone | musculoskeletal | SMI | 0.00023 | 0.64 (0.54 - 0.76) | 155 | 17069 |
| 285.22 | Anemia in neoplastic disease | hematopoietic | SMD | 0.00023 | 0.63 (0.54 - 0.75) | 198 | 14700 |
| 198.2 | Secondary malignancy of respiratory organs | neoplasms | SMD | 0.00023 | 0.73 (0.65 - 0.82) | 408 | 14136 |
| 454 | Varicose veins | circulatory system | SMD | 0.00028 | 0.60 (0.50 - 0.73) | 145 | 15238 |
| 596 | Other disorders of bladder | genitourinary | SMI | 0.00029 | 0.76 (0.69 - 0.85) | 451 | 16528 |
| 562.1 | Diverticulosis | digestive | SMI | 0.00031 | 1.23 (1.14 - 1.33) | 886 | 11805 |
| 433.31 | Transient cerebral ischemia | circulatory system | SMD | 0.00031 | 0.71 (0.62 - 0.81) | 358 | 16754 |
| 771 | Musculoskeletal symptoms referable to limbs | symptoms | SMD | 0.00032 | 0.63 (0.53 - 0.75) | 177 | 17137 |
| 571.5 | Other chronic nonalcoholic liver disease | digestive | SMI | 0.00032 | 1.17 (1.10 - 1.24) | 1386 | 13915 |
| 710.1 | Osteomyelitis | musculoskeletal | SMI | 0.00034 | 0.64 (0.55 - 0.76) | 154 | 17069 |
| 433.3 | Cerebral ischemia | circulatory system | SMD | 0.00036 | 0.72 (0.63 - 0.81) | 377 | 16754 |
| 502 | Postinflammatory pulmonary fibrosis | respiratory | SMD | 0.00037 | 0.58 (0.47 - 0.72) | 128 | 15342 |
| 189.1 | Cancer of kidney and renal pelvis | neoplasms | SMI | 0.00046 | 1.40 (1.23 - 1.59) | 275 | 17039 |
| 428 | Congestive heart failure; nonhypertensive | circulatory system | SMI | 0.00047 | 0.81 (0.75 - 0.88) | 821 | 16709 |
| 427.12 | Paroxysmal ventricular tachycardia | circulatory system | SMD | 0.00048 | 0.54 (0.43 - 0.69) | 101 | 12600 |
| 459 | Other disorders of circulatory system | circulatory system | SMI | 0.00060 | 0.74 (0.65 - 0.83) | 317 | 16647 |
| 596.5 | Functional disorders of bladder | genitourinary | SMI | 0.00063 | 0.68 (0.59 - 0.79) | 198 | 16528 |
| 578.1 | Hematemesis | digestive | SMD | 0.00065 | 0.59 (0.48 - 0.73) | 126 | 15478 |
| 327 | Sleep disorders | neurological | SMI | 0.00068 | 0.83 (0.77 - 0.89) | 943 | 15928 |
| 459.9 | Circulatory disease NEC | circulatory system | SMD | 0.00071 | 0.59 (0.48 - 0.72) | 124 | 16641 |
| 771.1 | Swelling of limb | symptoms | SMD | 0.00078 | 0.71 (0.62 - 0.81) | 308 | 17137 |
| 276.13 | Hyperpotassemia | endocrine/metabolic | SMI | 0.00082 | 0.68 (0.59 - 0.79) | 202 | 15001 |
| 327.4 | Insomnia | neurological | SMI | 0.00085 | 0.80 (0.73 - 0.87) | 619 | 15928 |
| 260.3 | Adult failure to thrive | endocrine/metabolic | SMD | 0.00086 | 0.68 (0.58 - 0.79) | 244 | 15668 |
| 427.5 | Arrhythmia (cardiac) NOS | circulatory system | SMD | 0.00087 | 0.58 (0.47 - 0.72) | 124 | 12600 |
| 740.11 | Osteoarthrosis, localized, primary | musculoskeletal | SMI | 0.00092 | 1.40 (1.22 - 1.59) | 274 | 17082 |
| 289.5 | Diseases of spleen | hematopoietic | SMD | 0.00097 | 0.63 (0.53 - 0.76) | 163 | 15441 |
| 577 | Diseases of pancreas | digestive | SMI | 0.0013 | 0.85 (0.79 - 0.91) | 1125 | 16521 |
| 333 | Extrapyramidal disease and abnormal movement disorders | neurological | SMI | 0.0014 | 0.62 (0.52 - 0.76) | 126 | 16355 |
| 559 | Ileostomy status | digestive | SMD | 0.0015 | 0.70 (0.61 - 0.81) | 267 | 11799 |
| 418 | Nonspecific chest pain | circulatory system | SMD | 0.0015 | 0.85 (0.79 - 0.91) | 1424 | 16206 |
| 568 | Other disorders of peritoneum | digestive | SMI | 0.0016 | 0.80 (0.73 - 0.87) | 548 | 14836 |
| 296.1 | Bipolar | mental disorders | SMD | 0.0016 | 0.69 (0.59 - 0.80) | 249 | 14160 |
| 430 | Intracranial hemorrhage | circulatory system | SMD | 0.0016 | 0.67 (0.57 - 0.79) | 207 | 16754 |
| 625 | Pain and other symptoms associated with female genital organs | genitourinary | SMD | 0.0017 | 1.41 (1.23 - 1.62) | 366 | 9710 |
| 427.5 | Arrhythmia (cardiac) NOS | circulatory system | SMI | 0.0018 | 0.62 (0.51 - 0.75) | 124 | 12606 |
| 348.2 | Cerebral edema and compression of brain | neurological | SMD | 0.0018 | 0.59 (0.47 - 0.73) | 112 | 16348 |
| 430 | Intracranial hemorrhage | circulatory system | SMI | 0.0020 | 0.70 (0.60 - 0.81) | 207 | 16761 |
| 729 | Other disorders of soft tissues | musculoskeletal | SMD | 0.0021 | 0.68 (0.58 - 0.80) | 227 | 16966 |
| 740 | Osteoarthrosis | musculoskeletal | SMI | 0.0022 | 1.26 (1.15 - 1.39) | 564 | 17082 |
| 459.9 | Circulatory disease NEC | circulatory system | SMI | 0.0026 | 0.64 (0.53 - 0.77) | 125 | 16647 |
| 870 | Open wounds of head; neck; and trunk | injuries & poisonings | SMD | 0.0026 | 0.71 (0.62 - 0.82) | 275 | 17221 |
| 789 | Nausea and vomiting | symptoms | SMD | 0.0027 | 0.90 (0.86 - 0.94) | 4547 | 13092 |
| 577.2 | Chronic pancreatitis | digestive | SMD | 0.0030 | 0.66 (0.56 - 0.79) | 178 | 16514 |
| 710.19 | Unspecified osteomyelitis | musculoskeletal | SMI | 0.0031 | 0.64 (0.53 - 0.77) | 122 | 17069 |
| 426.3 | Bundle branch block | circulatory system | SMI | 0.0032 | 0.77 (0.69 - 0.86) | 539 | 12606 |
| 315 | Develomental delays and disorders | mental disorders | SMI | 0.0035 | 0.64 (0.53 - 0.77) | 111 | 17451 |
| 771 | Musculoskeletal symptoms referable to limbs | symptoms | SMI | 0.0036 | 0.68 (0.59 - 0.80) | 177 | 17144 |
| 740.1 | Osteoarthritis; localized | musculoskeletal | SMD | 0.0036 | 0.72 (0.63 - 0.83) | 318 | 17075 |
| 287.31 | Primary thrombocytopenia | hematopoietic | SMD | 0.0036 | 0.59 (0.48 - 0.74) | 110 | 16143 |
| 594 | Urinary calculus | genitourinary | SMI | 0.0037 | 1.19 (1.10 - 1.27) | 883 | 16294 |
| 165 | Cancer within the respiratory system | neoplasms | SMI | 0.0037 | 0.71 (0.61 - 0.82) | 233 | 17403 |
| 528 | Diseases of the oral soft tissues, excluding lesions specific for gingiva and tongue | digestive | SMI | 0.0037 | 0.68 (0.58 - 0.80) | 161 | 17385 |
| 427.22 | Atrial flutter | circulatory system | SMI | 0.0040 | 0.69 (0.59 - 0.81) | 235 | 12606 |
| 189.11 | Malignant neoplasm of kidney, except pelvis | neoplasms | SMI | 0.0041 | 1.41 (1.22 - 1.62) | 220 | 17039 |
| 290.1 | Dementias | mental disorders | SMI | 0.0046 | 0.69 (0.59 - 0.81) | 312 | 16104 |
| 994.1 | Systemic inflammatory response syndrome (SIRS) | injuries & poisonings | SMI | 0.0047 | 0.66 (0.55 - 0.79) | 135 | 15970 |
| 202 | Cancer of other lymphoid, histiocytic tissue | neoplasms | SMD | 0.0050 | 0.75 (0.66 - 0.85) | 391 | 16677 |
| 218 | Benign neoplasm of uterus | neoplasms | SMI | 0.0055 | 1.28 (1.15 - 1.41) | 384 | 8968 |
| 443.9 | Peripheral vascular disease, unspecified | circulatory system | SMD | 0.0055 | 0.65 (0.54 - 0.78) | 177 | 16717 |
| 512.8 | Cough | respiratory | SMD | 0.0069 | 0.81 (0.75 - 0.89) | 806 | 15031 |
| 165.1 | Cancer of bronchus; lung | neoplasms | SMI | 0.0074 | 0.71 (0.61 - 0.82) | 225 | 17403 |
| 594.1 | Calculus of kidney | genitourinary | SMI | 0.0077 | 1.20 (1.11 - 1.30) | 694 | 16294 |
| 395.6 | Heart valve replaced | circulatory system | SMD | 0.0081 | 0.62 (0.51 - 0.77) | 138 | 17028 |
| 871 | Open wounds of extremities | injuries & poisonings | SMD | 0.0082 | 0.65 (0.54 - 0.79) | 152 | 17221 |
| 200 | Myeloproliferative disease | neoplasms | SMI | 0.0086 | 0.74 (0.65 - 0.84) | 266 | 16684 |
| 292.1 | Aphasia/speech disturbance | mental disorders | SMD | 0.010 | 0.63 (0.51 - 0.77) | 139 | 16099 |
| 595 | Hydronephrosis | genitourinary | SMD | 0.011 | 0.81 (0.74 - 0.89) | 680 | 16288 |
| 271 | Disorders of carbohydrate transport and metabolism | endocrine/metabolic | SMI | 0.011 | 1.53 (1.26 - 1.84) | 116 | 17530 |
| 714.1 | Rheumatoid arthritis | musculoskeletal | SMD | 0.012 | 0.67 (0.56 - 0.80) | 184 | 17185 |
| 271.3 | Intestinal disaccharidase deficiencies and disaccharide malabsorption | endocrine/metabolic | SMI | 0.012 | 1.54 (1.27 - 1.85) | 113 | 17530 |
| 859 | Complication due to other implant and internal device | injuries & poisonings | SMD | 0.012 | 0.68 (0.57 - 0.81) | 185 | 16969 |
| 530.2 | Esophageal bleeding (varices/hemorrhage) | digestive | SMD | 0.014 | 0.70 (0.60 - 0.82) | 225 | 14896 |
| 575.8 | Other disorders of biliary tract | digestive | SMD | 0.014 | 0.79 (0.71 - 0.88) | 558 | 15841 |
| 306 | Other mental disorder | mental disorders | SMD | 0.014 | 0.74 (0.64 - 0.85) | 317 | 14160 |
| 585.31 | Renal dialysis | genitourinary | SMI | 0.015 | 0.74 (0.64 - 0.84) | 238 | 14825 |
| 939 | Atopic/contact dermatitis due to other or unspecified | dermatologic | SMD | 0.015 | 0.77 (0.68 - 0.87) | 416 | 16743 |
| 218.1 | Uterine leiomyoma | neoplasms | SMI | 0.015 | 1.26 (1.14 - 1.40) | 377 | 8968 |
| 136 | Other infectious and parasitic diseases | infectious diseases | SMD | 0.016 | 0.69 (0.59 - 0.82) | 203 | 17387 |
| 689 | Disorder of skin and subcutaneous tissue NOS | dermatologic | SMD | 0.017 | 0.68 (0.57 - 0.81) | 185 | 17454 |
| 805 | Fracture of vertebral column without mention of spinal cord injury | injuries & poisonings | SMI | 0.017 | 0.75 (0.66 - 0.85) | 283 | 16757 |
| 714 | Rheumatoid arthritis and other inflammatory polyarthropathies | musculoskeletal | SMD | 0.017 | 0.69 (0.59 - 0.82) | 212 | 17185 |
| 420 | Carditis | circulatory system | SMI | 0.017 | 0.74 (0.64 - 0.85) | 237 | 17109 |
| 182 | Malignant neoplasm of uterus | neoplasms | SMD | 0.017 | 0.72 (0.62 - 0.83) | 268 | 8577 |
| 185 | Cancer of prostate | neoplasms | SMI | 0.018 | 1.30 (1.16 - 1.47) | 483 | 6261 |
| 965 | Poisoning by analgesics, antipyretics, and antirheumatics | injuries & poisonings | SMD | 0.019 | 0.66 (0.55 - 0.80) | 164 | 15985 |
| 772.3 | Muscle weakness | symptoms | SMD | 0.019 | 0.72 (0.63 - 0.84) | 266 | 17208 |
| 189 | Cancer of urinary organs (incl. kidney and bladder) | neoplasms | SMI | 0.021 | 1.23 (1.12 - 1.35) | 607 | 17039 |
| 536.7 | Complications of gastrostomy, colostomy and enterostomy | digestive | SMD | 0.021 | 0.64 (0.53 - 0.79) | 132 | 16072 |
| 745 | Pain in joint | musculoskeletal | SMI | 0.021 | 1.13 (1.07 - 1.20) | 1533 | 16113 |
| 531 | Peptic ulcer (excl. esophageal) | digestive | SMI | 0.023 | 0.79 (0.71 - 0.88) | 410 | 17236 |
| 740.11 | Osteoarthrosis, localized, primary | musculoskeletal | SMD | 0.024 | 0.72 (0.62 - 0.84) | 274 | 17075 |
| 587 | Kidney replaced by transpant | genitourinary | SMI | 0.025 | 0.69 (0.58 - 0.82) | 138 | 14825 |
| 512 | Other symptoms of respiratory system | respiratory | SMI | 0.026 | 0.91 (0.87 - 0.95) | 2609 | 15037 |
| 458.1 | Orthostatic hypotension | circulatory system | SMI | 0.029 | 0.65 (0.53 - 0.80) | 109 | 16647 |
| 426.31 | Right bundle branch block | circulatory system | SMI | 0.031 | 0.72 (0.62 - 0.84) | 265 | 12606 |
| 579 | Other symptoms involving abdomen and pelvis | digestive | SMD | 0.031 | 0.84 (0.77 - 0.91) | 932 | 15478 |
| 709 | Diffuse diseases of connective tissue | dermatologic | SMD | 0.031 | 0.67 (0.55 - 0.81) | 156 | 16820 |
| 573.2 | Liver replaced by transplant | digestive | SMI | 0.031 | 0.71 (0.60 - 0.83) | 165 | 13915 |
| 277 | Other disorders of metabolism | endocrine/metabolic | SMI | 0.032 | 0.68 (0.57 - 0.82) | 128 | 17309 |
| 289.4 | Lymphadenitis | hematopoietic | SMD | 0.035 | 0.83 (0.75 - 0.90) | 766 | 15441 |
| 764 | Sciatica | symptoms | SMD | 0.035 | 0.72 (0.62 - 0.84) | 240 | 17093 |
| 202 | Cancer of other lymphoid, histiocytic tissue | neoplasms | SMI | 0.036 | 0.79 (0.71 - 0.88) | 391 | 16684 |
| 574.3 | Cholecystitis without cholelithiasis | digestive | SMD | 0.037 | 0.76 (0.66 - 0.86) | 334 | 15841 |
| 348.9 | Other conditions of brain, NOS | neurological | SMI | 0.038 | 0.68 (0.57 - 0.82) | 139 | 16355 |
| 536.3 | Gastroparesis | digestive | SMI | 0.038 | 0.76 (0.66 - 0.86) | 239 | 16078 |
| 599.4 | Urinary incontinence | genitourinary | SMD | 0.040 | 0.74 (0.64 - 0.85) | 282 | 15860 |
| 789 | Nausea and vomiting | symptoms | SMI | 0.040 | 0.93 (0.89 - 0.96) | 4547 | 13099 |
| 496.2 | Chronic bronchitis | respiratory | SMD | 0.040 | 0.63 (0.51 - 0.79) | 122 | 16322 |
| 394 | Rheumatic disease of the heart valves | circulatory system | SMD | 0.042 | 0.63 (0.51 - 0.79) | 123 | 17028 |
| 853 | Complication of colostomy or enterostomy | injuries & poisonings | SMI | 0.043 | 0.66 (0.54 - 0.80) | 102 | 16976 |
| 519 | Other diseases of respiratory system, not elsewhere classified | respiratory | SMI | 0.043 | 0.76 (0.67 - 0.87) | 262 | 17375 |
| 577.2 | Chronic pancreatitis | digestive | SMI | 0.045 | 0.73 (0.63 - 0.85) | 178 | 16521 |
| 426.92 | Cardiac defibrillator in situ | circulatory system | SMI | 0.048 | 0.66 (0.55 - 0.81) | 125 | 12606 |
| 420.2 | Pericarditis | circulatory system | SMI | 0.055 | 0.71 (0.60 - 0.84) | 163 | 17109 |
| 428.1 | Congestive heart failure (CHF) NOS | circulatory system | SMI | 0.061 | 0.80 (0.72 - 0.89) | 435 | 16709 |
| 272.11 | Hypercholesterolemia | endocrine/metabolic | SMI | 0.063 | 1.30 (1.14 - 1.47) | 310 | 14762 |
| 519 | Other diseases of respiratory system, not elsewhere classified | respiratory | SMD | 0.064 | 0.74 (0.64 - 0.86) | 262 | 17368 |
| 575.7 | Other disorders of gallbladder | digestive | SMI | 0.067 | 0.73 (0.63 - 0.85) | 192 | 15847 |
| 687.1 | Rash and other nonspecific skin eruption | dermatologic | SMD | 0.068 | 0.78 (0.70 - 0.88) | 417 | 16831 |
| 338.1 | Acute pain | neurological | SMI | 0.068 | 0.88 (0.82 - 0.93) | 1143 | 15508 |
| 277 | Other disorders of metabolism | endocrine/metabolic | SMD | 0.071 | 0.66 (0.53 - 0.81) | 127 | 17303 |
| 599.2 | Retention of urine | genitourinary | SMI | 0.074 | 0.80 (0.72 - 0.89) | 440 | 15864 |
| 429 | Ill-defined descriptions and complications of heart disease | circulatory system | SMD | 0.079 | 0.64 (0.52 - 0.80) | 116 | 16703 |
| 574.2 | Calculus of bile duct | digestive | SMD | 0.083 | 0.72 (0.61 - 0.85) | 222 | 15841 |
| 289 | Other diseases of blood and blood-forming organs | hematopoietic | SMI | 0.085 | 0.73 (0.62 - 0.85) | 174 | 15447 |
| 204.2 | Myeloid leukemia | neoplasms | SMI | 0.085 | 0.75 (0.65 - 0.86) | 213 | 16684 |
| 170 | Cancer of bone and connective tissue | neoplasms | SMI | 0.10 | 0.78 (0.69 - 0.88) | 300 | 17346 |
| 550.2 | Diaphragmatic hernia | digestive | SMD | 0.10 | 0.77 (0.67 - 0.88) | 343 | 16378 |
| 300.9 | Posttraumatic stress disorder | mental disorders | SMD | 0.10 | 0.63 (0.51 - 0.80) | 106 | 14160 |
| 204.21 | Myeloid leukemia, acute | neoplasms | SMI | 0.10 | 0.74 (0.63 - 0.86) | 183 | 16684 |
| 295 | Schizophrenia and other psychotic disorders | mental disorders | SMD | 0.11 | 0.70 (0.59 - 0.84) | 184 | 14160 |
| 592.1 | Cystitis | genitourinary | SMI | 0.11 | 0.82 (0.75 - 0.91) | 544 | 15566 |
| 536.3 | Gastroparesis | digestive | SMD | 0.11 | 0.73 (0.63 - 0.86) | 238 | 16072 |
| 189 | Cancer of urinary organs (incl. kidney and bladder) | neoplasms | SMD | 0.12 | 0.82 (0.74 - 0.90) | 607 | 17032 |
| 070.2 | Viral hepatitis B | infectious diseases | SMD | 0.12 | 1.45 (1.20 - 1.74) | 191 | 16466 |
| 689 | Disorder of skin and subcutaneous tissue NOS | dermatologic | SMI | 0.13 | 0.74 (0.63 - 0.86) | 185 | 17461 |
| 960.2 | Allergy/adverse effect of penicillin | injuries & poisonings | SMI | 0.14 | 0.80 (0.72 - 0.90) | 386 | 15992 |
| 070.2 | Viral hepatitis B | infectious diseases | SMI | 0.14 | 0.74 (0.63 - 0.86) | 191 | 16473 |
| 286.2 | Encounter for long-term (current) use of anticoagulants | hematopoietic | SMI | 0.15 | 0.84 (0.77 - 0.92) | 636 | 16149 |
| 155 | Cancer of liver and intrahepatic bile duct | neoplasms | SMI | 0.15 | 0.80 (0.72 - 0.90) | 391 | 15030 |
| 198.1 | Secondary malignancy of lymph nodes | neoplasms | SMD | 0.15 | 0.82 (0.75 - 0.91) | 612 | 14136 |
| 592 | Cystitis and urethritis | genitourinary | SMI | 0.16 | 0.83 (0.75 - 0.91) | 556 | 15566 |
| 281 | Other deficiency anemia | hematopoietic | SMI | 0.16 | 0.68 (0.55 - 0.83) | 121 | 14706 |
| 080 | Postoperative infection | infectious diseases | SMI | 0.17 | 0.78 (0.68 - 0.88) | 257 | 17215 |
| 600 | Hyperplasia of prostate | genitourinary | SMI | 0.18 | 1.25 (1.11 - 1.39) | 539 | 6261 |
| 599 | Other symptoms/disorders or the urinary system | genitourinary | SMD | 0.18 | 0.88 (0.82 - 0.94) | 1478 | 15860 |
| 202.2 | Non-Hodgkins lymphoma | neoplasms | SMD | 0.19 | 0.77 (0.68 - 0.88) | 334 | 16677 |
| 110 | Dermatophytosis / Dermatomycosis | infectious diseases | SMD | 0.19 | 0.73 (0.62 - 0.86) | 216 | 16933 |
| 495.2 | Asthma with exacerbation | respiratory | SMI | 0.19 | 1.46 (1.20 - 1.78) | 103 | 16329 |
| 070.3 | Viral hepatitis C | infectious diseases | SMD | 0.20 | 0.78 (0.69 - 0.89) | 359 | 16466 |
| 870 | Open wounds of head; neck; and trunk | injuries & poisonings | SMI | 0.21 | 0.79 (0.69 - 0.89) | 276 | 17226 |
| 770 | Myalgia and myositis unspecified | symptoms | SMD | 0.21 | 0.79 (0.70 - 0.90) | 403 | 17236 |
| 136 | Other infectious and parasitic diseases | infectious diseases | SMI | 0.24 | 0.76 (0.65 - 0.88) | 203 | 17394 |
| 850 | Hemorrhage or hematoma complicating a procedure | injuries & poisonings | SMD | 0.25 | 0.69 (0.57 - 0.84) | 149 | 16969 |
| 709 | Diffuse diseases of connective tissue | dermatologic | SMI | 0.27 | 0.73 (0.62 - 0.86) | 156 | 16827 |
| 054 | Herpes simplex | infectious diseases | SMD | 0.27 | 0.68 (0.56 - 0.84) | 135 | 16466 |
| 420.2 | Pericarditis | circulatory system | SMD | 0.29 | 0.71 (0.59 - 0.85) | 163 | 17102 |
| 740.9 | Osteoarthrosis NOS | musculoskeletal | SMD | 0.32 | 0.75 (0.65 - 0.88) | 263 | 17075 |
| 512.7 | Shortness of breath | respiratory | SMI | 0.37 | 0.90 (0.85 - 0.95) | 1556 | 15037 |
| 586.4 | Stricture/obstruction of ureter | genitourinary | SMD | 0.37 | 0.71 (0.59 - 0.85) | 166 | 14818 |
| 272.13 | Mixed hyperlipidemia | endocrine/metabolic | SMI | 0.38 | 1.42 (1.17 - 1.72) | 129 | 14762 |
| 289.5 | Diseases of spleen | hematopoietic | SMI | 0.40 | 0.74 (0.63 - 0.87) | 163 | 15447 |
| 531 | Peptic ulcer (excl. esophageal) | digestive | SMD | 0.41 | 0.80 (0.71 - 0.91) | 410 | 17229 |
| 575.1 | Cholangitis | digestive | SMD | 0.42 | 0.77 (0.67 - 0.89) | 291 | 15841 |
| 695 | Erythematous conditions | dermatologic | SMD | 0.43 | 0.79 (0.69 - 0.90) | 349 | 16949 |
| 558 | Noninfectious gastroenteritis | digestive | SMD | 0.49 | 0.85 (0.78 - 0.93) | 802 | 11799 |
| 696.4 | Psoriasis | dermatologic | SMI | 0.50 | 1.43 (1.17 - 1.75) | 106 | 16765 |
| 726 | Peripheral enthesopathies and allied syndromes | musculoskeletal | SMI | 0.51 | 1.29 (1.12 - 1.49) | 218 | 16973 |
| 540.11 | Acute appendicitis | digestive | SMI | 0.52 | 1.17 (1.07 - 1.28) | 525 | 17046 |
| 180 | Cervical cancer and dysplasia | neoplasms | SMI | 0.54 | 0.73 (0.62 - 0.87) | 148 | 8566 |
| 979 | Adverse drug events and drug allergies | injuries & poisonings | SMI | 0.55 | 0.70 (0.58 - 0.86) | 112 | 15992 |
| 261 | Vitamin deficiency | endocrine/metabolic | SMD | 0.58 | 0.82 (0.74 - 0.92) | 517 | 15668 |
| 766 | Neuralgia, neuritis, and radiculitis NOS | symptoms | SMD | 0.59 | 0.72 (0.60 - 0.87) | 162 | 17093 |
| 716.9 | Arthropathy NOS | musculoskeletal | SMD | 0.60 | 0.66 (0.53 - 0.84) | 106 | 17063 |
| 788 | Syncope and collapse | symptoms | SMI | 0.60 | 0.82 (0.74 - 0.92) | 400 | 17246 |
| 930 | Allergic reaction to food | injuries & poisonings | SMI | 0.60 | 0.72 (0.60 - 0.87) | 127 | 16750 |
| 348.9 | Other conditions of brain, NOS | neurological | SMD | 0.61 | 0.69 (0.57 - 0.85) | 139 | 16348 |
| 819 | Skull and face fracture and other intercranial injury | injuries & poisonings | SMI | 0.61 | 0.71 (0.59 - 0.86) | 106 | 17494 |
| 592.11 | Acute cystitis | genitourinary | SMI | 0.62 | 0.83 (0.75 - 0.92) | 459 | 15566 |
| 687 | Symptoms affecting skin | dermatologic | SMD | 0.66 | 0.70 (0.57 - 0.86) | 133 | 16831 |
| 180.1 | Cervical cancer | neoplasms | SMI | 0.66 | 0.72 (0.60 - 0.87) | 133 | 8566 |
| 916 | Contusion | injuries & poisonings | SMD | 0.68 | 0.78 (0.67 - 0.90) | 276 | 17363 |
| 502 | Postinflammatory pulmonary fibrosis | respiratory | SMI | 0.68 | 0.71 (0.59 - 0.86) | 128 | 15349 |
| 766 | Neuralgia, neuritis, and radiculitis NOS | symptoms | SMI | 0.72 | 0.75 (0.64 - 0.88) | 162 | 17097 |
| 751 | Genitourinary congenital anomalies | congenital anomalies | SMI | 0.75 | 0.75 (0.63 - 0.88) | 148 | 17411 |
| 573 | Other disorders of liver | digestive | SMD | 0.80 | 0.86 (0.79 - 0.94) | 830 | 13909 |
| 261 | Vitamin deficiency | endocrine/metabolic | SMI | 0.83 | 0.84 (0.77 - 0.93) | 518 | 15673 |
| 218.1 | Uterine leiomyoma | neoplasms | SMD | 0.85 | 1.26 (1.10 - 1.44) | 377 | 8963 |
| 536 | Disorders of function of stomach | digestive | SMD | 0.86 | 0.84 (0.75 - 0.93) | 553 | 16072 |
| 202.2 | Non-Hodgkins lymphoma | neoplasms | SMI | 0.88 | 0.81 (0.72 - 0.92) | 334 | 16684 |
| 853 | Complication of colostomy or enterostomy | injuries & poisonings | SMD | 0.88 | 0.67 (0.54 - 0.85) | 102 | 16969 |
| 627 | Menopausal and postmenopausal disorders | genitourinary | SMI | 0.89 | 1.25 (1.10 - 1.43) | 255 | 9274 |
| 531.2 | Gastric ulcer | digestive | SMI | 0.90 | 0.72 (0.60 - 0.87) | 125 | 17236 |
| 189.11 | Malignant neoplasm of kidney, except pelvis | neoplasms | SMD | 0.91 | 0.76 (0.64 - 0.89) | 220 | 17032 |
| 450 | Noninfectious disorders of lymphatic channels | circulatory system | SMD | 0.92 | 0.69 (0.56 - 0.86) | 119 | 17520 |
| 585.4 | Chronic kidney disease, Stage I or II | genitourinary | SMD | 0.93 | 0.66 (0.52 - 0.84) | 102 | 14818 |
| 573.4 | Acute and subacute necrosis of liver | digestive | SMI | 0.96 | 0.73 (0.61 - 0.88) | 129 | 13915 |
| 513 | Respiratory abnormalities | respiratory | SMI | 0.96 | 0.74 (0.62 - 0.88) | 149 | 17497 |
| 743.9 | Osteopenia or other disorder of bone and cartilage | musculoskeletal | SMI | 0.99 | 0.79 (0.69 - 0.91) | 265 | 16918 |
| 189.1 | Cancer of kidney and renal pelvis | neoplasms | SMD | 0.99 | 0.78 (0.67 - 0.90) | 275 | 17032 |
| 170.2 | Cancer of connective tissue | neoplasms | SMI | 1.0 | 0.80 (0.70 - 0.91) | 269 | 17346 |
| 441 | Vascular insufficiency of intestine | circulatory system | SMI | 1.0 | 0.78 (0.68 - 0.90) | 228 | 16724 |
| 733 | Other disorders of bone and cartilage | musculoskeletal | SMI | 1.0 | 0.76 (0.65 - 0.90) | 189 | 17069 |
| 686 | Other local infections of skin and subcutaneous tissue | dermatologic | SMI | 1.0 | 0.72 (0.60 - 0.88) | 114 | 16728 |
| 615 | Endometriosis | genitourinary | SMI | 1.0 | 1.28 (1.11 - 1.48) | 180 | 9622 |
| 356 | Hereditary and idiopathic peripheral neuropathy | neurological | SMD | 1.0 | 0.71 (0.58 - 0.87) | 139 | 17286 |
| 577.3 | Cyst and pseudocyst of pancreas | digestive | SMD | 1.0 | 0.75 (0.64 - 0.89) | 198 | 16514 |
| 681 | Superficial cellulitis and abscess | dermatologic | SMI | 1.0 | 0.88 (0.82 - 0.95) | 823 | 16728 |
| 218 | Benign neoplasm of uterus | neoplasms | SMD | 1.0 | 1.25 (1.09 - 1.43) | 384 | 8963 |
| 702.2 | Seborrheic keratosis | dermatologic | SMD | 1.0 | 1.35 (1.13 - 1.62) | 194 | 17313 |
| 737 | Curvature of spine | musculoskeletal | SMD | 1.0 | 0.69 (0.55 - 0.86) | 109 | 17323 |
| 289.4 | Lymphadenitis | hematopoietic | SMI | 1.0 | 0.88 (0.81 - 0.95) | 766 | 15447 |
| 696 | Psoriasis and related disorders | dermatologic | SMI | 1.0 | 1.38 (1.13 - 1.67) | 112 | 16765 |
| 350 | Abnormal movement | neurological | SMI | 1.0 | 0.83 (0.74 - 0.93) | 371 | 17248 |
| 255 | Disorders of adrenal glands | endocrine/metabolic | SMI | 1.0 | 0.80 (0.71 - 0.92) | 260 | 16939 |
| 289 | Other diseases of blood and blood-forming organs | hematopoietic | SMD | 1.0 | 0.74 (0.62 - 0.89) | 173 | 15441 |
| 350.1 | Abnormal involuntary movements | neurological | SMD | 1.0 | 0.74 (0.61 - 0.89) | 161 | 17242 |
| 965.1 | Opiates and related narcotics causing adverse effects in therapeutic use | injuries & poisonings | SMD | 1.0 | 0.76 (0.64 - 0.90) | 210 | 15985 |
| 716 | Other arthropathies | musculoskeletal | SMD | 1.0 | 0.70 (0.56 - 0.87) | 118 | 17063 |
| 433.8 | Late effects of cerebrovascular disease | circulatory system | SMI | 1.0 | 0.73 (0.60 - 0.88) | 132 | 16761 |
| 578.1 | Hematemesis | digestive | SMI | 1.0 | 0.74 (0.62 - 0.89) | 126 | 15485 |
| 350 | Abnormal movement | neurological | SMD | 1.0 | 0.81 (0.72 - 0.93) | 371 | 17242 |
| 519.9 | Symptoms involving respiratory system and other chest symptoms | respiratory | SMI | 1.0 | 0.76 (0.64 - 0.90) | 150 | 17375 |
| 184.1 | Malignant neoplasm of ovary and other uterine adnexa | neoplasms | SMI | 1.0 | 0.82 (0.73 - 0.93) | 344 | 8833 |
| 196 | Radiotherapy | neoplasms | SMI | 1.0 | 0.84 (0.75 - 0.94) | 381 | 14143 |
| 764 | Sciatica | symptoms | SMI | 1.0 | 1.24 (1.08 - 1.42) | 243 | 17097 |
| 760 | Back pain | symptoms | SMD | 1.0 | 0.90 (0.84 - 0.96) | 1497 | 16142 |
| 745 | Pain in joint | musculoskeletal | SMD | 1.0 | 0.90 (0.84 - 0.96) | 1533 | 16106 |
| 426.91 | Cardiac pacemaker in situ | circulatory system | SMI | 1.0 | 0.76 (0.64 - 0.90) | 211 | 12606 |
| 619 | Noninflammatory female genital disorders | genitourinary | SMI | 1.0 | 1.15 (1.05 - 1.26) | 553 | 9601 |
| 416 | Cardiomegaly | circulatory system | SMI | 1.0 | 0.85 (0.77 - 0.94) | 485 | 16494 |
| 540.1 | Appendicitis | digestive | SMI | 1.0 | 1.14 (1.05 - 1.24) | 567 | 17046 |
| 250.41 | Impaired fasting glucose | endocrine/metabolic | SMI | 1.0 | 1.33 (1.11 - 1.60) | 143 | 14753 |
| 159.3 | Malignant neoplasm of gallbladder and extrahepatic bile ducts | neoplasms | SMI | 1.0 | 0.76 (0.64 - 0.91) | 168 | 15030 |
| 208 | Benign neoplasm of colon | neoplasms | SMI | 1.0 | 1.14 (1.05 - 1.24) | 676 | 16075 |
| 695.42 | Systemic lupus erythematosus | dermatologic | SMI | 1.0 | 0.73 (0.60 - 0.90) | 104 | 16911 |
| 594.3 | Calculus of ureter | genitourinary | SMI | 1.0 | 1.24 (1.08 - 1.42) | 227 | 16294 |
| 440 | Atherosclerosis | circulatory system | SMD | 1.0 | 0.73 (0.60 - 0.90) | 145 | 16717 |
| 578.2 | Blood in stool | digestive | SMI | 1.0 | 0.86 (0.79 - 0.95) | 519 | 15485 |
| 249 | Secondary diabetes mellitus | endocrine/metabolic | SMI | 1.0 | 0.78 (0.66 - 0.92) | 164 | 14753 |
| 626 | Disorders of menstruation and other abnormal bleeding from female genital tract | genitourinary | SMI | 1.0 | 1.18 (1.06 - 1.32) | 334 | 9274 |
| 695.4 | Lupus (localized and systemic) | dermatologic | SMI | 1.0 | 0.75 (0.62 - 0.90) | 115 | 16911 |
| 560.4 | Other intestinal obstruction | digestive | SMD | 1.0 | 0.90 (0.83 - 0.96) | 1300 | 11799 |
| 184.11 | Malignant neoplasm of ovary | neoplasms | SMI | 1.0 | 0.83 (0.73 - 0.94) | 324 | 8833 |
| 800 | Fracture of lower limb | injuries & poisonings | SMD | 1.0 | 0.78 (0.66 - 0.92) | 206 | 16753 |
| 605 | Erectile dysfunction [ED] | genitourinary | SMI | 1.0 | 1.31 (1.10 - 1.57) | 147 | 6634 |
| 763 | Thoracic or lumbosacral neuritis or radiculitis, unspecified | symptoms | SMI | 1.0 | 1.28 (1.08 - 1.50) | 173 | 17097 |
| 473 | Diseases of the larynx and vocal cords | respiratory | SMI | 1.0 | 0.79 (0.67 - 0.92) | 183 | 16763 |
| 427.12 | Paroxysmal ventricular tachycardia | circulatory system | SMI | 1.0 | 0.72 (0.57 - 0.90) | 101 | 12606 |
| 338 | Pain | neurological | SMI | 1.0 | 0.93 (0.89 - 0.98) | 2138 | 15508 |
| 182 | Malignant neoplasm of uterus | neoplasms | SMI | 1.0 | 1.21 (1.07 - 1.38) | 268 | 8582 |
| 442 | Other aneurysm | circulatory system | SMD | 1.0 | 0.80 (0.69 - 0.93) | 281 | 16717 |
| 540 | Appendiceal conditions | digestive | SMI | 1.0 | 1.13 (1.04 - 1.23) | 600 | 17046 |
| 199 | Neoplasm of uncertain behavior | neoplasms | SMI | 1.0 | 0.86 (0.78 - 0.95) | 459 | 14143 |
| 514.2 | Solitary pulmonary nodule | respiratory | SMI | 1.0 | 0.85 (0.75 - 0.95) | 385 | 14750 |
| 153 | Colorectal cancer | neoplasms | SMD | 1.0 | 0.89 (0.82 - 0.96) | 1002 | 14710 |
| 250.24 | Type 2 diabetes with neurological manifestations | endocrine/metabolic | SMI | 1.0 | 1.28 (1.08 - 1.52) | 162 | 14753 |
| 198.5 | Secondary malignancy of brain/spine | neoplasms | SMD | 1.0 | 0.77 (0.65 - 0.92) | 180 | 14136 |
| 395 | Heart valve disorders | circulatory system | SMD | 1.0 | 0.84 (0.75 - 0.95) | 466 | 17028 |
| 269 | Proteinuria | endocrine/metabolic | SMD | 1.0 | 0.72 (0.57 - 0.91) | 103 | 17321 |
| 395.2 | Nonrheumatic aortic valve disorders | circulatory system | SMD | 1.0 | 0.77 (0.64 - 0.93) | 198 | 17028 |
| 327.4 | Insomnia | neurological | SMD | 1.0 | 0.87 (0.78 - 0.96) | 617 | 15923 |
| 550.1 | Inguinal hernia | digestive | SMI | 1.0 | 1.25 (1.07 - 1.47) | 203 | 16384 |
| 720 | Spinal stenosis | musculoskeletal | SMD | 1.0 | 0.81 (0.69 - 0.94) | 272 | 16752 |
| 292.1 | Aphasia/speech disturbance | mental disorders | SMI | 1.0 | 0.76 (0.63 - 0.92) | 139 | 16104 |
| 159.3 | Malignant neoplasm of gallbladder and extrahepatic bile ducts | neoplasms | SMD | 1.0 | 0.77 (0.64 - 0.93) | 168 | 15023 |
| 110.1 | Dermatophytosis | infectious diseases | SMD | 1.0 | 0.78 (0.66 - 0.93) | 194 | 16933 |
| 741 | Symptoms and disorders of the joints | musculoskeletal | SMD | 1.0 | 0.78 (0.65 - 0.93) | 182 | 17434 |
| 174.11 | Malignant neoplasm of female breast | neoplasms | SMI | 1.0 | 0.87 (0.79 - 0.96) | 548 | 8867 |
| 614.5 | Inflammatory disease of cervix, vagina, and vulva | genitourinary | SMI | 1.0 | 1.28 (1.07 - 1.53) | 123 | 9622 |
| 317.1 | Alcoholism | mental disorders | SMI | 1.0 | 0.87 (0.79 - 0.96) | 462 | 15504 |
| 771.1 | Swelling of limb | symptoms | SMI | 1.0 | 0.84 (0.74 - 0.95) | 308 | 17144 |
| 333 | Extrapyramidal disease and abnormal movement disorders | neurological | SMD | 1.0 | 0.74 (0.60 - 0.92) | 126 | 16348 |
| 348.2 | Cerebral edema and compression of brain | neurological | SMI | 1.0 | 0.76 (0.63 - 0.93) | 112 | 16355 |
| 535.2 | Atrophic gastritis | digestive | SMI | 1.0 | 1.21 (1.05 - 1.39) | 223 | 16078 |
| 153.2 | Colon cancer | neoplasms | SMD | 1.0 | 0.88 (0.80 - 0.96) | 695 | 14710 |
| 706 | Diseases of sebaceous glands | dermatologic | SMI | 1.0 | 0.82 (0.71 - 0.95) | 198 | 17313 |
| 564.9 | Personal history of diseases of digestive system | digestive | SMD | 1.0 | 0.78 (0.65 - 0.93) | 174 | 11799 |
| 253 | Disorders of the pituitary gland and its hypothalamic control | endocrine/metabolic | SMI | 1.0 | 0.75 (0.61 - 0.93) | 103 | 16939 |
| 054 | Herpes simplex | infectious diseases | SMI | 1.0 | 0.78 (0.66 - 0.94) | 135 | 16473 |
| 281 | Other deficiency anemia | hematopoietic | SMD | 1.0 | 0.74 (0.59 - 0.93) | 121 | 14700 |
| 614 | Inflammatory diseases of female pelvic organs | genitourinary | SMI | 1.0 | 1.15 (1.04 - 1.27) | 402 | 9622 |
| 574.11 | Cholelithiasis with acute cholecystitis | digestive | SMD | 1.0 | 0.74 (0.60 - 0.93) | 116 | 15841 |
| 368 | Visual disturbances | sense organs | SMD | 1.0 | 0.81 (0.69 - 0.95) | 228 | 17382 |
| 537 | Other disorders of stomach and duodenum | digestive | SMD | 1.0 | 0.88 (0.80 - 0.97) | 666 | 16072 |
| 180.1 | Cervical cancer | neoplasms | SMD | 1.0 | 0.76 (0.61 - 0.93) | 133 | 8561 |
| 261.4 | Vitamin D deficiency | endocrine/metabolic | SMD | 1.0 | 0.84 (0.74 - 0.96) | 359 | 15668 |
| 244.2 | Acquired hypothyroidism | endocrine/metabolic | SMD | 1.0 | 0.73 (0.58 - 0.93) | 108 | 16096 |
| 433 | Cerebrovascular disease | circulatory system | SMI | 1.0 | 0.89 (0.82 - 0.97) | 760 | 16761 |
| 611 | Abnormal findings on mammogram or breast exam | genitourinary | SMD | 1.0 | 1.28 (1.06 - 1.55) | 185 | 17356 |
| 961.1 | Poisoning/allergy of sulfonamides | injuries & poisonings | SMD | 1.0 | 0.82 (0.70 - 0.95) | 255 | 15985 |
| 261.4 | Vitamin D deficiency | endocrine/metabolic | SMI | 1.0 | 0.86 (0.76 - 0.96) | 359 | 15673 |
| 465.2 | Acute pharyngitis | respiratory | SMD | 1.0 | 1.33 (1.07 - 1.66) | 137 | 17227 |
| 442 | Other aneurysm | circulatory system | SMI | 1.0 | 0.84 (0.73 - 0.96) | 281 | 16724 |
| 317 | Alcohol-related disorders | mental disorders | SMI | 1.0 | 0.88 (0.81 - 0.97) | 513 | 15504 |
| 807 | Fracture of ribs | injuries & poisonings | SMD | 1.0 | 1.31 (1.07 - 1.61) | 154 | 16753 |
| 153.3 | Malignant neoplasm of rectum, rectosigmoid junction, and anus | neoplasms | SMD | 1.0 | 0.87 (0.78 - 0.97) | 506 | 14710 |
| 628 | Ovarian cyst | genitourinary | SMI | 1.0 | 1.14 (1.03 - 1.27) | 384 | 9274 |
| 626.1 | Irregular menstrual cycle/bleeding | genitourinary | SMI | 1.0 | 1.21 (1.04 - 1.39) | 188 | 9274 |
| 184 | Cancer of other female genital organs | neoplasms | SMI | 1.0 | 0.86 (0.77 - 0.97) | 382 | 8833 |
| 317.11 | Alcoholic liver damage | mental disorders | SMI | 1.0 | 0.84 (0.73 - 0.96) | 225 | 15504 |
| 442.1 | Aortic aneurysm | circulatory system | SMI | 1.0 | 0.79 (0.65 - 0.95) | 159 | 16724 |
| 340 | Migraine | neurological | SMI | 1.0 | 1.14 (1.03 - 1.26) | 403 | 16892 |
| 687.1 | Rash and other nonspecific skin eruption | dermatologic | SMI | 1.0 | 0.88 (0.79 - 0.97) | 417 | 16836 |
| 260.6 | Anorexia | endocrine/metabolic | SMD | 1.0 | 0.83 (0.72 - 0.96) | 302 | 15668 |
| 208 | Benign neoplasm of colon | neoplasms | SMD | 1.0 | 1.13 (1.03 - 1.25) | 676 | 16068 |
| 216 | Benign neoplasm of skin | neoplasms | SMD | 1.0 | 1.26 (1.05 - 1.51) | 197 | 17322 |
| 476 | Allergic rhinitis | respiratory | SMD | 1.0 | 1.22 (1.04 - 1.44) | 250 | 16757 |
| 619.1 | Noninflammatory disorders of ovary, fallopian tube, and broad ligament | genitourinary | SMI | 1.0 | 1.19 (1.03 - 1.36) | 221 | 9601 |
| 327.7 | Sleep related movement disorders | neurological | SMI | 1.0 | 1.31 (1.05 - 1.62) | 104 | 15928 |
| 580 | Nephritis; nephrosis; renal sclerosis | genitourinary | SMI | 1.0 | 0.80 (0.67 - 0.96) | 139 | 14825 |
| 159.2 | Malignant neoplasm of small intestine, including duodenum | neoplasms | SMI | 1.0 | 0.78 (0.64 - 0.95) | 117 | 15030 |
| 174.1 | Breast cancer [female] | neoplasms | SMI | 1.0 | 0.89 (0.81 - 0.98) | 561 | 8867 |
| 555 | Inflammatory bowel disease and other gastroenteritis and colitis | digestive | SMD | 1.0 | 0.88 (0.80 - 0.98) | 650 | 11799 |
| 196 | Radiotherapy | neoplasms | SMD | 1.0 | 0.86 (0.76 - 0.97) | 381 | 14136 |
| 199 | Neoplasm of uncertain behavior | neoplasms | SMD | 1.0 | 0.87 (0.77 - 0.97) | 459 | 14136 |
| 626.1 | Irregular menstrual cycle/bleeding | genitourinary | SMD | 1.0 | 1.28 (1.05 - 1.56) | 188 | 9271 |
| 840 | Sprains and strains | injuries & poisonings | SMI | 1.0 | 1.26 (1.04 - 1.51) | 120 | 17452 |
| 627 | Menopausal and postmenopausal disorders | genitourinary | SMD | 1.0 | 1.21 (1.04 - 1.42) | 254 | 9271 |
| 531.4 | Peptic ulcer, site unspecified | digestive | SMI | 1.0 | 0.81 (0.68 - 0.97) | 154 | 17236 |
| 496 | Chronic airway obstruction | respiratory | SMI | 1.0 | 0.89 (0.81 - 0.98) | 577 | 16329 |
| 751.2 | Congenital anomalies of urinary system | congenital anomalies | SMI | 1.0 | 0.79 (0.65 - 0.96) | 113 | 17411 |
| 599.2 | Retention of urine | genitourinary | SMD | 1.0 | 0.87 (0.77 - 0.98) | 440 | 15860 |
| 747 | Cardiac and circulatory congenital anomalies | congenital anomalies | SMD | 1.0 | 0.77 (0.63 - 0.96) | 124 | 17515 |
| 441 | Vascular insufficiency of intestine | circulatory system | SMD | 1.0 | 0.82 (0.70 - 0.97) | 228 | 16717 |
| 702 | Degenerative skin conditions and other dermatoses | dermatologic | SMD | 1.0 | 1.18 (1.03 - 1.36) | 326 | 17100 |
| 949 | Allergies, other | injuries & poisonings | SMD | 1.0 | 0.84 (0.73 - 0.97) | 287 | 16743 |
| 790.6 | Other abnormal blood chemistry | symptoms | SMI | 1.0 | 0.91 (0.85 - 0.99) | 829 | 16697 |
| 555.21 | Ulcerative colitis (chronic) | digestive | SMD | 1.0 | 0.79 (0.64 - 0.97) | 135 | 11799 |
| 386.9 | Dizziness and giddiness (Light-headedness and vertigo) | sense organs | SMD | 1.0 | 0.89 (0.81 - 0.98) | 634 | 16965 |
| 586.4 | Stricture/obstruction of ureter | genitourinary | SMI | 1.0 | 0.82 (0.69 - 0.97) | 166 | 14825 |
| 426.32 | Left bundle branch block | circulatory system | SMI | 1.0 | 0.83 (0.71 - 0.97) | 239 | 12606 |
| 599.9 | Other abnormality of urination | genitourinary | SMI | 1.0 | 1.19 (1.02 - 1.39) | 204 | 15864 |
| 290.1 | Dementias | mental disorders | SMD | 1.0 | 0.83 (0.71 - 0.97) | 312 | 16099 |
| 720.1 | Spinal stenosis of lumbar region | musculoskeletal | SMD | 1.0 | 0.80 (0.67 - 0.97) | 176 | 16752 |
| 496.1 | Emphysema | respiratory | SMD | 1.0 | 0.77 (0.62 - 0.97) | 121 | 16322 |
| 300.11 | Generalized anxiety disorder | mental disorders | SMI | 1.0 | 0.85 (0.74 - 0.98) | 239 | 14166 |
| 450 | Noninfectious disorders of lymphatic channels | circulatory system | SMI | 1.0 | 0.80 (0.65 - 0.97) | 119 | 17527 |
| 189.2 | Cancer of bladder | neoplasms | SMD | 1.0 | 0.85 (0.74 - 0.98) | 333 | 17032 |
| 242 | Thyrotoxicosis with or without goiter | endocrine/metabolic | SMI | 1.0 | 0.78 (0.63 - 0.97) | 103 | 16103 |
| 242 | Thyrotoxicosis with or without goiter | endocrine/metabolic | SMD | 1.0 | 0.76 (0.60 - 0.97) | 103 | 16096 |
| 174 | Breast cancer | neoplasms | SMI | 1.0 | 0.90 (0.82 - 0.99) | 570 | 16350 |
| 496.1 | Emphysema | respiratory | SMI | 1.0 | 0.79 (0.64 - 0.97) | 121 | 16329 |
| 528 | Diseases of the oral soft tissues, excluding lesions specific for gingiva and tongue | digestive | SMD | 1.0 | 0.81 (0.67 - 0.97) | 161 | 17378 |
| 172.1 | Melanomas of skin, dx or hx | neoplasms | SMI | 1.0 | 0.79 (0.64 - 0.97) | 112 | 17160 |
| 695.42 | Systemic lupus erythematosus | dermatologic | SMD | 1.0 | 0.76 (0.60 - 0.97) | 104 | 16904 |
| 350.1 | Abnormal involuntary movements | neurological | SMI | 1.0 | 0.82 (0.70 - 0.98) | 161 | 17248 |
| 070 | Viral hepatitis | infectious diseases | SMI | 1.0 | 0.90 (0.82 - 0.99) | 570 | 16473 |
| 211 | Benign neoplasm of other parts of digestive system | neoplasms | SMD | 1.0 | 0.81 (0.68 - 0.98) | 168 | 15134 |
| 531.2 | Gastric ulcer | digestive | SMD | 1.0 | 0.78 (0.63 - 0.97) | 125 | 17229 |
| 512.2 | Painful respiration | respiratory | SMD | 1.0 | 0.81 (0.68 - 0.98) | 175 | 15031 |
| 250.22 | Type 2 diabetes with renal manifestations | endocrine/metabolic | SMI | 1.0 | 1.20 (1.02 - 1.41) | 195 | 14753 |
| 189.21 | Malignant neoplasm of bladder | neoplasms | SMD | 1.0 | 0.85 (0.74 - 0.98) | 321 | 17032 |
| 180 | Cervical cancer and dysplasia | neoplasms | SMD | 1.0 | 0.80 (0.65 - 0.98) | 148 | 8561 |
| 574.12 | Cholelithiasis with other cholecystitis | digestive | SMD | 1.0 | 0.81 (0.67 - 0.98) | 166 | 15841 |
| 614.3 | Pelvic inflammatory disease (PID) | genitourinary | SMD | 1.0 | 0.83 (0.70 - 0.98) | 220 | 9617 |
| 426.2 | Atrioventricular [AV] block | circulatory system | SMI | 1.0 | 0.82 (0.69 - 0.98) | 203 | 12606 |
| 261.2 | Vitamin B-complex deficiencies | endocrine/metabolic | SMI | 1.0 | 0.82 (0.69 - 0.98) | 166 | 15673 |
| 574.1 | Cholelithiasis | digestive | SMI | 1.0 | 1.09 (1.01 - 1.18) | 784 | 15847 |
| 351 | Other peripheral nerve disorders | neurological | SMD | 1.0 | 0.84 (0.73 - 0.98) | 254 | 17138 |
| 155 | Cancer of liver and intrahepatic bile duct | neoplasms | SMD | 1.0 | 0.87 (0.77 - 0.99) | 391 | 15023 |
| 418 | Nonspecific chest pain | circulatory system | SMI | 1.0 | 1.07 (1.01 - 1.13) | 1425 | 16212 |
| 550.1 | Inguinal hernia | digestive | SMD | 1.0 | 1.22 (1.02 - 1.46) | 203 | 16378 |
| 979 | Adverse drug events and drug allergies | injuries & poisonings | SMD | 1.0 | 0.78 (0.62 - 0.98) | 112 | 15985 |
| 695 | Erythematous conditions | dermatologic | SMI | 1.0 | 0.88 (0.79 - 0.99) | 349 | 16956 |
| 296.1 | Bipolar | mental disorders | SMI | 1.0 | 1.15 (1.01 - 1.31) | 249 | 14166 |
| 571.81 | Portal hypertension | digestive | SMI | 1.0 | 0.88 (0.78 - 0.99) | 319 | 13915 |
| 574.2 | Calculus of bile duct | digestive | SMI | 1.0 | 0.85 (0.74 - 0.99) | 222 | 15847 |
| 550.4 | Umbilical hernia | digestive | SMD | 1.0 | 0.83 (0.69 - 0.99) | 189 | 16378 |
| 216.1 | Screening for malignant neoplasms of the skin | neoplasms | SMD | 1.0 | 1.28 (1.02 - 1.60) | 125 | 17322 |
| 803 | Fracture of upper limb | injuries & poisonings | SMD | 1.0 | 0.81 (0.67 - 0.99) | 156 | 16753 |
| 473 | Diseases of the larynx and vocal cords | respiratory | SMD | 1.0 | 0.83 (0.69 - 0.99) | 182 | 16757 |
| 411.8 | Other chronic ischemic heart disease, unspecified | circulatory system | SMI | 1.0 | 0.79 (0.63 - 0.99) | 103 | 16271 |
| 939 | Atopic/contact dermatitis due to other or unspecified | dermatologic | SMI | 1.0 | 0.89 (0.81 - 0.99) | 416 | 16750 |
| 594 | Urinary calculus | genitourinary | SMD | 1.0 | 1.10 (1.00 - 1.19) | 882 | 16288 |
| 250.4 | Abnormal glucose | endocrine/metabolic | SMI | 1.0 | 1.10 (1.00 - 1.20) | 667 | 14753 |
| 368 | Visual disturbances | sense organs | SMI | 1.0 | 0.87 (0.75 - 0.99) | 228 | 17389 |
| 442.1 | Aortic aneurysm | circulatory system | SMD | 1.0 | 0.81 (0.67 - 0.99) | 159 | 16717 |
| 578.8 | Hemorrhage of rectum and anus | digestive | SMD | 1.0 | 1.17 (1.01 - 1.37) | 277 | 15478 |
| 155.1 | Malignant neoplasm of liver, primary | neoplasms | SMI | 1.0 | 0.87 (0.75 - 1.00) | 254 | 15030 |
| 714 | Rheumatoid arthritis and other inflammatory polyarthropathies | musculoskeletal | SMI | 1.0 | 0.86 (0.74 - 1.00) | 213 | 17191 |
| 796 | Elevated prostate specific antigen [PSA] | genitourinary | SMD | 1.0 | 1.29 (1.01 - 1.65) | 108 | 6259 |
| 721 | Spondylosis and allied disorders | musculoskeletal | SMD | 1.0 | 0.88 (0.77 - 1.00) | 369 | 16752 |
| 585.33 | Chronic Kidney Disease, Stage III | genitourinary | SMI | 1.0 | 0.87 (0.76 - 1.00) | 300 | 14825 |
| 569.2 | Gastrointestinal complications | digestive | SMI | 1.0 | 0.89 (0.79 - 1.00) | 310 | 14836 |
| 357 | Inflammatory and toxic neuropathy | neurological | SMI | 1.0 | 0.85 (0.72 - 1.00) | 179 | 17292 |
| 295 | Schizophrenia and other psychotic disorders | mental disorders | SMI | 1.0 | 0.85 (0.73 - 1.00) | 184 | 14166 |
| 626 | Disorders of menstruation and other abnormal bleeding from female genital tract | genitourinary | SMD | 1.0 | 1.16 (1.00 - 1.35) | 334 | 9271 |
| 608 | Other disorders of male genital organs | genitourinary | SMI | 1.0 | 1.19 (1.00 - 1.43) | 136 | 6634 |
| 327.7 | Sleep related movement disorders | neurological | SMD | 1.0 | 0.79 (0.62 - 1.00) | 104 | 15923 |
| 535.9 | Gastritis and duodenitis, NOS | digestive | SMD | 1.0 | 1.21 (1.00 - 1.46) | 182 | 16072 |
| 721.1 | Spondylosis without myelopathy | musculoskeletal | SMD | 1.0 | 0.87 (0.76 - 1.00) | 333 | 16752 |
| 579 | Other symptoms involving abdomen and pelvis | digestive | SMI | 1.0 | 0.93 (0.87 - 1.00) | 932 | 15485 |
| 555.2 | Ulcerative colitis | digestive | SMD | 1.0 | 0.87 (0.76 - 1.00) | 321 | 11799 |
| 965 | Poisoning by analgesics, antipyretics, and antirheumatics | injuries & poisonings | SMI | 1.0 | 0.85 (0.72 - 1.00) | 164 | 15992 |
| 189.2 | Cancer of bladder | neoplasms | SMI | 1.0 | 1.13 (1.00 - 1.29) | 333 | 17039 |
| 110.1 | Dermatophytosis | infectious diseases | SMI | 1.0 | 1.17 (0.99 - 1.37) | 194 | 16940 |
| 379 | Other disorders of eye | sense organs | SMI | 1.0 | 1.14 (0.99 - 1.32) | 248 | 17164 |
| 573.3 | Hepatomegaly | digestive | SMI | 1.0 | 0.87 (0.75 - 1.01) | 201 | 13915 |
| 722 | Intervertebral disc disorders | musculoskeletal | SMD | 1.0 | 0.88 (0.77 - 1.01) | 336 | 16752 |
| 695.4 | Lupus (localized and systemic) | dermatologic | SMD | 1.0 | 0.80 (0.64 - 1.01) | 115 | 16904 |
| 389 | Hearing loss | sense organs | SMI | 1.0 | 0.89 (0.80 - 1.00) | 386 | 17258 |
| 743.11 | Osteoporosis NOS | musculoskeletal | SMD | 1.0 | 1.13 (0.99 - 1.29) | 383 | 16912 |
| 379.2 | Disorders of vitreous body | sense organs | SMI | 1.0 | 1.16 (0.99 - 1.37) | 190 | 17164 |
| 599.5 | Frequency of urination and polyuria | genitourinary | SMI | 1.0 | 1.10 (0.99 - 1.23) | 423 | 15864 |
| 727 | Other disorders of synovium, tendon, and bursa | musculoskeletal | SMI | 1.0 | 1.16 (0.99 - 1.37) | 171 | 16973 |
| 761 | Cervicalgia | symptoms | SMD | 1.0 | 1.13 (0.99 - 1.29) | 381 | 17258 |
| 571.51 | Cirrhosis of liver without mention of alcohol | digestive | SMI | 1.0 | 0.91 (0.83 - 1.01) | 534 | 13915 |
| 586 | Other disorders of the kidney and ureters | genitourinary | SMI | 1.0 | 0.93 (0.86 - 1.01) | 857 | 14825 |
| 184.11 | Malignant neoplasm of ovary | neoplasms | SMD | 1.0 | 0.88 (0.77 - 1.01) | 324 | 8828 |
| 362.2 | Degeneration of macula and posterior pole of retina | sense organs | SMD | 1.0 | 1.17 (0.99 - 1.39) | 223 | 16998 |
| 395 | Heart valve disorders | circulatory system | SMI | 1.0 | 0.90 (0.81 - 1.01) | 466 | 17035 |
| 287.31 | Primary thrombocytopenia | hematopoietic | SMI | 1.0 | 0.83 (0.68 - 1.02) | 110 | 16149 |
| 293.1 | Swelling, mass, or lump in head and neck [Space-occupying lesion, intracranial NOS] | mental disorders | SMD | 1.0 | 0.82 (0.67 - 1.02) | 131 | 17499 |
| 339 | Other headache syndromes | neurological | SMD | 1.0 | 0.89 (0.79 - 1.01) | 407 | 16885 |
| 473.4 | Voice disturbance | respiratory | SMI | 1.0 | 0.84 (0.69 - 1.02) | 129 | 16763 |
| 564.1 | Irritable Bowel Syndrome | digestive | SMI | 1.0 | 0.87 (0.75 - 1.01) | 204 | 11805 |
| 740.9 | Osteoarthrosis NOS | musculoskeletal | SMI | 1.0 | 1.13 (0.99 - 1.30) | 263 | 17082 |
| 535 | Gastritis and duodenitis | digestive | SMD | 1.0 | 1.10 (0.99 - 1.22) | 608 | 16072 |
| 800.1 | Fracture of neck of femur | injuries & poisonings | SMI | 1.0 | 0.83 (0.67 - 1.02) | 114 | 16757 |
| 401.3 | Other hypertensive complications | circulatory system | SMI | 1.0 | 0.85 (0.71 - 1.02) | 139 | 13223 |
| 536.8 | Dyspepsia and other specified disorders of function of stomach | digestive | SMD | 1.0 | 1.16 (0.98 - 1.38) | 218 | 16072 |
| 172.1 | Melanomas of skin, dx or hx | neoplasms | SMD | 1.0 | 0.81 (0.65 - 1.03) | 112 | 17153 |
| 279.7 | Other immunological findings | endocrine/metabolic | SMI | 1.0 | 0.83 (0.68 - 1.02) | 102 | 17084 |
| 172 | Skin cancer | neoplasms | SMI | 1.0 | 0.90 (0.80 - 1.01) | 388 | 17160 |
| 465 | Acute upper respiratory infections of multiple or unspecified sites | respiratory | SMI | 1.0 | 1.11 (0.98 - 1.24) | 319 | 17234 |
| 367 | Disorders of refraction and accommodation; blindness and low vision | sense organs | SMD | 1.0 | 0.87 (0.75 - 1.02) | 251 | 17388 |
| 555.1 | Regional enteritis | digestive | SMD | 1.0 | 0.89 (0.78 - 1.02) | 351 | 11799 |
| 464 | Acute sinusitis | respiratory | SMI | 1.0 | 1.19 (0.97 - 1.44) | 109 | 17234 |
| 411.2 | Myocardial infarction | circulatory system | SMI | 1.0 | 0.91 (0.81 - 1.02) | 423 | 16271 |
| 512.2 | Painful respiration | respiratory | SMI | 1.0 | 0.87 (0.74 - 1.02) | 176 | 15037 |
| 726.1 | Enthesopathy | musculoskeletal | SMI | 1.0 | 1.19 (0.97 - 1.45) | 112 | 16973 |
| 949 | Allergies, other | injuries & poisonings | SMI | 1.0 | 0.90 (0.79 - 1.02) | 287 | 16750 |
| 706 | Diseases of sebaceous glands | dermatologic | SMD | 1.0 | 1.17 (0.98 - 1.40) | 198 | 17306 |
| 614 | Inflammatory diseases of female pelvic organs | genitourinary | SMD | 1.0 | 0.90 (0.79 - 1.02) | 402 | 9617 |
| 615 | Endometriosis | genitourinary | SMD | 1.0 | 1.18 (0.97 - 1.44) | 180 | 9617 |
| 720 | Spinal stenosis | musculoskeletal | SMI | 1.0 | 1.13 (0.98 - 1.29) | 272 | 16756 |
| 209 | Neuroendocrine tumors | neoplasms | SMI | 1.0 | 0.88 (0.75 - 1.02) | 199 | 17447 |
| 930 | Allergic reaction to food | injuries & poisonings | SMD | 1.0 | 0.83 (0.67 - 1.04) | 127 | 16743 |
| 568.1 | Peritoneal adhesions (postoperative) (postinfection) | digestive | SMI | 1.0 | 0.89 (0.78 - 1.02) | 259 | 14836 |
| 760 | Back pain | symptoms | SMI | 1.0 | 1.05 (0.99 - 1.11) | 1500 | 16146 |
| 380.4 | Impacted cerumen | sense organs | SMD | 1.0 | 1.22 (0.96 - 1.56) | 109 | 17506 |
| 722.6 | Degeneration of intervertebral disc | musculoskeletal | SMD | 1.0 | 0.86 (0.72 - 1.03) | 183 | 16752 |
| 599.9 | Other abnormality of urination | genitourinary | SMD | 1.0 | 1.16 (0.97 - 1.39) | 202 | 15860 |
| 296.2 | Depression | mental disorders | SMI | 1.0 | 0.96 (0.91 - 1.01) | 1948 | 14166 |
| 916 | Contusion | injuries & poisonings | SMI | 1.0 | 0.90 (0.79 - 1.02) | 276 | 17370 |
| 716 | Other arthropathies | musculoskeletal | SMI | 1.0 | 1.18 (0.97 - 1.43) | 118 | 17069 |
| 789.1 | Persistent vomiting | symptoms | SMD | 1.0 | 0.84 (0.68 - 1.04) | 130 | 13092 |
| 189.21 | Malignant neoplasm of bladder | neoplasms | SMI | 1.0 | 1.11 (0.98 - 1.27) | 321 | 17039 |
| 571 | Chronic liver disease and cirrhosis | digestive | SMI | 1.0 | 1.05 (0.99 - 1.10) | 1717 | 13915 |
| 395.1 | Nonrheumatic mitral valve disorders | circulatory system | SMD | 1.0 | 1.17 (0.97 - 1.41) | 184 | 17028 |
| 395.1 | Nonrheumatic mitral valve disorders | circulatory system | SMI | 1.0 | 0.87 (0.74 - 1.03) | 184 | 17035 |
| 871 | Open wounds of extremities | injuries & poisonings | SMI | 1.0 | 0.87 (0.74 - 1.03) | 153 | 17226 |
| 250.3 | Insulin pump user | endocrine/metabolic | SMI | 1.0 | 1.08 (0.98 - 1.20) | 487 | 14753 |
| 714.1 | Rheumatoid arthritis | musculoskeletal | SMI | 1.0 | 0.88 (0.75 - 1.03) | 184 | 17191 |
| 530.2 | Esophageal bleeding (varices/hemorrhage) | digestive | SMI | 1.0 | 0.89 (0.77 - 1.03) | 225 | 14903 |
| 184.1 | Malignant neoplasm of ovary and other uterine adnexa | neoplasms | SMD | 1.0 | 0.90 (0.79 - 1.03) | 344 | 8828 |
| 611.3 | Lump or mass in breast | genitourinary | SMI | 1.0 | 0.85 (0.69 - 1.04) | 104 | 17363 |
| 187 | Cancer of other male genital organs | neoplasms | SMI | 1.0 | 1.12 (0.97 - 1.30) | 189 | 6826 |
| 433.1 | Occlusion and stenosis of precerebral arteries | circulatory system | SMD | 1.0 | 0.84 (0.67 - 1.05) | 127 | 16754 |
| 411.4 | Coronary atherosclerosis | circulatory system | SMI | 1.0 | 1.06 (0.98 - 1.15) | 1024 | 16271 |
| 586.2 | Cyst of kidney, acquired | genitourinary | SMD | 1.0 | 1.21 (0.95 - 1.54) | 115 | 14818 |
| 396 | Abnormal heart sounds | circulatory system | SMI | 1.0 | 0.84 (0.68 - 1.05) | 102 | 17035 |
| 722.1 | Displacement of intervertebral disc | musculoskeletal | SMI | 1.0 | 1.17 (0.96 - 1.43) | 105 | 16756 |
| 716.9 | Arthropathy NOS | musculoskeletal | SMI | 1.0 | 1.18 (0.95 - 1.44) | 106 | 17069 |
| 565 | Anal and rectal conditions | digestive | SMD | 1.0 | 0.92 (0.83 - 1.02) | 541 | 14829 |
| 070 | Viral hepatitis | infectious diseases | SMD | 1.0 | 0.92 (0.83 - 1.02) | 570 | 16466 |
| 338.2 | Chronic pain | neurological | SMI | 1.0 | 0.95 (0.89 - 1.02) | 1089 | 15508 |
| 316 | Substance addiction and disorders | mental disorders | SMI | 1.0 | 0.93 (0.84 - 1.02) | 443 | 15504 |
| 625 | Pain and other symptoms associated with female genital organs | genitourinary | SMI | 1.0 | 1.09 (0.97 - 1.21) | 366 | 9715 |
| 257.1 | Testicular hypofunction | endocrine/metabolic | SMI | 1.0 | 1.17 (0.95 - 1.43) | 101 | 7161 |
| 681.7 | Cellulitis and abscess of trunk | dermatologic | SMI | 1.0 | 0.91 (0.81 - 1.03) | 298 | 16728 |
| 599.3 | Dysuria | genitourinary | SMI | 1.0 | 1.07 (0.98 - 1.17) | 584 | 15864 |
| 257 | Testicular dysfunction | endocrine/metabolic | SMI | 1.0 | 1.17 (0.95 - 1.43) | 102 | 7161 |
| 457 | Encounter for long-term (current) use of anticoagulants, antithrombotics, aspirin | circulatory system | SMI | 1.0 | 1.07 (0.98 - 1.17) | 706 | 15244 |
| 701 | Other hypertrophic and atrophic conditions of skin | dermatologic | SMI | 1.0 | 1.11 (0.97 - 1.26) | 240 | 17379 |
| 454 | Varicose veins | circulatory system | SMI | 1.0 | 0.87 (0.73 - 1.05) | 145 | 15244 |
| 536.8 | Dyspepsia and other specified disorders of function of stomach | digestive | SMI | 1.0 | 0.90 (0.78 - 1.04) | 218 | 16078 |
| 433.3 | Cerebral ischemia | circulatory system | SMI | 1.0 | 0.92 (0.81 - 1.03) | 377 | 16761 |
| 574.3 | Cholecystitis without cholelithiasis | digestive | SMI | 1.0 | 0.92 (0.81 - 1.03) | 334 | 15847 |
| 455 | Hemorrhoids | circulatory system | SMD | 1.0 | 1.09 (0.97 - 1.23) | 464 | 15238 |
| 579.2 | Splenomegaly | digestive | SMI | 1.0 | 0.92 (0.81 - 1.03) | 300 | 15485 |
| 720.1 | Spinal stenosis of lumbar region | musculoskeletal | SMI | 1.0 | 1.13 (0.95 - 1.35) | 176 | 16756 |
| 187.2 | Malignant neoplasm of testis | neoplasms | SMD | 1.0 | 1.15 (0.95 - 1.40) | 172 | 6824 |
| 600 | Hyperplasia of prostate | genitourinary | SMD | 1.0 | 1.09 (0.97 - 1.23) | 539 | 6259 |
| 350.2 | Abnormality of gait | neurological | SMI | 1.0 | 0.89 (0.75 - 1.05) | 182 | 17248 |
| 789.1 | Persistent vomiting | symptoms | SMI | 1.0 | 0.88 (0.74 - 1.05) | 130 | 13099 |
| 530 | Diseases of esophagus | digestive | SMI | 1.0 | 0.97 (0.92 - 1.01) | 2393 | 14903 |
| 747 | Cardiac and circulatory congenital anomalies | congenital anomalies | SMI | 1.0 | 0.88 (0.73 - 1.05) | 124 | 17522 |
| 743.1 | Osteoporosis | musculoskeletal | SMD | 1.0 | 1.10 (0.96 - 1.24) | 406 | 16912 |
| 175 | Acquired absence of breast | neoplasms | SMD | 1.0 | 0.87 (0.71 - 1.06) | 149 | 8757 |
| 296 | Mood disorders | mental disorders | SMI | 1.0 | 0.97 (0.92 - 1.01) | 2153 | 14166 |
| 355.1 | Chronic pain syndrome | neurological | SMI | 1.0 | 0.89 (0.75 - 1.05) | 149 | 17143 |
| 594.3 | Calculus of ureter | genitourinary | SMD | 1.0 | 1.12 (0.95 - 1.33) | 227 | 16288 |
| 261.2 | Vitamin B-complex deficiencies | endocrine/metabolic | SMD | 1.0 | 0.87 (0.72 - 1.06) | 165 | 15668 |
| 300.11 | Generalized anxiety disorder | mental disorders | SMD | 1.0 | 0.89 (0.76 - 1.05) | 239 | 14160 |
| 961.1 | Poisoning/allergy of sulfonamides | injuries & poisonings | SMI | 1.0 | 0.91 (0.79 - 1.04) | 255 | 15992 |
| 564.1 | Irritable Bowel Syndrome | digestive | SMD | 1.0 | 1.13 (0.95 - 1.35) | 204 | 11799 |
| 366 | Cataract | sense organs | SMI | 1.0 | 1.07 (0.97 - 1.19) | 526 | 17120 |
| 578.2 | Blood in stool | digestive | SMD | 1.0 | 0.93 (0.83 - 1.04) | 519 | 15478 |
| 694.2 | Other dyschromia | dermatologic | SMI | 1.0 | 0.88 (0.74 - 1.06) | 134 | 16956 |
| 366.2 | Senile cataract | sense organs | SMI | 1.0 | 1.10 (0.96 - 1.25) | 272 | 17120 |
| 761 | Cervicalgia | symptoms | SMI | 1.0 | 1.07 (0.96 - 1.19) | 385 | 17261 |
| 577.1 | Acute pancreatitis | digestive | SMI | 1.0 | 0.94 (0.85 - 1.03) | 495 | 16521 |
| 531.4 | Peptic ulcer, site unspecified | digestive | SMD | 1.0 | 0.88 (0.72 - 1.07) | 154 | 17229 |
| 724 | Other and unspecified disorders of back | musculoskeletal | SMD | 1.0 | 0.88 (0.72 - 1.08) | 143 | 16752 |
| 611.3 | Lump or mass in breast | genitourinary | SMD | 1.0 | 1.17 (0.92 - 1.51) | 104 | 17356 |
| 479 | Other upper respiratory disease | respiratory | SMI | 1.0 | 0.92 (0.80 - 1.05) | 254 | 16763 |
| 619.1 | Noninflammatory disorders of ovary, fallopian tube, and broad ligament | genitourinary | SMD | 1.0 | 1.12 (0.94 - 1.33) | 221 | 9596 |
| 618 | Genital prolapse | genitourinary | SMD | 1.0 | 1.16 (0.92 - 1.47) | 114 | 10035 |
| 184 | Cancer of other female genital organs | neoplasms | SMD | 1.0 | 0.92 (0.81 - 1.05) | 382 | 8828 |
| 473.4 | Voice disturbance | respiratory | SMD | 1.0 | 0.87 (0.70 - 1.08) | 128 | 16757 |
| 241.1 | Nontoxic uninodular goiter | endocrine/metabolic | SMD | 1.0 | 1.15 (0.92 - 1.45) | 122 | 16096 |
| 379.2 | Disorders of vitreous body | sense organs | SMD | 1.0 | 1.12 (0.93 - 1.35) | 190 | 17157 |
| 594.1 | Calculus of kidney | genitourinary | SMD | 1.0 | 1.06 (0.96 - 1.17) | 694 | 16288 |
| 579.8 | Nonspecific abnormal findings in stool contents | digestive | SMD | 1.0 | 1.11 (0.94 - 1.30) | 237 | 15478 |
| 698 | Pruritus and related conditions | dermatologic | SMD | 1.0 | 0.90 (0.76 - 1.07) | 213 | 17426 |
| 770 | Myalgia and myositis unspecified | symptoms | SMI | 1.0 | 1.07 (0.96 - 1.18) | 403 | 17243 |
| 356 | Hereditary and idiopathic peripheral neuropathy | neurological | SMI | 1.0 | 0.89 (0.74 - 1.08) | 140 | 17292 |
| 681.5 | Cellulitis and abscess of leg, except foot | dermatologic | SMI | 1.0 | 0.89 (0.74 - 1.08) | 131 | 16728 |
| 608 | Other disorders of male genital organs | genitourinary | SMD | 1.0 | 1.14 (0.92 - 1.42) | 136 | 6632 |
| 296.22 | Major depressive disorder | mental disorders | SMI | 1.0 | 0.96 (0.89 - 1.03) | 805 | 14166 |
| 535.2 | Atrophic gastritis | digestive | SMD | 1.0 | 1.11 (0.94 - 1.31) | 223 | 16072 |
| 401.22 | Hypertensive chronic kidney disease | circulatory system | SMI | 1.0 | 0.92 (0.80 - 1.06) | 290 | 13223 |
| 428.4 | Heart failure with preserved EF [Diastolic heart failure] | circulatory system | SMI | 1.0 | 1.12 (0.92 - 1.36) | 149 | 16709 |
| 577.3 | Cyst and pseudocyst of pancreas | digestive | SMI | 1.0 | 0.91 (0.78 - 1.06) | 198 | 16521 |
| 614.3 | Pelvic inflammatory disease (PID) | genitourinary | SMI | 1.0 | 1.08 (0.94 - 1.24) | 220 | 9622 |
| 158 | Neoplasm of unspecified nature of digestive system | neoplasms | SMI | 1.0 | 0.91 (0.78 - 1.07) | 207 | 15030 |
| 426.21 | First degree AV block | circulatory system | SMI | 1.0 | 0.88 (0.71 - 1.09) | 135 | 12606 |
| 187.2 | Malignant neoplasm of testis | neoplasms | SMI | 1.0 | 1.09 (0.94 - 1.27) | 172 | 6826 |
| 741 | Symptoms and disorders of the joints | musculoskeletal | SMI | 1.0 | 1.10 (0.93 - 1.28) | 183 | 17440 |
| 564.9 | Personal history of diseases of digestive system | digestive | SMI | 1.0 | 0.91 (0.77 - 1.07) | 174 | 11805 |
| 495.2 | Asthma with exacerbation | respiratory | SMD | 1.0 | 0.87 (0.69 - 1.11) | 103 | 16322 |
| 763 | Thoracic or lumbosacral neuritis or radiculitis, unspecified | symptoms | SMD | 1.0 | 0.90 (0.75 - 1.09) | 172 | 17093 |
| 694 | Dyschromia and Vitiligo | dermatologic | SMI | 1.0 | 0.91 (0.76 - 1.08) | 149 | 16956 |
| 729 | Other disorders of soft tissues | musculoskeletal | SMI | 1.0 | 0.92 (0.80 - 1.06) | 227 | 16973 |
| 611 | Abnormal findings on mammogram or breast exam | genitourinary | SMI | 1.0 | 0.92 (0.78 - 1.07) | 185 | 17363 |
| 395.6 | Heart valve replaced | circulatory system | SMI | 1.0 | 0.90 (0.74 - 1.09) | 138 | 17035 |
| 586.2 | Cyst of kidney, acquired | genitourinary | SMI | 1.0 | 1.12 (0.91 - 1.38) | 115 | 14825 |
| 457.3 | Encounter for long-term (current) use of aspirin | circulatory system | SMI | 1.0 | 1.05 (0.96 - 1.16) | 581 | 15244 |
| 530.11 | GERD | digestive | SMI | 1.0 | 1.03 (0.98 - 1.09) | 1746 | 14903 |
| 257.1 | Testicular hypofunction | endocrine/metabolic | SMD | 1.0 | 0.88 (0.69 - 1.12) | 101 | 7159 |
| 733 | Other disorders of bone and cartilage | musculoskeletal | SMD | 1.0 | 0.91 (0.76 - 1.09) | 189 | 17062 |
| 401.2 | Hypertensive heart and/or renal disease | circulatory system | SMI | 1.0 | 0.94 (0.84 - 1.05) | 465 | 13223 |
| 174.1 | Breast cancer [female] | neoplasms | SMD | 1.0 | 0.94 (0.85 - 1.05) | 561 | 8862 |
| 800.1 | Fracture of neck of femur | injuries & poisonings | SMD | 1.0 | 0.88 (0.70 - 1.12) | 114 | 16753 |
| 433.31 | Transient cerebral ischemia | circulatory system | SMI | 1.0 | 0.94 (0.83 - 1.06) | 358 | 16761 |
| 562.2 | Diverticulitis | digestive | SMD | 1.0 | 0.95 (0.87 - 1.05) | 754 | 11799 |
| 174.11 | Malignant neoplasm of female breast | neoplasms | SMD | 1.0 | 0.95 (0.85 - 1.05) | 548 | 8862 |
| 159 | Malignant neoplasm of other and ill-defined sites within the digestive organs and peritoneum | neoplasms | SMD | 1.0 | 0.95 (0.86 - 1.05) | 627 | 15023 |
| 175 | Acquired absence of breast | neoplasms | SMI | 1.0 | 0.91 (0.77 - 1.09) | 149 | 8762 |
| 110.11 | Dermatophytosis of nail | infectious diseases | SMI | 1.0 | 1.12 (0.90 - 1.39) | 111 | 16940 |
| 619 | Noninflammatory female genital disorders | genitourinary | SMD | 1.0 | 1.06 (0.95 - 1.18) | 553 | 9596 |
| 272.13 | Mixed hyperlipidemia | endocrine/metabolic | SMD | 1.0 | 0.90 (0.72 - 1.12) | 129 | 14755 |
| 574.12 | Cholelithiasis with other cholecystitis | digestive | SMI | 1.0 | 1.09 (0.92 - 1.28) | 166 | 15847 |
| 155.1 | Malignant neoplasm of liver, primary | neoplasms | SMD | 1.0 | 0.93 (0.79 - 1.08) | 254 | 15023 |
| 250.42 | Other abnormal glucose | endocrine/metabolic | SMI | 1.0 | 1.05 (0.95 - 1.15) | 548 | 14753 |
| 332 | Parkinson's disease | neurological | SMI | 1.0 | 0.89 (0.70 - 1.13) | 113 | 16355 |
| 440 | Atherosclerosis | circulatory system | SMI | 1.0 | 0.91 (0.75 - 1.10) | 145 | 16724 |
| 850 | Hemorrhage or hematoma complicating a procedure | injuries & poisonings | SMI | 1.0 | 0.92 (0.77 - 1.09) | 149 | 16976 |
| 293.1 | Swelling, mass, or lump in head and neck [Space-occupying lesion, intracranial NOS] | mental disorders | SMI | 1.0 | 0.91 (0.76 - 1.10) | 131 | 17506 |
| 965.1 | Opiates and related narcotics causing adverse effects in therapeutic use | injuries & poisonings | SMI | 1.0 | 0.93 (0.80 - 1.08) | 210 | 15992 |
| 362 | Other retinal disorders | sense organs | SMI | 1.0 | 0.94 (0.83 - 1.07) | 340 | 17005 |
| 618 | Genital prolapse | genitourinary | SMI | 1.0 | 1.09 (0.90 - 1.33) | 115 | 10039 |
| 257 | Testicular dysfunction | endocrine/metabolic | SMD | 1.0 | 0.90 (0.71 - 1.14) | 102 | 7159 |
| 519.9 | Symptoms involving respiratory system and other chest symptoms | respiratory | SMD | 1.0 | 0.91 (0.75 - 1.12) | 150 | 17368 |
| 241.1 | Nontoxic uninodular goiter | endocrine/metabolic | SMI | 1.0 | 1.09 (0.90 - 1.32) | 122 | 16103 |
| 476 | Allergic rhinitis | respiratory | SMI | 1.0 | 1.06 (0.93 - 1.21) | 250 | 16763 |
| 241 | Nontoxic nodular goiter | endocrine/metabolic | SMI | 1.0 | 1.07 (0.92 - 1.26) | 187 | 16103 |
| 433.21 | Cerebral artery occlusion, with cerebral infarction | circulatory system | SMI | 1.0 | 0.94 (0.81 - 1.08) | 255 | 16761 |
| 411 | Ischemic Heart Disease | circulatory system | SMI | 1.0 | 0.97 (0.91 - 1.04) | 1325 | 16271 |
| 433.2 | Occlusion of cerebral arteries | circulatory system | SMI | 1.0 | 0.94 (0.82 - 1.08) | 260 | 16761 |
| 705.8 | Hyperhidrosis | dermatologic | SMI | 1.0 | 1.08 (0.90 - 1.31) | 122 | 17313 |
| 185 | Cancer of prostate | neoplasms | SMD | 1.0 | 0.95 (0.84 - 1.07) | 483 | 6259 |
| 840 | Sprains and strains | injuries & poisonings | SMD | 1.0 | 1.10 (0.88 - 1.39) | 120 | 17447 |
| 159.2 | Malignant neoplasm of small intestine, including duodenum | neoplasms | SMD | 1.0 | 0.91 (0.73 - 1.14) | 117 | 15023 |
| 593.1 | Gross hematuria | genitourinary | SMD | 1.0 | 0.91 (0.73 - 1.14) | 127 | 15562 |
| 375.1 | Dry eyes | sense organs | SMD | 1.0 | 1.10 (0.88 - 1.38) | 123 | 17157 |
| 805 | Fracture of vertebral column without mention of spinal cord injury | injuries & poisonings | SMD | 1.0 | 0.94 (0.81 - 1.09) | 280 | 16753 |
| 174 | Breast cancer | neoplasms | SMD | 1.0 | 0.96 (0.86 - 1.07) | 570 | 16343 |
| 415.2 | Chronic pulmonary heart disease | circulatory system | SMI | 1.0 | 0.94 (0.81 - 1.09) | 227 | 16494 |
| 605 | Erectile dysfunction [ED] | genitourinary | SMD | 1.0 | 1.09 (0.88 - 1.34) | 147 | 6632 |
| 244 | Hypothyroidism | endocrine/metabolic | SMI | 1.0 | 1.03 (0.96 - 1.10) | 1245 | 16103 |
| 788 | Syncope and collapse | symptoms | SMD | 1.0 | 1.05 (0.93 - 1.19) | 399 | 17240 |
| 158 | Neoplasm of unspecified nature of digestive system | neoplasms | SMD | 1.0 | 0.94 (0.79 - 1.11) | 207 | 15023 |
| 530.14 | Reflux esophagitis | digestive | SMD | 1.0 | 1.07 (0.90 - 1.26) | 226 | 14896 |
| 702.2 | Seborrheic keratosis | dermatologic | SMI | 1.0 | 1.06 (0.90 - 1.25) | 194 | 17320 |
| 365 | Glaucoma | sense organs | SMI | 1.0 | 0.95 (0.81 - 1.10) | 238 | 17050 |
| 701 | Other hypertrophic and atrophic conditions of skin | dermatologic | SMD | 1.0 | 0.94 (0.80 - 1.11) | 239 | 17373 |
| 340 | Migraine | neurological | SMD | 1.0 | 0.95 (0.84 - 1.08) | 403 | 16885 |
| 743.9 | Osteopenia or other disorder of bone and cartilage | musculoskeletal | SMD | 1.0 | 0.95 (0.81 - 1.10) | 265 | 16912 |
| 530.14 | Reflux esophagitis | digestive | SMI | 1.0 | 0.95 (0.82 - 1.10) | 226 | 14903 |
| 339 | Other headache syndromes | neurological | SMI | 1.0 | 1.04 (0.93 - 1.15) | 407 | 16892 |
| 443.9 | Peripheral vascular disease, unspecified | circulatory system | SMI | 1.0 | 1.06 (0.89 - 1.26) | 177 | 16724 |
| 216 | Benign neoplasm of skin | neoplasms | SMI | 1.0 | 0.95 (0.82 - 1.10) | 197 | 17329 |
| 535.9 | Gastritis and duodenitis, NOS | digestive | SMI | 1.0 | 0.95 (0.81 - 1.11) | 182 | 16078 |
| 366 | Cataract | sense organs | SMD | 1.0 | 0.96 (0.86 - 1.08) | 526 | 17113 |
| 479 | Other upper respiratory disease | respiratory | SMD | 1.0 | 0.95 (0.81 - 1.11) | 254 | 16757 |
| 350.2 | Abnormality of gait | neurological | SMD | 1.0 | 0.94 (0.78 - 1.13) | 182 | 17242 |
| 599 | Other symptoms/disorders or the urinary system | genitourinary | SMI | 1.0 | 0.98 (0.93 - 1.04) | 1481 | 15864 |
| 722.6 | Degeneration of intervertebral disc | musculoskeletal | SMI | 1.0 | 0.95 (0.80 - 1.12) | 185 | 16756 |
| 371.3 | Inflammation of eyelids | sense organs | SMD | 1.0 | 1.08 (0.85 - 1.38) | 106 | 17301 |
| 170 | Cancer of bone and connective tissue | neoplasms | SMD | 1.0 | 0.95 (0.83 - 1.10) | 300 | 17339 |
| 380.4 | Impacted cerumen | sense organs | SMI | 1.0 | 1.07 (0.86 - 1.33) | 109 | 17513 |
| 578.8 | Hemorrhage of rectum and anus | digestive | SMI | 1.0 | 1.04 (0.92 - 1.18) | 277 | 15485 |
| 562 | Diverticulosis and diverticulitis | digestive | SMD | 1.0 | 0.98 (0.91 - 1.05) | 1487 | 11799 |
| 721 | Spondylosis and allied disorders | musculoskeletal | SMI | 1.0 | 1.04 (0.92 - 1.17) | 370 | 16756 |
| 809 | Fracture of unspecified bones | injuries & poisonings | SMD | 1.0 | 0.96 (0.85 - 1.09) | 379 | 16753 |
| 216.1 | Screening for malignant neoplasms of the skin | neoplasms | SMI | 1.0 | 1.06 (0.87 - 1.28) | 125 | 17329 |
| 250.41 | Impaired fasting glucose | endocrine/metabolic | SMD | 1.0 | 0.94 (0.76 - 1.16) | 143 | 14747 |
| 172.2 | Other non-epithelial cancer of skin | neoplasms | SMD | 1.0 | 1.05 (0.90 - 1.22) | 286 | 17153 |
| 172 | Skin cancer | neoplasms | SMD | 1.0 | 0.96 (0.85 - 1.10) | 388 | 17153 |
| 800 | Fracture of lower limb | injuries & poisonings | SMI | 1.0 | 0.96 (0.82 - 1.11) | 207 | 16757 |
| 443 | Peripheral vascular disease | circulatory system | SMI | 1.0 | 0.96 (0.82 - 1.12) | 226 | 16724 |
| 530.1 | Esophagitis, GERD and related diseases | digestive | SMI | 1.0 | 0.99 (0.94 - 1.04) | 2098 | 14903 |
| 721.1 | Spondylosis without myelopathy | musculoskeletal | SMI | 1.0 | 1.03 (0.91 - 1.17) | 334 | 16756 |
| 705.8 | Hyperhidrosis | dermatologic | SMD | 1.0 | 1.06 (0.85 - 1.34) | 122 | 17306 |
| 455 | Hemorrhoids | circulatory system | SMI | 1.0 | 1.03 (0.93 - 1.14) | 464 | 15244 |
| 110 | Dermatophytosis / Dermatomycosis | infectious diseases | SMI | 1.0 | 1.04 (0.89 - 1.21) | 216 | 16940 |
| 300.9 | Posttraumatic stress disorder | mental disorders | SMI | 1.0 | 1.05 (0.86 - 1.28) | 106 | 14166 |
| 374 | Other disorders of eyelids | sense organs | SMI | 1.0 | 1.05 (0.85 - 1.30) | 110 | 17308 |
| 496.2 | Chronic bronchitis | respiratory | SMI | 1.0 | 1.05 (0.85 - 1.29) | 122 | 16329 |
| 241 | Nontoxic nodular goiter | endocrine/metabolic | SMD | 1.0 | 1.04 (0.87 - 1.26) | 187 | 16096 |
| 170.2 | Cancer of connective tissue | neoplasms | SMD | 1.0 | 0.96 (0.83 - 1.12) | 269 | 17339 |
| 318 | Tobacco use disorder | mental disorders | SMI | 1.0 | 1.01 (0.96 - 1.07) | 1555 | 15504 |
| 332 | Parkinson's disease | neurological | SMD | 1.0 | 1.06 (0.83 - 1.35) | 113 | 16348 |
| 157 | Pancreatic cancer | neoplasms | SMD | 1.0 | 0.97 (0.86 - 1.10) | 440 | 15023 |
| 159.4 | Malignant neoplasm of retroperitoneum and peritoneum | neoplasms | SMD | 1.0 | 1.05 (0.85 - 1.31) | 130 | 15023 |
| 389.1 | Sensorineural hearing loss | sense organs | SMI | 1.0 | 1.04 (0.88 - 1.22) | 198 | 17258 |
| 279.7 | Other immunological findings | endocrine/metabolic | SMD | 1.0 | 1.06 (0.83 - 1.36) | 102 | 17077 |
| 306 | Other mental disorder | mental disorders | SMI | 1.0 | 0.97 (0.86 - 1.10) | 317 | 14166 |
| 292.3 | Memory loss | mental disorders | SMD | 1.0 | 0.95 (0.75 - 1.21) | 112 | 16099 |
| 165 | Cancer within the respiratory system | neoplasms | SMD | 1.0 | 0.97 (0.82 - 1.14) | 233 | 17396 |
| 722 | Intervertebral disc disorders | musculoskeletal | SMI | 1.0 | 0.97 (0.86 - 1.10) | 338 | 16756 |
| 550.2 | Diaphragmatic hernia | digestive | SMI | 1.0 | 0.98 (0.86 - 1.10) | 344 | 16384 |
| 722.1 | Displacement of intervertebral disc | musculoskeletal | SMD | 1.0 | 1.05 (0.83 - 1.34) | 105 | 16752 |
| 365 | Glaucoma | sense organs | SMD | 1.0 | 0.97 (0.82 - 1.14) | 238 | 17043 |
| 702 | Degenerative skin conditions and other dermatoses | dermatologic | SMI | 1.0 | 1.03 (0.90 - 1.16) | 326 | 17106 |
| 619.4 | Noninflammatory disorders of vagina | genitourinary | SMD | 1.0 | 0.96 (0.78 - 1.18) | 147 | 9596 |
| 367 | Disorders of refraction and accommodation; blindness and low vision | sense organs | SMI | 1.0 | 0.97 (0.85 - 1.12) | 251 | 17395 |
| 173 | Neoplasm of uncertain behavior of skin | neoplasms | SMD | 1.0 | 0.96 (0.79 - 1.17) | 164 | 17153 |
| 211 | Benign neoplasm of other parts of digestive system | neoplasms | SMI | 1.0 | 1.03 (0.88 - 1.22) | 168 | 15141 |
| 274.1 | Gout | endocrine/metabolic | SMI | 1.0 | 1.03 (0.87 - 1.22) | 184 | 17444 |
| 271.3 | Intestinal disaccharidase deficiencies and disaccharide malabsorption | endocrine/metabolic | SMD | 1.0 | 1.05 (0.83 - 1.33) | 113 | 17523 |
| 187 | Cancer of other male genital organs | neoplasms | SMD | 1.0 | 1.04 (0.87 - 1.25) | 189 | 6824 |
| 292.3 | Memory loss | mental disorders | SMI | 1.0 | 1.04 (0.84 - 1.30) | 112 | 16104 |
| 427.9 | Palpitations | circulatory system | SMD | 1.0 | 0.97 (0.82 - 1.15) | 224 | 12600 |
| 698 | Pruritus and related conditions | dermatologic | SMI | 1.0 | 0.97 (0.84 - 1.12) | 213 | 17433 |
| 579.8 | Nonspecific abnormal findings in stool contents | digestive | SMI | 1.0 | 1.03 (0.89 - 1.18) | 237 | 15485 |
| 574.11 | Cholelithiasis with acute cholecystitis | digestive | SMI | 1.0 | 1.04 (0.85 - 1.27) | 116 | 15847 |
| 110.11 | Dermatophytosis of nail | infectious diseases | SMD | 1.0 | 0.96 (0.76 - 1.22) | 111 | 16933 |
| 585.4 | Chronic kidney disease, Stage I or II | genitourinary | SMI | 1.0 | 1.04 (0.83 - 1.30) | 102 | 14825 |
| 271 | Disorders of carbohydrate transport and metabolism | endocrine/metabolic | SMD | 1.0 | 1.04 (0.83 - 1.31) | 116 | 17523 |
| 172.2 | Other non-epithelial cancer of skin | neoplasms | SMI | 1.0 | 0.98 (0.85 - 1.12) | 286 | 17160 |
| 465.2 | Acute pharyngitis | respiratory | SMI | 1.0 | 0.97 (0.82 - 1.16) | 137 | 17234 |
| 512.8 | Cough | respiratory | SMI | 1.0 | 1.01 (0.94 - 1.10) | 807 | 15037 |
| 458.1 | Orthostatic hypotension | circulatory system | SMD | 1.0 | 0.96 (0.76 - 1.22) | 109 | 16641 |
| 394 | Rheumatic disease of the heart valves | circulatory system | SMI | 1.0 | 0.97 (0.79 - 1.19) | 123 | 17035 |
| 702.1 | Actinic keratosis | dermatologic | SMD | 1.0 | 1.03 (0.86 - 1.24) | 188 | 17313 |
| 269 | Proteinuria | endocrine/metabolic | SMI | 1.0 | 1.03 (0.84 - 1.27) | 103 | 17328 |
| 475 | Chronic sinusitis | respiratory | SMI | 1.0 | 1.02 (0.88 - 1.19) | 194 | 16763 |
| 159.4 | Malignant neoplasm of retroperitoneum and peritoneum | neoplasms | SMI | 1.0 | 0.97 (0.80 - 1.18) | 130 | 15030 |
| 593 | Hematuria | genitourinary | SMI | 1.0 | 0.99 (0.89 - 1.09) | 453 | 15566 |
| 244.4 | Hypothyroidism NOS | endocrine/metabolic | SMI | 1.0 | 1.01 (0.94 - 1.08) | 1116 | 16103 |
| 465 | Acute upper respiratory infections of multiple or unspecified sites | respiratory | SMD | 1.0 | 0.98 (0.85 - 1.13) | 319 | 17227 |
| 790 | Nonspecific findings on examination of blood | symptoms | SMI | 1.0 | 1.03 (0.85 - 1.24) | 136 | 16697 |
| 362.2 | Degeneration of macula and posterior pole of retina | sense organs | SMI | 1.0 | 0.98 (0.83 - 1.15) | 223 | 17005 |
| 371 | Inflammation of the eye | sense organs | SMD | 1.0 | 1.03 (0.84 - 1.25) | 158 | 17301 |
| 726 | Peripheral enthesopathies and allied syndromes | musculoskeletal | SMD | 1.0 | 0.98 (0.83 - 1.16) | 218 | 16966 |
| 803 | Fracture of upper limb | injuries & poisonings | SMI | 1.0 | 0.98 (0.83 - 1.16) | 156 | 16757 |
| 687 | Symptoms affecting skin | dermatologic | SMI | 1.0 | 1.02 (0.85 - 1.23) | 133 | 16836 |
| 362 | Other retinal disorders | sense organs | SMD | 1.0 | 0.98 (0.86 - 1.13) | 340 | 16998 |
| 386.9 | Dizziness and giddiness (Light-headedness and vertigo) | sense organs | SMI | 1.0 | 0.99 (0.91 - 1.08) | 634 | 16972 |
| 743 | Osteoporosis, osteopenia and pathological fracture | musculoskeletal | SMD | 1.0 | 0.99 (0.90 - 1.09) | 727 | 16912 |
| 514.2 | Solitary pulmonary nodule | respiratory | SMD | 1.0 | 0.98 (0.86 - 1.12) | 385 | 14743 |
| 560.3 | Peritoneal or intestinal adhesions | digestive | SMD | 1.0 | 1.03 (0.83 - 1.26) | 142 | 11799 |
| 726.1 | Enthesopathy | musculoskeletal | SMD | 1.0 | 0.97 (0.77 - 1.23) | 112 | 16966 |
| 574 | Cholelithiasis and cholecystitis | digestive | SMI | 1.0 | 1.01 (0.94 - 1.08) | 1050 | 15847 |
| 751.2 | Congenital anomalies of urinary system | congenital anomalies | SMD | 1.0 | 1.03 (0.82 - 1.30) | 113 | 17405 |
| 687.4 | Disturbance of skin sensation | dermatologic | SMI | 1.0 | 0.99 (0.88 - 1.11) | 320 | 16836 |
| 165.1 | Cancer of bronchus; lung | neoplasms | SMD | 1.0 | 0.98 (0.83 - 1.16) | 225 | 17396 |
| 395.2 | Nonrheumatic aortic valve disorders | circulatory system | SMI | 1.0 | 0.98 (0.82 - 1.17) | 198 | 17035 |
| 244.2 | Acquired hypothyroidism | endocrine/metabolic | SMI | 1.0 | 1.02 (0.83 - 1.26) | 108 | 16103 |
| 704 | Diseases of hair and hair follicles | dermatologic | SMI | 1.0 | 0.98 (0.82 - 1.17) | 134 | 17407 |
| 427.9 | Palpitations | circulatory system | SMI | 1.0 | 1.02 (0.88 - 1.17) | 224 | 12606 |
| 371.3 | Inflammation of eyelids | sense organs | SMI | 1.0 | 1.02 (0.83 - 1.26) | 106 | 17308 |
| 599.4 | Urinary incontinence | genitourinary | SMI | 1.0 | 1.01 (0.89 - 1.16) | 283 | 15864 |
| 614.5 | Inflammatory disease of cervix, vagina, and vulva | genitourinary | SMD | 1.0 | 1.02 (0.82 - 1.29) | 123 | 9617 |
| 374 | Other disorders of eyelids | sense organs | SMD | 1.0 | 0.98 (0.77 - 1.24) | 110 | 17301 |
| 173 | Neoplasm of uncertain behavior of skin | neoplasms | SMI | 1.0 | 0.98 (0.83 - 1.17) | 164 | 17160 |
| 151 | Cancer of stomach | neoplasms | SMD | 1.0 | 1.02 (0.86 - 1.20) | 234 | 15023 |
| 209 | Neuroendocrine tumors | neoplasms | SMD | 1.0 | 1.02 (0.85 - 1.21) | 199 | 17440 |
| 535 | Gastritis and duodenitis | digestive | SMI | 1.0 | 0.99 (0.91 - 1.08) | 608 | 16078 |
| 389.1 | Sensorineural hearing loss | sense organs | SMD | 1.0 | 1.02 (0.85 - 1.22) | 197 | 17252 |
| 379 | Other disorders of eye | sense organs | SMD | 1.0 | 1.01 (0.86 - 1.19) | 248 | 17157 |
| 371 | Inflammation of the eye | sense organs | SMI | 1.0 | 0.99 (0.83 - 1.17) | 158 | 17308 |
| 694.2 | Other dyschromia | dermatologic | SMD | 1.0 | 1.02 (0.82 - 1.26) | 134 | 16949 |
| 687.4 | Disturbance of skin sensation | dermatologic | SMD | 1.0 | 1.01 (0.88 - 1.16) | 318 | 16831 |
| 819 | Skull and face fracture and other intercranial injury | injuries & poisonings | SMD | 1.0 | 1.02 (0.80 - 1.30) | 106 | 17488 |
| 593 | Hematuria | genitourinary | SMD | 1.0 | 1.01 (0.90 - 1.13) | 453 | 15562 |
| 694 | Dyschromia and Vitiligo | dermatologic | SMD | 1.0 | 1.01 (0.83 - 1.24) | 149 | 16949 |
| 727 | Other disorders of synovium, tendon, and bursa | musculoskeletal | SMD | 1.0 | 0.99 (0.82 - 1.20) | 171 | 16966 |
| 351 | Other peripheral nerve disorders | neurological | SMI | 1.0 | 1.01 (0.88 - 1.15) | 254 | 17143 |
| 807 | Fracture of ribs | injuries & poisonings | SMI | 1.0 | 1.01 (0.85 - 1.20) | 154 | 16757 |
| 593.1 | Gross hematuria | genitourinary | SMI | 1.0 | 0.99 (0.81 - 1.21) | 127 | 15566 |
| 809 | Fracture of unspecified bones | injuries & poisonings | SMI | 1.0 | 1.00 (0.89 - 1.11) | 380 | 16757 |
| 250.1 | Type 1 diabetes | endocrine/metabolic | SMI | 1.0 | 0.99 (0.84 - 1.18) | 147 | 14753 |
| 375.1 | Dry eyes | sense organs | SMI | 1.0 | 1.01 (0.83 - 1.22) | 123 | 17164 |
| 429 | Ill-defined descriptions and complications of heart disease | circulatory system | SMI | 1.0 | 1.01 (0.82 - 1.24) | 116 | 16709 |
| 599.5 | Frequency of urination and polyuria | genitourinary | SMD | 1.0 | 1.00 (0.88 - 1.13) | 422 | 15860 |
| 433.1 | Occlusion and stenosis of precerebral arteries | circulatory system | SMI | 1.0 | 1.01 (0.82 - 1.24) | 127 | 16761 |
| 702.1 | Actinic keratosis | dermatologic | SMI | 1.0 | 1.00 (0.84 - 1.18) | 188 | 17320 |
| 590 | Pyelonephritis | genitourinary | SMI | 1.0 | 1.00 (0.90 - 1.11) | 428 | 15566 |
| 272.11 | Hypercholesterolemia | endocrine/metabolic | SMD | 1.0 | 1.00 (0.87 - 1.16) | 310 | 14755 |
| 599.3 | Dysuria | genitourinary | SMD | 1.0 | 1.00 (0.90 - 1.11) | 583 | 15860 |
| 475 | Chronic sinusitis | respiratory | SMD | 1.0 | 1.00 (0.83 - 1.19) | 194 | 16757 |
| 389 | Hearing loss | sense organs | SMD | 1.0 | 1.00 (0.88 - 1.14) | 385 | 17252 |
| 704 | Diseases of hair and hair follicles | dermatologic | SMD | 1.0 | 1.00 (0.81 - 1.24) | 134 | 17400 |
| 751 | Genitourinary congenital anomalies | congenital anomalies | SMD | 1.0 | 1.00 (0.82 - 1.23) | 147 | 17405 |
| 619.4 | Noninflammatory disorders of vagina | genitourinary | SMI | 1.0 | 1.00 (0.84 - 1.18) | 147 | 9601 |
| 070.3 | Viral hepatitis C | infectious diseases | SMI | 1.0 | 1.00 (0.89 - 1.12) | 359 | 16473 |
| 464 | Acute sinusitis | respiratory | SMD | 1.0 | 1.00 (0.79 - 1.27) | 109 | 17227 |
| 274 | Gout and other crystal arthropathies | endocrine/metabolic | SMI | 1.0 | 1.00 (0.85 - 1.18) | 202 | 17444 |
| 366.2 | Senile cataract | sense organs | SMD | 1.0 | 1.00 (0.86 - 1.16) | 272 | 17113 |
| 724 | Other and unspecified disorders of back | musculoskeletal | SMI | 1.0 | 1.00 (0.83 - 1.19) | 143 | 16756 |
| 790 | Nonspecific findings on examination of blood | symptoms | SMD | 1.0 | 1.00 (0.81 - 1.24) | 136 | 16690 |
| 562.1 | Diverticulosis | digestive | SMD | 1.0 | 1.00 (0.92 - 1.09) | 886 | 11799 |

Note.– SMI = skeletal muscle index, SMD = skeletal muscle density, OR = sex and age adjusted odds ratio, CI = confidence interval, P-value = Bonferroni adjusted p value.

Table S2. Number of associations between SMI, SMD and medical phenotypes in cases where nonlinear model represents a better fit compared to linear model.

| **Measurement** | **Significant in Linear Model** | **Significant in Nonlinear Model** | | | |
| --- | --- | --- | --- | --- | --- |
|  |  | **Linear term only** | **Square term only** | **Both linear and square term** |  |
| SMI | No | 0 | 8 | 0 |  |
|  | Yes | 0 | 2 | 19 |  |
| SMD | No | 0 | 6 | 6 |  |
|  | Yes | 2 | 0 | 67 |  |

Note.– SMI= skeletal muscle index, SMD=skeletal muscle density.

Table S3: Associations between SMI, SMD and medical phenotypes in cases where nonlinear model represents a better fit compared to linear model and linear only model had no statistically significant association.

| **Phenotype** | **Description** | **Phenotype Group** | **Metric** | **P value** | **OR (95% CI)** | **Cases (n)** | **Controls (n)** |
| --- | --- | --- | --- | --- | --- | --- | --- |
| 070 | Viral hepatitis | infectious diseases | SMD | 1.0 | 0.81 (0.71 - 0.92) | 570 | 16466 |
| 070 | Viral hepatitis | infectious diseases | SMD² | < 0.0001 | 0.77 (0.70 - 0.84) | 570 | 16466 |
| 070.3 | Viral hepatitis C | infectious diseases | SMD | < 0.0001 | 0.62 (0.52 - 0.73) | 359 | 16466 |
| 070.3 | Viral hepatitis C | infectious diseases | SMD² | < 0.0001 | 0.72 (0.63 - 0.81) | 359 | 16466 |
| 153 | Colorectal cancer | neoplasms | SMD | 0.0054 | 0.80 (0.72 - 0.88) | 1002 | 14710 |
| 153 | Colorectal cancer | neoplasms | SMD² | 0.0011 | 0.86 (0.81 - 0.91) | 1002 | 14710 |
| 153.3 | Malignant neoplasm of rectum, rectosigmoid junction, and anus | neoplasms | SMD | 0.043 | 0.75 (0.66 - 0.86) | 506 | 14710 |
| 153.3 | Malignant neoplasm of rectum, rectosigmoid junction, and anus | neoplasms | SMD² | 0.0018 | 0.80 (0.73 - 0.87) | 506 | 14710 |
| 155 | Cancer of liver and intrahepatic bile duct | neoplasms | SMD | 0.031 | 0.69 (0.58 - 0.82) | 391 | 15023 |
| 155 | Cancer of liver and intrahepatic bile duct | neoplasms | SMD² | 0.0062 | 0.77 (0.69 - 0.86) | 391 | 15023 |
| 155.1 | Malignant neoplasm of liver, primary | neoplasms | SMD | 1.0 | 0.71 (0.57 - 0.87) | 254 | 15023 |
| 155.1 | Malignant neoplasm of liver, primary | neoplasms | SMD² | 0.0062 | 0.70 (0.60 - 0.81) | 254 | 15023 |
| 157 | Pancreatic cancer | neoplasms | SMD | 1.0 | 0.78 (0.66 - 0.91) | 440 | 15023 |
| 157 | Pancreatic cancer | neoplasms | SMD² | 0.0029 | 0.77 (0.69 - 0.86) | 440 | 15023 |
| 159 | Malignant neoplasm of other and ill-defined sites within the digestive organs and peritoneum | neoplasms | SMD | 0.093 | 0.76 (0.67 - 0.87) | 627 | 15023 |
| 159 | Malignant neoplasm of other and ill-defined sites within the digestive organs and peritoneum | neoplasms | SMD² | < 0.0001 | 0.74 (0.68 - 0.81) | 627 | 15023 |
| 170 | Cancer of bone and connective tissue | neoplasms | SMD | 1.0 | 0.85 (0.72 - 1.01) | 300 | 17339 |
| 170 | Cancer of bone and connective tissue | neoplasms | SMD² | 0.0040 | 0.73 (0.64 - 0.83) | 300 | 17339 |
| 170.2 | Cancer of connective tissue | neoplasms | SMD | 1.0 | 0.86 (0.71 - 1.03) | 269 | 17339 |
| 170.2 | Cancer of connective tissue | neoplasms | SMD² | 0.0075 | 0.72 (0.62 - 0.83) | 269 | 17339 |
| 198.1 | Secondary malignancy of lymph nodes | neoplasms | SMD | < 0.0001 | 0.66 (0.58 - 0.75) | 612 | 14136 |
| 198.1 | Secondary malignancy of lymph nodes | neoplasms | SMD² | < 0.0001 | 0.72 (0.65 - 0.79) | 612 | 14136 |
| 575.1 | Cholangitis | digestive | SMD | 0.00070 | 0.61 (0.50 - 0.73) | 291 | 15841 |
| 575.1 | Cholangitis | digestive | SMD² | 0.036 | 0.76 (0.66 - 0.86) | 291 | 15841 |
| 250.3 | Insulin pump user | endocrine/metabolic | SMI | 1.0 | 1.06 (0.97 - 1.15) | 487 | 14753 |
| 250.3 | Insulin pump user | endocrine/metabolic | SMI² | 0.0012 | 1.09 (1.05 - 1.13) | 487 | 14753 |
| 318 | Tobacco use disorder | mental disorders | SMI | 1.0 | 1.01 (0.95 - 1.06) | 1555 | 15504 |
| 318 | Tobacco use disorder | mental disorders | SMI² | 0.047 | 1.06 (1.03 - 1.08) | 1555 | 15504 |
| 338.2 | Chronic pain | neurological | SMI | 1.0 | 0.94 (0.89 - 1.00) | 1089 | 15508 |
| 338.2 | Chronic pain | neurological | SMI² | 0.024 | 1.06 (1.03 - 1.09) | 1089 | 15508 |
| 512.7 | Shortness of breath | respiratory | SMI | 0.56 | 0.91 (0.86 - 0.96) | 1556 | 15037 |
| 512.7 | Shortness of breath | respiratory | SMI² | 0.014 | 1.06 (1.03 - 1.09) | 1556 | 15037 |
| 760 | Back pain | symptoms | SMI | 1.0 | 1.03 (0.97 - 1.08) | 1500 | 16146 |
| 760 | Back pain | symptoms | SMI² | < 0.0001 | 1.08 (1.06 - 1.11) | 1500 | 16146 |
| 764 | Sciatica | symptoms | SMI | 1.0 | 1.15 (1.03 - 1.30) | 243 | 17097 |
| 764 | Sciatica | symptoms | SMI² | 0.024 | 1.11 (1.05 - 1.16) | 243 | 17097 |
| 800 | Fracture of lower limb | injuries & poisonings | SMI | 1.0 | 0.96 (0.84 - 1.09) | 207 | 16757 |
| 800 | Fracture of lower limb | injuries & poisonings | SMI² | 0.020 | 1.12 (1.06 - 1.17) | 207 | 16757 |
| 871 | Open wounds of extremities | injuries & poisonings | SMI | 1.0 | 0.89 (0.78 - 1.02) | 153 | 17226 |
| 871 | Open wounds of extremities | injuries & poisonings | SMI² | 0.011 | 1.12 (1.06 - 1.17) | 153 | 17226 |

Note.– SMI = skeletal muscle index, SMD = skeletal muscle density, OR = sex and age adjusted odds ratio, CI = confidence interval, P-value = Bonferroni adjusted p value.
